# Variation in the Analysis of Positively Selected Sites Using Nonsynonymous/Synonymous Rate Ratios: An Example Using Influenza Virus

**DOI:** 10.1371/journal.pone.0019996

**Published:** 2011-05-24

**Authors:** Jiming Chen, Yingxue Sun

**Affiliations:** China Animal Health and Epidemiology Center, Qingdao, China; University of Cambridge, United Kingdom

## Abstract

Sites in a gene showing the nonsynonymous/synonymous rate ratio (ω) >1 have been frequently identified to be under positive selection. To examine the performance of such analysis, sites of the ω ratio >1 in the HA1 gene of H3N2 subtype human influenza viruses were identified from seven overlapping sequence data sets in this study. Our results showed that the sites of the ω ratio >1 were of significant variation among the data sets even though they targeted similar clusters, indicating that the analysis is likely to be either of low sensitivity or of low specificity in identifying sites under positive selection. Most (43/45) of the sites showing ω >1 calculated from at least one data set are involved in B-cell epitopes which cover less than a half sites in the protein, suggesting that the analysis is likely to be of low sensitivity rather than of low specificity. It was further found that the analysis sensitivity could not be enhanced by including more sequences or covering longer time intervals. Previously some reports also likely identified only a portion of the sites under positive selection in the viral gene using the ω ratio. Low sensitivity of the analysis may result from that some sites under positive selection in the gene are also under negative (purifying) selection simultaneously for functional constrains, and so their ω ratios could be <1. Theoretically, the sites under the two opposite selection forces at the same time favor only certain nonsynonymous changes, e.g. those changing the antigenicity of the gene and maintaining the gene function. This study also suggested that sometimes we can identify more sites under positive selection using the ω ratio by integrating the positively selected sites estimated from multiple data sets.

## Introduction

Molecular selection modulates gene sequence evolution in different ways by constraining potential changes in amino acid sequences (negative or purifying selection) or by favoring new and adaptive genetic variants (positive selection), and is ultimately responsible for adaption in morphology and behavior [1]–[4]. It is thus an exciting topic in molecular evolutionary studies. The ratio (ω) of the synonymous substitution rate (dS) versus the nonsynonymous substitution rate (dN) is widely used as an indicator of selective pressure acting on a protein-coding gene [3], [4]. Homologous genes of the ω ratio = 1, <1 or >1 are usually assumed to be evolving under neutral evolution, negative selection or positive selection, respectively.

Estimation of the ω ratio is very complicated [2], [3]. The ratio was estimated in early studies by taking an average of all sites in a gene. Because the sites in a gene under negative selection due to functional constraints are usually more than those under positive selection, such analysis can rarely find ω ratios >1 or detect positive selection [1], [3], [5]. Later, methods were developed to estimate the ω ratio at each site of a gene by reconstructing ancestral sequences via counting synonymous and nonsynonymous changes along the tree at each site using the maximum parsimony method [6]–[8], or maximum likelihood (ML) methods based on explicit models of codon substitution assuming variable ω ratios among sites [9], [10]. Such methods have been thought to be powerful in identifying sites under positive selection in genes of viruses, bacteria and other organisms including mammals [6]–[15].

Although the ω ratio has been widely used in identifying sites under positive selection in a gene, the specificity and sensitivity of the analysis remains unclear because it is difficult to predict exactly which sites are under positive selection [3], [16], [17]. Some researchers have noted that this measure may be of low sensitivity in the analysis of positive selection if the compared sequences are sampled from a single population [4]. Extensive variation in the ω ratios has also been demonstrated when comparing closely related bacterial genomes, and hitch-hiking may play a role in this variation [18]. In addition, empirical data based on whole-genome protein sequence alignments between humans and fifteen other vertebrate species suggested that the ω ratios do not appropriately reflect the action of selection as they are strongly influenced by the denominator dS [19].

In this study, we estimated sites under positive selection in the HA1 gene of H3N2 human influenza viruses using the ω ratios, and examined the specificity and the sensitivity of this measure. The HA1 gene encodes the major surface protein harboring five B-cell epitopes (BCEs), epitopes A–E, for the binding of neutralizing antibodies which are induced after infection or vaccination [7]. Nonsynonymous substitutions at BCEs may help the virus avoid host immunity, allowing them to be positively selected. Another advantage of investigating this viral gene is that high-quality sequences of this gene are available for each year over recent decades. Therefore, this gene is a good candidate to study the performance of the ω ratio as an indicator of selection pressure.

## Materials and Methods

### Sequence data sets

Seven overlapping sequence data sets were used in this study. They covered 43, 43, 86, 15, 15, 30 and 30 sequences, respectively. Data set 1 was built by randomly selecting one full-length sequence of the viral HA1 gene with no ambiguous nucleotides for each of the years 1968–2010. Data set 2 was built by randomly selecting another full-length sequence of the viral HA1 gene with no ambiguous nucleotides for each of the years 1968–2010. Data set 3 consisted of data set 1 and data set 2. Data set 4 was the part of data set 1 covering the years 1983–1997. Data set 5 was the part of data set 2 covering the years 1983–1997. Data set 6 was the part of data set 3 covering the years 1996–2010. Data set 7 was the part of data set 3 covering the years 1983–1997. These data sets were used to examine whether more sequences or sequences covering longer time intervals increase the sensitivity of the measure. Previous reports using the same analyses were based on the period 1983–1997 [6], [7], [12], and this period was also selected in this study to allow comparison of the results. Additionally, sequences from 1996–2010 were also selected here to compare the two time periods. All the sequences were selected from GenBank and given in Text S1.

### Phylogenetic analysis

Sequences were aligned using the software MUSCLE [20], and their phylogenetic relationships were calculated by the maximum likelihood (ML) method using the software MEGA 5b [21]. The nucleotide substitution was set in the General Time Reversible (GTR) model. Rates among site were set in a gamma distribution with invariant sites (G+I), and the number of discrete gamma categories was set as 4.

### Calculation of the ω ratio

The ω ratio of each site in the viral gene was estimated using the software PAML 4.4 [22]. Codon frequencies were set as the F3×4 table. The ω ratios were analyzed under the following six models, most of which allow ω ratios variable among sites [3], [10], [22]. Model M0 (one-ratio) assumes one ω for all sites. Model M1 (neutral) assumes a class of conserved sites with ω = 0 and another class of neutral sites with ω = 1. Model M2 (selection) adds a third class of sites with ω >1. Model M3 (discrete) assumes a general discrete distribution. Model M7 (beta) assumes a beta distribution of ω, limited in the range (0, 1), and model M8 (beta & ω) adds an extra site class with ω >1. Models M0, M1 and M7 were set as the null models to be compared against their alternatives (Yang, 2006). The likelihood ratios of these models were compared using the chi-square test. The Kappa values (transition/transversion ratios) were calculated automatically. The results of Bayes Empirical Bayes (BEB) analysis were used in this study [23], except for model M3 for which only the results of Naive Empirical Bayes analysis were available.

## Results

### Phylogenetic analysis

**Figure 1:**
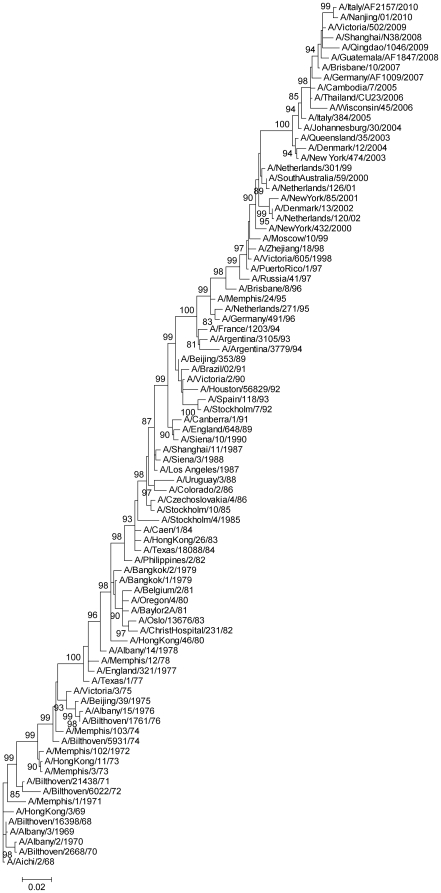
Phylogenetic tree of 86 H3N2 human influenza viruses based on the whole-length sequences of the viral HA1 gene. The tree was calculated using the maximum likelihood method, and bootstrap percentage values out of 1000 replicates are shown at the relevant nodes.

Phylogenetic relationships among the sequences of the seven data sets, as shown in Fig. 1 using data set 3 as an example, were consistent with previous reports [6], [7], demonstrating that the viral gene evolved into few lineages along its stepwise evolutionary history. Most of the bootstrap values of the trees were high, which suggested that the calculated phylogenetic relationships were reliable.

### Estimates of some parameters for the data sets

ML estimates of some parameters of the seven data sets under models M0, M1, M2, M3, M7 and M8 are provided in Table S1. The likelihood ratio tests suggested that model M1 was more suitable than model M0 (*P*<0.01) for all the seven data sets, and model M2 was more suitable than models M1 for all the seven data sets (*P*<0.01 except for data sets 5 and 6), and model M8 was more suitable than models M7 for all the seven data sets (*P*<0.01 except for data sets 5 and 6). Models M2, M3 and M8 showed no significant differences by the likelihood ratio tests, except that models M3 and M8 were more suitable than model M2 for data set 3.

The kappa values varied from 3.94 to 4.85. The ω ratios for the whole gene varied from 0.34 to 0.42 under models M2, M3 and M8 based on different data sets, indicating that the viral gene, in general, is under negative selection. As models M2, M3 and M8 all revealed some sites of the ω ratio >1, this analysis provided significant evidence for positive selection on the viral gene, consistent with some previous reports [6], [7], [10], [12], [16], [17], [24].

### Variation of the sites of the ω ratio >1 calculated from different data sets and models

Sites of the ω ratio >1 calculated under models M2, M3 and M8 from the seven data sets were provided in Table 1. Five to 31 sites of the ω ratio >1 were identified from at least one of the data sets under models M2, M3 or M8. For the same data set, sites of the ω ratio >1 were of little variation among the models of M2, M3 and M8.

**Table 1:** Sites under positive selection calculated using models M2, M3 and M8 from seven data sets.

Data sets	Sequence background	Sites under positive selection		
		Calculated by model M2	Calculated by model M3	Calculated by model M8
1	One sequence for each of the 43 years from 1968–2010	124, 137, 144, 145, 155, 159, 189, 193, 226	124, 137, 144, 145, 155, 159, 189, 193, 226	121, 124, 135, 137, 144, 145, 155, 159, 172, 189, 193, 226
2	Another sequence for each of the 43 years from 1968–2010	2, 3, 50, 124, 133, 137, 144, 145, 155–159, 186, 189, 193, 196, 226	**2** a, **3**, **50**, 82, 83, 121, **124**, **133**, 135, **137**, 138, **144**, **145**, **155**–**158**, 159, 172, 173, **186**, 188, **189**, **193**, **196**, 197, 213, 219, **226**, 273, 278	2, 3, 50, 82, 83, 121, 124, 133, 135, 137, 138, **144**, 145, 155–159, 172, 173, 186, 189, **193**, 196, **226**, 278
3	Data set 1+data set 2	137, 138, 144, **145**, 155, 157, 159, 189, **193**, **226**	50, 121, 124, **137**, 138, **144**, **145**, 155, 156, 157, 159, 172, 186, **189**, **193**, 196, 219, **226**	50, 121, 124, 137, 138, 144, **145**, 155, 156, 157, 159, 186, 189, **193**, 219, **226**
4	The part of data set 1 covering the years 1983–1997	121, 124, 133, 135, 138, 145, 159, 193, 226	**121**, **124**, **133**, **135**, **138**, **145**, 156, **159**, 172, 189, **193**, 196, **226**, 262, 276	121, **124**, 133, **135**, 138, 145, **159**, 172, 193, 196, **226**
5	The part of data set 2 covering the years 1983–1997	94, 121, 124, 133, 135, 156, 159, 186, 196, 219, 226, 262	3, 67, 82, 88, 91, 94, **121**, **124**, **133**, **135**, 137, 145, 155, **156**, 157, **159**, 163, 172, **186**, 189, 193, **196**, 219, **226**, 246, 261, **262**, 276, 278, 279, 299	3, 67, 82, 88, 91, 94, 121, 124, 133, 135, 145, 156, 159, 172, 186, 189, 193, 196, 219, 226, 246, 262, 276, 278, 299
6	The part of data set 3 covering the years 1996–2010	50, 144, 157, 189, 226	50, 144, 157, 189, 226	45, 50, 82, 137, 144, 155, 157, 173, 189, 220, 226
7	The part of data set 3 covering the years 1983–1997	121, 124, 133, 135, 145, 156, 159, 186, 193, 219, 226	94, **121**, **124**, 133, **135**, 138, **145**, 156, **159**, **186**, **193**, 196, 219, **226**, 246, 262	121, 124, 133, 135, 138, **145**, 156, **159**, 186, 193, 196, 219, **226**, 246, 262

a Sites under positive selection with *P*>95% are indicated in bold type.

Table 1 shows that the sites of the ω ratio >1 were of significant variation among the data sets, even though the data sets targeted similar clusters and were analyzed under the same model. For example, data sets 1–3 all covered the years 1968–2010, and the clusters they covered were similar (Fig. 1), but they revealed nine, eighteen and ten sites of the ω ratio >1 under model M2, respectively. For another example, data sets 4 and 5 both covered the years 1983–1997, and the clusters they covered were similar (Fig. 1), but they revealed nine and twelve sites of the ω ratio >1 under model M2, respectively, and shared only five sites of the ω ratio >1 (sites 121, 124, 133, 135 and 226). In total, 45 sites were identified as the sites of the ω ratio >1 by at least one data set, and only site 226 was shared by the seven data sets under any of the three models.

To investigate why the results are of significant differences among the data sets, the nucleotide and amino acid substitutions at fourteen sites (sites 2, 3, 50, 82, 83, 133, 138, 156, 157, 158, 173, 186, 196, 278) within data set 1 and data set 2 were analyzed (Table S2). These sites were identified as the ones of the ω ratio >1 at least by one model using data set 2, but they were identified as the ones of the ω ratio <1 by all the three models using data set 1. Table S2 demonstrates that synonymous substitutions on all these fourteen sites within data set 1 were more or no less than their counterparts within data set 2, while the nonsynonymous substitutions on most (12/14) of these sites within data set 1 were less than their counterparts within data set 2. Therefore, Table S2 indicates that it is likely rational for these fourteen sites to be identified as the ones of the ω ratio >1 using data set 2, but as the ones of the ω ratio <1 using data set 1.

### Specificity and sensitivity of the identification of sites under positive selection

Sites under positive selection in different clusters of a gene may be different [3], [4], [26]–[30], and this may cause some differences of the sites of the ω ratio >1 among the data sets, e.g., some differences between data set 6 and data set 7 which covered different clusters (Fig. 1). However, this explanation can not explain all the differences shown in Table 1, because significant differences among the data sets existed even though they targeted similar clusters as mentioned above. Therefore, the significant variation of the sites of the ω ratio >1 calculated from different data sets suggests that identification of sites under positive selection using the ω ratio is likely to be either of low specificity or of low sensitivity.

Among the 329 sites in the HA1 protein of H3N2 subtype human influenza virus, 131 sites (Table 2) are involved in the BCEs as revealed by the structure of the protein of an influenza virus isolated in 1968 [25]. An additional site, site 220 which lies within 4 Å of BCE D, has also been thought to be involved in the BCEs [16]. The other 197 sites are assumed to be located elsewhere in the protein. Some of the 132 sites involved in the BCEs are thought to be under positive selection pressure in order to escape host antibody immunity, but it is unclear which sites are under positive selection. It is thus impossible to calculate exactly the specificity and the sensitivity of the analysis identifying sites under positive selection using the ω ratio.

**Table 2:** Distribution of the sites under positive selection in the HA1 gene of H3N2 influenza virus calculated by different analyses.

Analyses	Sites under positive selection	
	At BCEs^a^	Elsewhere
By combining the results of seven data sets using the ω ratio (this study)	45, 50, 67, 82, 83, 88, 91, 94, 121, 124, 133, 135, 137, 138, 144, 145, 155–159, 163, 172, 173, 186, 188, 189, 193, 194, 196, 197, 213, 219, 220, 226, 246, 261, 262, 273, 276–279, 299	2, 3
By Shih et al. not using the ω ratio [17]	50, 53, 54, 57, 62, 63, 67, 75, 78, 82, 83, 94, 121, 122, 124, 126, 131, 133, 135, 137, 142, 143, 144, 145, 146, 155, 156, 157, 158, 159, 160, 163, 172, 173, 186, 188, 189, 190, 192, 193, 196, 197, 207, 213, 217, 226, 227, 242, 244, 248, 260, 262, 275, 276, 278, 299, 307	2, 3, 25, 202, 222, 225
By Suzuki et al. using the ω ratio [8]	138, 196, 226	
By Suzuki using the ω ratio [24]	220, 229	
By Bush et al. using the ω ratio [6]	121, 124, 128, 133, 135, 137, 138, 142, 145, 156, 158, 159, 182, 186, 190, 193, 194, 196, 197, 201, 219, 220, 226, 246, 262, 275, 276, 310, 312,	
By Fitch et al. using the ω ratio [7]	121, 133, 135, 137, 138, 145, 156, 186, 190, 193, 194, 196, 226, 276	1, 2, 3,
By Yang et al. using the ω ratio [12]	133, 135, 137, 138, 145, 156, 157, 159, 186, 193, 219, 226	
By Plotkin et al. not using the ω ratio [16]	62, 83, 121, 124, 131, 133, 135, 142, 144, 145, 156, 157, 158, 172, 189, 190, 193, 196, 197, 220, 226, 262, 275, 276, 278, 299	2, 3, 5, 31, 112, 271

a Sites 44–48, 50, 51, 53, 54, 57, 59, 62, 63, 67, 75, 78, 80–83, 86–88, 91, 92, 94, 96, 102, 103, 109, 117, 121, 122, 124, 126, 128–135, 137, 138, 140, 142–146, 150, 152, 155–160, 163–165, 167, 168, 170–177, 179, 182, 186–190, 192–194, 196–198, 201, 203, 207–209, 212–219, 220, 226–230, 238, 240, 242, 244, 246–248, 260, 261, 262, 265, 273, 275, 276, 278–280, 294, 297, 299, 300, 304, 305, 307–312 are considered to be at BCEs, and the other 197 sites in the gene are considered to be out of BCEs from the structure of the gene of a virus isolated in 1968 [16], [25].

Among the 45 sites of the ω ratio >1 identified by at least one model of a data set, 43 (95.56%) are involved in the BCEs which cover less than a half sites in the gene, and the other two sites (site 2 and site 3) lie elsewhere. Therefore, the 43 sites of the ω ratio >1 involved in the BCEs are of high possibility to be really under positive selection. These data suggested that the specificity of the analysis is likely no lower than 98.98% ((197–2)/197) in this case. Because the analysis is likely to be of high specificity, the significant variation of the sites under positive selection calculated from different data sets suggested that the analysis is likely of low sensitivity.

Table 1 also demonstrates that the sensitivity of the analysis can not be enhanced by covering longer time intervals or more sequences. For example, both data sets 5 and 7 covered a shorter time interval and fewer sequences than data set 3, but the positively selected sites identified from data set 3 are fewer than those identified from data sets 5 and 7.

### Comparison with previous reports

Table 2 shows the sites under positive selection in the same viral gene identified using various analyses by previous reports [6]–[8], [12], [16], [17], [24]. As compared with the results of this study, this table demonstrates some interesting data. Firstly, Yang (2000) analyzed 349 sequences of the gene using the ω ratio also identified only 12 positively selected sites [12], also suggesting that the sensitivity of the analysis can not be enhanced by covering more sequences. Secondly, Shih et al. identified 57 sites likely to be under positive selection in the same viral gene using frequency diagrams of amino acid residues rather than the ω ratio in 2007 [17]. They analyzed a total of 2,248 sequences of the gene, and found 63 sites with very rapid amino acid substitutions. They assumed that 57 of the 63 sites are likely to be under positive selection because these 57 sites are involved in the BCEs. Their findings are consistent with the conclusion of this study that at least 43 sites in the gene are likely to be under positive selection. In addition, 32 sites are shared by the 57 sites identified by Shih et al. in 2007 and the 43 sites identified in this study, as shown in Table 2.

Table 2 also shows that Suzuki and Gojobori identified only three sites (sites 138, 196 and 226) in the gene under positive selection in 1999 using the ω ratio [8], and Suzuki identified only two sites (sites 220 and 229) in the gene under positive selection in 2006 using the ω ratio [24]. Bush, Fitch, Yang et al. identified more but no more than 29 sites under positive selection in the gene using the ω ratio [6], [7], [12]. The significant variation of the results of these reports should result from multiple aspects including that the models they used and the sequences or clusters they analyzed were somehow different. Nevertheless, as consistent with this study, these reports all likely identified only a portion of the sites under positive selection in the gene, and almost all the positively selected sites revealed by these reports are involved in the BCEs (Table 2).

## Discussion

The possible low sensitivity of the analysis identifying sites under positive selection may be explained by several reasons. Firstly, if a site is under positive selection, but no mutations occurred during the observation period, then, using this method, the site would be assumed not to be under positive selection [1]. This is likely to be the case if the compared sequences are from genetically close clusters. This bias can be minimized by selecting sequences over a longer time interval. However, this bias can not explain all of the scenarios described in Table 1 because, as mentioned above, the sensitivity of the analysis can not be enhanced by covering longer time intervals or more sequences.

Previously, sites in a gene were assumed to be under neutral selection, negative selection or positive selection without overlap. In fact, some sites in a gene under positive selection for fitness may be also under negative selection for functional constrains simultaneously. Theoretically, the sites under these two opposite selection forces simultaneously favor nonsynonymous changes, but they favor only certain nonsynonymous changes, e.g. those changing the antigenicity of the gene and maintaining the gene function. It has been found that several epitopic residues within the HA1 gene of human H3N2 subtype influenza virus exhibited mainly synonymous variation for strong functional constrains [16], and these epitopic residues can be taken as the extreme examples of the claim that some sites under positive selection are also under negative selection at the same time for functional constraints. Furthermore, it has been widely accepted that a site in a gene may be positively selected in one cluster, but negatively selected in another cluster [3], [4], [26]–[30]. This fact actually indicates that the site, which usually plays the same functional role in the gene among different clusters, is likely to be under positive selection pressure and negative selection pressure at the same time, and it exhibits different selection effect in different clusters just because of some random or non-random factors [26]–[29]. Due to that a site under positive selection for fitness may be also under negative selection simultaneously for function constrains, the ω ratio of a site under positive selection may be <1, and so the sensitivity of the analysis identifying sites under positive selection using the ω ratio from one data set is low, and the sensitivity can not be enhanced by including more sequences or covering longer time intervals. This is similar to that it is of low sensitivity to identify an object being pushed through searching the objects moving forward because an object being pushed may keep still or move back for co-existence of friction or other kinds of contrary forces.

The above view does not deny the important use of the ω ratio in analyzing molecular selection. It is just like that sometimes we had better analyze the dynamic of a moving object not from the resultant force, but from two opposite forces, e.g. a driving force and friction which are really co-existing. This view is helpful for us to understand why only a few sites in the HA1 gene of influenza viruses were found to be under positive selection in some previous reports [6]–[8], [24], [26], [31], and why in this study the positively selected sites estimated from different data sets are of significant difference even though the data sets targeted similar clusters. Furthermore, this study also suggests that sometimes we can enhance the sensitivity of the analysis identifying sites under positive selection using the ω ratio by integrating all the sites of the ω ratio >1 estimated from different data sets of the same gene. For example, if we integrate all the sites of the ω ratio >1 estimated in this study from each of the seven data sets, we will find that at least 45 sites are likely to be under positive selection.

Due to that some sites in a gene under positive selection may not exhibit the effect of positive selection from the ω ratio, we may had better differentiate in the future a positively selected site from a site under positive selection. A positively selected site can be defined as the one of the ω ratio >1, which means that the site has exhibited the effect of positive selection, while a site under positive selection may have not exhibited the effect of positive selection, though it is under positive selection, namely that the ω ratio of a site under positive selection may be >1,  = 1, or <1. This differentiation is helpful for us to understand the intricate nature of molecular evolution, like that we should differentiate an object moving forward from an object being pushed, as the later may keep still or move back for co-existence of friction or other kinds of contrary forces.

The method used in this study to estimate the ω ratio of each site in a gene from one data set has been well established, and is likely able to identify all the sites of the ω ratio >1 [9]–[16], [28], [32], which is also consistent with Table S2. However, this method may be of low sensitivity to identify the sites under positive selection from just one data set for the reason given above. In other words, according to the aforementioned differentiation, analysis of the ω ratio of each site in a gene from just one data set is likely able to identify some positively selected sites rather than all the sites under positive selection within the relevant cluster.

As shown in Table 2, this study and some previous reports including references 7, 16 and 17 all indicate that sites 2 and 3, if they are not false positives of the analyses, should be also under positive selection. These two residues are on the surface of the HA trimer, but they lie far away from the BCEs [16], [25]. It remains unclear why these two residues may be under positive selection.

## Supporting Information

Table S1 Estimates of some parameters of data sets 1–7.
(DOC)

Table S2 The codons and amino acid residues (in parentheses) of fourteen sites within data set 1 and data set 2.
(XLS)

Text S1 The 86 sequences analyzed in this study.
(DOC)
