# Supplementary material for: Variation in the Analysis of Positively Selected Sites Using Nonsynonymous/Synonymous Rate Ratios: An Example Using Influenza Virus
Source: PLoS One. 2011 May 24;6(5):e19996. doi: 10.1371/journal.pone.0019996 (PMC3101217; doi:10.1371/journal.pone.0019996)
Supplement: Table S1 — Estimates of some parameters of data sets 1–7. (DOC) [file pone.0019996.s001.doc]

Supporting Information

**Table S1.** Estimates of some parameters of data sets 1-7

|  | Model | L | np | Parameters | *d*_N_/*d*_S_ | Kappa |
| --- | --- | --- | --- | --- | --- | --- |
| Data set 1 | 0 (one ratio) | -4260.4622 | 85 | ω = 0.34355 | 0.34355 | 4.60177 |
|  | 1 (neutral) | -4210.9006 | 86 | *p_0_* =0.75698, ω*_0_* = 0.0981 | 0.3173 | 4.56830 |
|  |  |  |  | *p_1_* =0.24302, ω*_1_* = 1.00000 |  |  |
|  | 2 (selection) | -4208.8455 | 88 | *p_0_* = 0.76559, ω*_0_* = 0.10876 | 0.3515 | 4.70217 |
|  |  |  |  | *p_1_* = 0.21374, ω*_1_* = 1.00000 |  |  |
|  |  |  |  | *p_2_* = 0.02067, ω*_2_* = 2.63790 |  |  |
|  | 3 (discrete) | -4208.3707 | 103 | *p_0-3_* = 0.65609, ω*_0-3_* ≈ 0.07160 | 0.3435 | 4.68234 |
|  |  |  |  | *p_4-7_* = 0.29500, ω*_4-7_* ≈ 0.65568 |  |  |
|  |  |  |  | *p_8-9_* = 0.04891, ω*_8-9_* ≈ 2.10870 |  |  |
|  | 7 (beta) | -4213.2089 | 86 | *p* = 0.20760, *q* = 0.45443 | 0.3136 | 4.56644 |
|  | 8(beta & ω) | -4208.4383 | 88 | *p_0_* = 0.94830 | 0.3432 | 4.68021 |
|  |  |  |  | *p* = 0.38788, *q* = 1.15400 |  |  |
|  |  |  |  | *p*_1_ = 0.05170, ω = 2.05276 |  |  |
| Data set 2 | 0 (one ratio) | -4442.2544 | 85 | ω = 0.38353 | 0.38353 | 4.75895 |
|  | 1 (neutral) | -4367.5939 | 86 | *p_0_* = 0.74139, ω*_0_* = 0.07812 | 0.3165 | 4.55850 |
|  |  |  |  | *p_1_* =0.25861, ω*_1_* = 1.00000 |  |  |
|  | 2 (selection) | -4362.1945 | 88 | *p_0_* =0.76988, ω*_0_* = 0.10284 | 0.3848 | 4.80236 |
|  |  |  |  | *p_1_* = 0.16797, ω*_1_* = 1.00000 |  |  |
|  |  |  |  | *p_2_* = 0.06216, ω*_2_* = 2.21416 |  |  |
|  | 3 (discrete) | -4360.7786 | 103 | *p_0_* =0.40512, ω*_0_* = 0.00000 | 0.3785 | 4.78417 |
|  |  |  |  | *p_1-6_* = 0.47433, ω*_1-6_* ≈ 0.32552 |  |  |
|  |  |  |  | *p_7-9_* = 0.12055, ω*_7-9_* ≈ 1.85890 |  |  |
|  | 7 (beta) | -4371.0584 | 86 | *p* =0.13565, *q* = 0.28615 | 0.3216 | 4.55154 |
|  | 8(beta & ω) | -4361.1275 | 88 | *p_0_* = 0.89169 | 0.3843 | 4.85008 |
|  |  |  |  | *p* = 0.47099, *q* = 1.94251 |  |  |
|  |  |  |  | *p*_1_ = 0.10831, ω = 1.91799 |  |  |
| Data set 3 | 0 (one ratio) | -5792.9461 | 171 | ω = 0.35984 | 0.35984 | 4.74031 |
|  | 1 (neutral) | -5702.7363 | 172 | *p_0_* = 0.76616, ω*_0_* = 0.07812 | 0.3165 | 4.62357 |
|  |  |  |  | *p_1_* = 0.23384, ω*_1_* = 1.00000 |  |  |
|  | 2 (selection) | -5696.4009 | 174 | *p_0_* = 0.77052, ω*_0_* = 0.10284 | 0.3674 | 4.80965 |
|  |  |  |  | *p_1_* = 0.20002, ω*_1_* = 1.00000 |  |  |
|  |  |  |  | *p_2_* = 0.11945, ω*_2_* = 2.55652 |  |  |
|  | 3 (discrete) | -5694.1684 | 189 | *p_0-2_* =0.22037, ω*_0_* ≈ 0.00000 | 0.3545 | 4.75471 |
|  |  |  |  | *p_3-4_* = 0.40105, ω*_1-6_* ≈ 0.11665 |  |  |
|  |  |  |  | *p_5_* =0.22037, ω*_5_* =0.54338 |  |  |
|  |  |  |  | *p_6-9_* = 0.07030, ω*_6-9_* ≈ 1.99400 |  |  |
|  | 7 (beta) | -5706.7488 | 172 | *p* = 0.24178, *q* = 0.53589 | 0.3136 | 4.59197 |
|  | 8(beta & ω) | -5694.1933 | 174 | *p_0_* = 0.93634 | 0.3545 | 4.75731 |
|  |  |  |  | *p* = 0.45954, *q* = 1.44287 |  |  |
|  |  |  |  | *p*_1_ = 0.06366, ω = 2.04734 |  |  |
| Data set 4 | 0 (one ratio) | -2373.5872 | 29 | ω = 0.34214 | 0.34214 | 3.87873 |
|  | 1 (neutral) | -2350.2345 | 30 | *p_0_* = 0.71783, ω*_0_* = 0.00525 | 0.2859 | 3.76708 |
|  |  |  |  | *p_1_* = 0.28217, ω*_1_* = 1.00000 |  |  |
|  | 2 (selection) | -2346.3717 | 32 | *p_0_* = 0.87876, ω*_0_* = 0.10993 | 0.3548 | 3.94443 |
|  |  |  |  | *p_1_* = 0.04182, ω*_1_* = 1.00000 |  |  |
|  |  |  |  | *p_2_* = 0.07942, ω*_2_* = 2.72504 |  |  |
|  | 3 (discrete) | -2346.3641 | 47 | *p_0_* =0.40512, ω*_0_* = 0.00000 | 0.3549 | 3.94694 |
|  |  |  |  | *p_1-6_* = 0.47433, ω*_1-6_* ≈ 0.32552 |  |  |
|  |  |  |  | *p_7-9_* = 0.12055, ω*_7-9_* ≈ 1.85890 |  |  |
|  | 7 (beta) | -2350.2760 | 30 | *p* = 0.00500, *q* = 0.01156 | 0.3000 | 3.79881 |
|  | 8(beta & ω) | -2346.3657 | 32 | *p_0_* = 0.91582 | 0.3549 | 3.94633 |
|  |  |  |  | *p* = 0.84418, *q* = 5.07279 |  |  |
|  |  |  |  | *p*_1_ = 0.08418, ω = 2.69562 |  |  |
| Data set 5 | 0 (one ratio) | -2545.6389 | 29 | ω = 0.34801 | 0.34801 | 4.58869 |
|  | 1 (neutral) | -2519.0134 | 30 | *p_0_* = 0.69149, ω*_0_* = 0.08342 | 0.3085 | 4.44856 |
|  |  |  |  | *p_1_* = 0.30851, ω*_1_* = 1.00000 |  |  |
|  | 2 (selection) | -2517.5175 | 32 | *p_0_* = 0.82583, ω*_0_* = 0.07495 | 0.3523 | 4.60711 |
|  |  |  |  | *p_1_* = 0.00000, ω*_1_* = 1.00000 |  |  |
|  |  |  |  | *p_2_* = 0.17417, ω*_2_* = 1.66751 |  |  |
|  | 3 (discrete) | -2517.4927 | 47 | *p_0-2_* = 0.51318, ω*_0_* ≈ 0.00000 | 0.3528 | 4.60618 |
|  |  |  |  | *p_3-4_* = 0.33892, ω*_3-4_* ≈ 0.11665 |  |  |
|  |  |  |  | *p_5_* = 0.00002, ω*_5_* = 0.40972 |  |  |
|  |  |  |  | *p_6-8_* = 0.14778, ω*_6-8_* ≈1.78826 |  |  |
|  |  |  |  | *p_9_* = 0.00009, ω*_9_* = 1.79122 |  |  |
|  | 7 (beta) | -2519.0248 | 30 | *p* = 0.00533, *q* = 0.01047 | 0.3026 | 4.43454 |
|  | 8(beta & ω) | -2517.5012 | 32 | *p_0_* = 0.84737 | 0.3527 | 4.60642 |
|  |  |  |  | *p* = 0.41609, *q* = 3.63467 |  |  |
|  |  |  |  | *p*_1_ = 0.15263, ω = 1.76089 |  |  |
| Data set 6 | 0 (one ratio) | -2772.1897 | 59 | ω = 0.40895 | 0.40895 | 4.17004 |
|  | 1 (neutral) | -2754.7042 | 60 | *p_0_* = 0.64778, ω*_0_* = 0.08342 | 0.3812 | 4.14197 |
|  |  |  |  | *p_1_* = 0.04470, ω*_1_* = 1.00000 |  |  |
|  | 2 (selection) | -2753.5743 | 62 | *p_0_* = 0.68582, ω*_0_* = 0.07959 | 0.4196 | 4.22322 |
|  |  |  |  | *p_1_* = 0.28890, ω*_1_* = 1.00000 |  |  |
|  |  |  |  | *p_2_* = 0.02528, ω*_2_* = 3.00923 |  |  |
|  | 3 (discrete) | -2753.1696 | 77 | *P_0-1_* =0.50378, ω*_0_* = 0.00000 | 0.4106 | 4.19749 |
|  |  |  |  | *p_2-6_* = 0.45699, ω*_2-6_* ≈ 0.65795 |  |  |
|  |  |  |  | *p_7-9_* = 0.08811, ω*_7-9_* ≈ 2.80177 |  |  |
|  | 7 (beta) | -2754.8588 | 60 | *p* = 0.01461, *q* = 0.01927 | 0.4089 | 4.20986 |
|  | 8(beta & ω) | -2753.3518 | 62 | *P_0_* = 0.96704 | 0.4133 | 4.20576 |
|  |  |  |  | *p* = 0.19562, *q* = 0.39755 |  |  |
|  |  |  |  | *p*_1_ = 0.03296, ω = 2.86271 |  |  |
| Data set 7 | 0 (one ratio) | -3116.4360 | 59 | ω = 0.35006 | 0.35006 | 4.16538 |
|  | 1 (neutral) | -3076.9951 | 60 | *P_0_* = 0.75413, ω*_0_* = 0.05538 | 0.2876 | 3.98167 |
|  |  |  |  | *P_1_* = 0.24587, ω*_1_* = 1.00000 |  |  |
|  | 2 (selection) | -3072.8765 | 62 | *P_0_* = 0.81076, ω*_0_* = 0.09367 | 0.3510 | 4.17430 |
|  |  |  |  | *p_1_* = 0.13064, ω*_1_* = 1.00000 |  |  |
|  |  |  |  | *p_2_* = 0.05860, ω*_2_* = 2.46369 |  |  |
|  | 3 (discrete) | -3072.6081 | 77 | *p_0-1_* =0.46905, ω*_0_* = 0.00000 | 0.3492 | 4.17694 |
|  |  |  |  | *p_1-6_* = 0.45699, ω*_1-6_* ≈ 0.34558 |  |  |
|  |  |  |  | *p_7-9_* = 0.03923, ω*_7-9_* ≈ 2.22596 |  |  |
|  | 7 (beta) | -3078.0256 | 60 | *p* = 0.01187, *q* = 0.02307 | 0.3206 | 4.08437 |
|  | 8(beta & ω) | -3072.6873 | 62 | *P_0_* = 0.91662 | 0.3493 | 4.17465 |
|  |  |  |  | *p* = 0.40701, *q* = 1.86325 |  |  |
|  |  |  |  | *p*_1_ = 0.08338, ω = 2.25296 |  |  |
