# Supplementary material for: Variation in the Analysis of Positively Selected Sites Using Nonsynonymous/Synonymous Rate Ratios: An Example Using Influenza Virus
Source: PLoS One. 2011 May 24;6(5):e19996. doi: 10.1371/journal.pone.0019996 (PMC3101217; doi:10.1371/journal.pone.0019996)
Supplement: Text S1 — The 86 sequences analyzed in this study. (DOC) [file pone.0019996.s003.doc]

Supporting Information

Text S1 The 86 sequences analyzed in the report

(Note: the 43 sequences are those of data set 1 followed by the other 43 sequences of data set 2)

A/Bilthoven/16398/68

CAAGACCTTCCAGGAAATGACAACAGCACAGCAACGCTGTGCCTGGGACATCATGCGGTGCCAAACGGAACACTAGTGAAAACAATCACAGATGATCAGATTGAAGTGACTAATGCTACTGAGCTAGTTCAGAGCTCCTCAACGGGGAAAATATGCAACAATCCTCATCGAATCCTTGATGGAATAAACTGCACACTGATAGATGCTCTATTGGGGGACCCTCATTGTGATGTTTTTCAAGATGAGACATGGGACCTTTTCGTTGAACGCAGCAAAGCTTTCAGCAACTGTTACCCTTATGATGTGCCAGATTATGCCTCCCTTAGGTCACTAGTTGCCTCGTCAGGCACTCTGGAGTTTATCACTGAGGGTTTCACTTGGACTGGGGTCACTCAGAATGGGGGAAGCAATGCTTGCAAAAGGGGACCTGGTAGCGGTTTTTTCAGTAGACTGAACTGGTTGACCAAATCAGGAAGCACATATCCAGTGCTGAACGTGACTATGCCAAACAATGACAATTTTGACAAACTATACATTTGGGGGGTTCACCACCCGAGCACGAACCAAGAACAAACCAGCCTGTATGTTCAAGCATCAGGGAGAGTCACAGTCTCTACCAGGAGAAGCCAGCAAACTATAATCCCGAATATCGGGTCCAGACCCTGGGTAAGGGGTCTGTCTAGTAGAATAAGCATCTATTGGACAATAGTTAAGCCGGGAGACGTACTGGTAATTAATAGTAATGGGAACCTAATCGCTCCTCGGGGTTATTTCAAAATGCGCACTGGGAAAAGCTCAATAATGAGGTCAGATGCACCTATTGATACCTGTATTTCTGAATGCATCACTCCAAATGGAAGCATTCCCAATGACAAGCCCTTTCAAAACGTAAACAAGATCACATATGGAGCATGCCCCAAGTATGTTAAGCAAAACACCCTGAAGTTGGCAACAGGGATGCGGAATGTACCAGAGAAACAAACTAGA

A/Albany/3/1969

CAAGACCTTCCAGGAAATGACAACAGCACAGCAACGCTGTGCCTGGGACATCATGCGGTGCCAAACGGAACACTAGTGAAAACAATCACAGATGATCAGATTGAAGTGACTAATGCTACTGAGCTAGTTCAGAGCTCCTCAACGGGGAAAATATGCAACAATCCTCATCGAATCCTTGATGGAATAAACTGCACACTGATAGATGCTCTATTGGGGGACCCTCATTGTGATGTTTTTCAAGATGAGACATGGGACCTTTTCGTTGAACGCAGCAAAGCTTTCAGCAACTGTTACCCTTATGATGTGCCAGATTATGCCTCCCTTAGGTCACTAGTTGCCTCGTCAGGCACTCTGGAGTTTATCACTGAGGGTTTCACTTGGACTGGGGTCACTCAGAATGGGGGAAGCAATGCTTGCAAAAGGGGACCTGGTAGCGGTTTTTTCAGTAGACTGAACTGGTTGACCAAATCAGGAAGCACATATCCAGTGCTGAACGTGACTATGCCAAACAATGACAATTTTGACAAACTATACATTTGGGGGGTTCACCACCCGAGCACGAACCAAGAACAAACCAGCCTGTATGTTCAAGCATCAGGGAGAGTCACAGTCTCTACCAGGAGAAGCCAGCAAACTATAATCCCGAATATCGGGTCCAGACCCTGGGTAAGGGGTCTGTCTAGTAGAATAAGCATCTATTGGACAATAGTTAAGCCGGGAGACGTACTGGTAATTAATAGTAATGGGAACCTAATCGCTCCTCGGGGTTATTTCAAAATGCGCACTGGGAAAAGCTCAATAATGAGGTCAGATGCATCTATTGATACCTGTATTTCTGAATGCATCACTCCAAATGGAAGCATTCCCAATGACAAGCCCTTTCAAAACGTAAACAAGATCACATATGGAGCATGCCCCAAGTATGTTAAGCAAAACACCCTGAAGTTGGCAACAGGGATGCGGAATGTACCAGAGAAACAAACTAGA

A/Albany/2/1970

CAAGACCTTCCAGGAAATGACAACAGTACAGCAACGCTGTGCCTGGGACATCATGCGGTGCCAAACGGAACACTAGTGAAAACAATCACAAATGATCAGATTGAAGTGACTAATGCTACTGAGCTAGTTCAGAGTTCCTCAACGGGGAAAATATGCAACAATCCTCATCGAATCCTTGATGGAATAAACTGCACACTGATAGACGCTCTATTGGGGGACCCTCATTGTGATGTTTTCCAAGATGAGACATGGGACCTTTTCGTTGAACGCAGCAAAGCTTTCAGCAACTGTTACCCTTATGATGTGCCAGATTATGCCTCCCTTAGGTCACTAGTTGCCTCGTCAGGCACTCTGGAGTTTATCACTGAGGGTTTCACTTGGACTGGGGTCACTCAGAATGGGGGAAGCAATGCTTGCAAAAGGGGACCTGGTAGCGGTTTTTTCAGTAGACTGAACTGGTTGACCAAATCAGGAAGCACATATCCAGTGCTGAACGTGACTATGCCAAACAATGACAATTTTGACAAACTATACATTTGGGGGGTTCACCACCCGAGCACGAACCAAGAACAAACCAGCCTGTATGTTCAAGCATCAGGGAGAGTCACAGTCTCTACCAGGAGAAGCCAGCAAACTATAATCCCGAATATCGGGTCCAGACCCTGGGTAAGGGGTCTGTCTAGTAGAATAAGCATCTATTGGACAATAGTTAAACCGGGAGACGTACTGGTAATTAATAGTAATGGGAACCTAATCGCTCCTCGGGGTTATTTCAAAATGCGCACTGGGAAAAGCTCAATAATGAGGTCAGATGCACCTATTGATACCTGTATTTCTGAATGCATCACTCCAAATGGAAGCATTCCCAATGACAAGCCCTTTCAAAACGTAAACAAGATCACATATGGAGCATGCCCCAAGTATGTTAAGCAAAACACCCTGAAATTGGCAACAGGGATGCGGAATGTACCAGAGAAACAAACTAGA

A/Memphis/1/1971

CAATACCTTCCAGGAAATGACAACAGCACAGCAACGCTGTGTCTGGGACATCATGCAGTGCCAAACGGAACACTAGTGAAAACAATCACAAATGATCAGATTGAAGTGACTAATGCTACTGAGCTAGTTCAGAGCTCCTCAACGGGGAAAATATGCAACAATCCTCATCGAATCCTTGATGGAATAGACTGCACACTGATAGATGCTCTATTGGGGGACCCTCATTGTGATGGTTTTCAAAATGAGACATGGGACCTTTTCGTTGAACGCAGCAAAGCTTTCAGCAACTGTTACCCCTATGATGTGCCAGATTATGCCTCCCTTAGATCACTAGTTGCCTCGTCAGGCACTCTGGAGTTTATCACTGAGGGTTTCACTTGGACTGGGGTCACTCAGAATGGGGGAAGCAATGCTTGCAAAAGGGGACCTGGTAGCGGTTTTTTCAGTAGACTGAACTGGTTGACCAAATCAGAAAGCACATATCCAGTGCTGAACGTGACTATGCCAAACAATGACAATTTTGACAAACTATACATTTGGGGAGTTCACCACCCGAGCACGAACCAAGAACAAACCAGCCTGTATGTTCAAGCATCAGGGAGAGTCACAGTCTCTACCAGGAGAAGCCAGCAAACTATAATCCCGAATATCGGGTCTAGACCCTGGGTAAGGGGTCTGTCTAGTAGAATAAGCATCTATTGGACAATAGTTAAGCCCGGGGACGTACTGGTAATTAATAGTAATGCGAACCTAATCGCTCCTCGGGGTTATTTCAAAATGCGCACTGGGAAAAGCTCAATAATGAGGTCAGATGCACCTATTGATACCTGTATTTCTGAATGCATCACTCCAAATGGAAGCATTCCCAACGACAAGCCCTTTCAAAACGTAAACAAGATCACATATGGAGCATGCCCCAAGTATGTTAAGCAAAACACCCTGAAGTTGGCAACACGGATGCGGAATGTACCAGAGAAACAAACTAGA

A/Memphis/102/1972

CAAGACTTTCCAGGAAATGACAACAGCACAGCAACGCTGTGCCTGGGACATCATGCGGTGCCAAACGGAACACTAGTGAAAACAATCACAAATGATCAGATTGAAGTGACTAATGCTACTGAGCTGGTTCAGAGTTCCTCAACGGGGAAAATATGCAACAATCCTCATCGAATCCTTGATGGAATAGACTGCACACTGATAGATGCTCTATTGGGGGACCCTCATTGTGATGGCTTTCAAAATGAGACATGGGACCTTTTCGTTGAACGCAGCAAAGCTTTCAGCAACTGTTACCCTTATGATGTGCCAGATTATGCCTCCCTTAGGTCACTAGTTGCCTCGTCAGGCACTTTGGAGTTTATCAATGAAGGCTTCACTTGGACTGGGGTCACTCAGAATGGGGGAAGCAATGCTTGCAAAAGGGGACCTGATAGCGGTTTTTTCAGTAGACTGAACTGGTTGTACAAATCAGGAAGCACATATCCAGTGCTGAATGTGACTATGCCAAACAATGACAATTTTGACAAACTATACATTTGGGGGGTTCACCACCCGAGCACGGACCAAGAACAAACCAGCCTATATGTTCAAGCATCAGGGAGAGTCACAGTCTCTACCAAGAGAAGCCAGCAAACTATAATCCCGAATATCGGGTCTAGACCCTGGGTAAGGGGTCTGTCTAGTAGAATAAGCATCTATTGGACAATAGTTAAACCGGGGGACATACTGGTAATTAATAGTAATGGGAACCTAATTGCTCCTCGGGGTTATTTCAAAATGCGCACTGGGAAAAGCTCAATAATGAGGTCAGATGCACCTATTGGCACCTGCATTTCTGAATGCATCACTCCAAATGGAAGCATTCCCAATGACAAGCCCTTTCAAAACGTAAACAAGATCACATATGGGGCATGTCCCAAGTATGTTAAGCAAAACACCCTGAAGTTGGCAACAGGGATGCGGAATGTACCAGAGAAACAAACTAGA

A/HongKong/11/73

CAAGACTTTCCAGGAAATGACAACAGCACAGCAACGCTGTGCCTGGGACATCATGCGGTGCCAAACGGAACACTAGTGAAAACAATCACAAATGATCAGATTGAAGTGACTAATGCTACTGAGCTGGTTCAGAGTTCCTCAACGGGGAAAATATGCAACAATCCTCATCGAATCCTTGATGGAATAGACTGCACACTGATAGATGCTCTATTGGGGGACCCTCATTGTGATGGCTTTCAAAATGAGACATGGGACCTTTTCGTTGAACGCAGCAAAGCTTTCAGCAACTGTTACCCTTATGATGTGCCAGATTATGCCTCCCTTAGGTCACTAGTTGCCTCGTCAGGCACTCTGGAGTTTATCAATGAAGGCTTCACTTGGACTGGGGTCACTCAGAATGGGGGAAGCAATGCTTGCAAAAGGGGACCTGATAGCGGTTTTTTCAGTAGACTGAACTGGTTGTACAAATCAGGAAGCACATATCCAGTGCTGAACGTGACTATGCCAAACAATGACAATTTTGACAAACTATACATTTGGGGGGTTCACCACCCGAGCACGGACCAAGAACAAACCAGCCTATATGTTCAAGCATCAGGGAGAGTCACAGTCTCTACCAAGAGAAGCCAGCAAACTATAATCCCGAATATCGGGTCTAGACCCTGGGTAAGGGGTCTGTCTAGTAGAATAAGCATCTATTGGACAATAGTTAAACCAGGAGACATACTGGTACTTAATAGTAATGGGAACCTAATTGCTCCTCGGGGTTATTTCAAAATGCGCACTGGGAAAAGCTCAATAATGAGGTCAGATGCACCTATTGGCACCTGCATTTCTGAATGCATCACTCCAAATGGAAGCATTCCCAATGACAAGCCCTTTCAAAACGTAAACAAGATCACATATGGGGCATGTCCCAAGTATGTTAAGCAAAACACCCTGAAGTTGGCAACAGGGATGCGGAATGTACCAGAGAAACAAACTAGA

A/Bilthoven/5931/74

CAAGACCTTCCAGGAAATGACAACAGCACAGCAACGCTGTGCCTGGGACATCATGCGGTGCCAAACGGAACGCTAGTGAAAACAATCACGAATGATCAGATTGAAGTGACTAATGCTACTGAGCTGGTTCAGAGTTCCTCAACGGGTAAAATATGCAACAATCCTCATCGAATCCTTGATGGAATAAACTGCACACTGATAGATGCTCTATTGGGGGACCCTCATTGTGATGGCTTTCAAAATGAGAAATGGGACCTTTTTGTTGAACGCAGCAAAGCTTTCAGCAATTGTTACCCTTATGATGTGCCAGATTATGCCTCCCTTAGGTCACTAGTTGCCTCGTCAGGCACTCTGGAGTTTATCAATGAAGGCTTCAATTGGACTGGGGTCACTCAGAATGGGGGAAGCAATGCTTGCAAAAGGGGACCTGACAGCGGTTTTTTCAGTAGACTGAACTGGTTGTACAAATCAGGAAGCACATATCCAGTGCTGAACGTGACTATGCCAAACAATGACAATTTTGACAAACTATACATTTGGGGGGTTCACCACCCGAGCACGGACCAAGAACAAACCAACCTATATGTTCAAGCATCAGGGAGAGTCACAGTCTCCACCAAGAGAAGCCAGCAAACTATAATCCCGAATATCGGGTCTAGACCCTGGGTAAGGGGTCTGTCTAGTAGAATAAGCATCTATTGGACAATAGTTAAACCGGGAGACATACTGGTAATTAATAGTAATGGGAACCTAATTGCTCCTCGGGGTTACTTCAAAATGCGCACTGGGAAAAGCTCAATAATGAGGTCAGATGCACCTATTGGCACCTGCATTTCTGAATGCATCACTCCAAACGGAAGCATTCCCAATGACAAGCCCTTTCAAAACGTAAACAAGATCACATATGGGGCATGTCCCAAGTATGTTAAGCAAAACACTCTGAAGTTGGCAACAGGGATGCGGAATGTACCAGAGAAACAAACTAGA

A/Beijing/39/1975

CAAGACCTTCCAGGAAATGACAACAGCACAGCAACGCTGTGCCTGGGACATCATGCGGTGCCAAACGGAACGCTAGTGAAAACAATCACGAATGATCAGATTGAAGTGACTAATGCTACTGAACTGGTTCAGAGTTCCTCAACGGGTAAAATATGCGACAATCCTCATCGAATCCTTGATGGAATAAACTGCACACTGATAGATGCTCTATTGGGGGACCCTCATTGTGATGGCTTTCAAAATGAGAAATGGGACCTTTTCGTTGAACGCAGCAAAGCTTTCAGCAACTGTTACCCTTATGATGTGCCAGATTATGCCTCCCTTAGGTCACTAGTTGCCTCGTCAGGCACCCTGGAGTTTATCAATGAAGGCTTCAATTGGACTGGGGTCACTCAGAATGGGGGAAGCAGTGCTTGCAAAAGGGGACCTGATAACGGTTTTTTCAGTAGACTGAACTGGTTGTACAAATCAGGAAGCACATATCCAGTGCAGAACGTGACCATGCCAAACAATGACAATTCTGACAAACTATACATTTGGGGGGTTCACCACCCGAACACGGACAAAGAACAAACCGACCTATATGTTCAAGCATCAGGGAAAGTCACAGTCTCCACCAAGAGAAGCCAGCAAACTATAATCCCGAATGTCGGGTCTAGACCCTGGGTAAGGGGTCTGTCTAGTAGAGTAAGCATCTATTGGACAATAGTTAAACCGGGAGACATACTGGTAATTAATAGTAATGGAAACCTAATTGCTCCTCGGGGTTACTTCAAAATGCGCACTGGGAAAAGCTCAATAATGAGGTCAGATGCACCTATTGGCACCTGCAGCTCTGAATGCATCACTCCAAATGGAAGCATTCCCAATGACAAGCCCTTTCAAAACGTAAACAAGATCACATATGGGGCATGTCCCAAGTATGTTAAGCAAAACACTCTGAAGTTGGCAACAGGGATGCGGAATGTACCAGAGAAACAAACTAGA

A/Albany/15/1976

CAAGGCCTTCCAGGAAATGACAACAGCACAGCAACTCTGTGCCTGGGACATCATGCGGTGCCAAACGGAACGCTAGTGAAAACAATCACGAATGATCAGATTGAAGTGACTAATGCTACTGAACTGGTTCAGAGTTCCTCAACGGGTAAAATATGCGACAATCCTCATCGAATCCTTGATGGAATAAACTGCACACTGATAGATGCTCTATTGGGGGACCCTCATTGTGATGGCTTTCAAAATGAGAAATGGGACCTTTTCGTTGAACGCAGCAAAGCTTTCAGCAACTGTTACCCTTATGATGTGCCAGATTATGCCTCCCTTAGGTCACTAGTTGCCTCGTCAGGCACTCTGGAGTTTATCAATGAAGGCTTCAATTGGACTGGGGTCACTCAGAATGGGGGAAGCAGTTCTTGCAAAAGGGGACCTGATAACGGTTTTTTCAGTAGACTGAACTGGTTGTACAAATCAGGAAGCACATATCCAGTGCAGAACGTGACCATGCCAAACAATGACAACTCTGACAAACTATACATTTGGGGGGTTCACCACCCGAGCACGGACAAAGAACAAACCGACCTATATGTTCAAGCATCAGGGAAAGTCACAGTCTCCACCAAGAGAAGCCAGCAAACTGTAATCCCGAATGTCGGGTCTAGACCCTGGGTAAGGGGTCTGTCTAGTAGAGTAAGCATCTATTGGACAATAGTTAAACCGGGAGACATACTGGTAATTAATAGTAATGGAAACCTAATTGCTCCTCGGGGTTACTTCAAAATGCGCACTGGGAAAAGCTCAATAATGAGGTCAGATGCACCTATTGGCACCTGCAGCTCTGAATGCATCACTCCAAATGGAAGCATTCCCAATGACAAGCCCTTTCAAAACGTAAACAAGATCACATATGGGGCATGTCCCAAGTATGTTAAGCAAAACACTCTGAAGTTGGCAACAGGGATGCGGAATGTACCAGAGAAACAAACTAGA

A/England/321/1977

CAAAACCTTCCAGGAAATGACAACAGCACAGCAACGCTGTGCCTGGCACATCATGCAGTGCCAAACGGAACGCTAGTGAAAACAATCACGAATGATCAGATTGAAGTGACTAATGCTACTGAGCTGGTTCAGAGTTCCTCAACAGGTAGAATATGCGACAGTCCTCATCGAATCCTTGATGGAAAAAACTGCACACTGATAGATGCTCTATTGGGGGACCCTCATTGTGATGGCTTTCAAAATGAGAAATGGGACCTTTTTGTTGAACGCAGCAAAGCTTTCAGCAACTGTTACCCTTATGATGTGCCAGATTATGCCTCCCTTAGGTCACTAGTTGCCTCGTCAGGCACCCTGGAGTTTATCAATGAAGGCTTCAATTGGACTGGGGTCACTCAGAATGGGGGAAGCTATGCTTGCAAAAGGGGACCTGATAACAGTTTCTTCAGTAGACTGAACTGGTTGTACAAATCAGAAAGCACATATCCAGTGCTGAACGTGACTATGCCAAACAATGACAATTTTGACAAACTGTACATTTGGGGAGTTCACCACCCGAGCACGGACAAAGAACAAACCAAACTATATGTTCAAGCATCAGGGAGAGTCACAGTCTCCACCAAGAGAAGCCAGCAAACTATAATCCCGAATGTCGGGTCTAGACCCTGGGTAAGGGGTCTGTCTAGTAGAATAAGCATCTATTGGACAATAGTAAAACCGGGAGACATACTGTTAATTAATAGCAACGGGAACCTAATTGCTCCTCGGGGTTACTTCAAAATACGCACTGGGAAAAGCTCAATAATGAGGTCAGATGCACCCATTGGCACCTGCAGTTCTGAATGCATCACTCCAAATGGAAGCATTCCCAATGACAAGCCCTTTCAAAACGTAAACAAGATCACATATGGGGCATGTCCCAAGTATGTTAAGCAAAACACTCTGAAGTTGGCAACAGGGATGCGGAATGTACCAGAGAAACAAACTAGA

A/Albany/14/1978

CAAAACCTTCCCGGAAATGACAACAGCACAGCAACGCTGTGCCTGGGACATCATGCAGTGCCAAACGGAACGCTAGTGAAAACAATCACGAATGATCAGATTGAAGTGACTAATGCTACTGAGCTGGTTCAGAGTTCCTCAACAGGTAGAATATGCGACAGTCCTCATCGAATCCTTGATGGAAAAAACTGCACACTGATAGATGCTCTATTGGGAGACCCTCATTGTGATGGCTTTCAAAATGAGAAATGGGACCTTTTTGTTGAACGCAGCAAAGCTTTCAGCAACTGTTACCCTTATGATGTGCCAGATTATGCCTCCCTTAGGTCACTAGTTGCCTCGTCAGGCACCCTGGAGTTTATCAATGAAGGCTTCAATTGGACTGGGGTCACTCAGAATGGAGGAAGCTATGCTTGCAAAAGGGGACCTGATAACAGTTTCTTCAGTAGACTGAACTGGTTGTACGAATCAGAAAGCAAATATCCAGTGCTGAACGTGACTATGCCAAACAATGACAATTTTGACAAACTGTACATTTGGGGGGTTCACCACCCGAGCACGGACAAAGAACAAACCAACCTATATGTTCAAGCATCAGGGAGAGTCACAGTCTCTACCAAGAAAAGCCAGCAGACTATAATCCCGAATGTCGGGTCTAGACCCTGGGTAAGGGGTCTGTCTAGTAGAATAAGTATCTATTGGACAATAGTAAAACCGGGAGACATACTGTTAATTAATAGTAATGGGAACCTAATTGCTCCTCGGGGTTACTTCAAAATACGCACTGGGAAAAGCTCAATAATGAGGTCAGATGCACCTATTGGCACCTGCAGTTCTGAATGCATCACTCCAAATGGAAGCATTCCCAATGACAAGCCCTTTCAAAACGTAAACAAGATCACATATGGAGCATGTCCCAAGTATGTTAAGCAAAACACTCTGAAGTTGGCAACAGGGATGCGGAATGTACCAGAGAAACAAACTAGA

A/Bangkok/2/1979

CAAAACCTTCCCGGAAATGACAACAGCACAGCAACGCTGTGCCTGGGACACCATGCAGTGCCAAACGGAACGCTAGTGAAAACAATCACGAATGATCAGATTGAAGTGACTAATGCTACTGAGCTGGTTCAGAGTTCCTCAACAGGTAGAATATGCGACAGTCCTCACCGAATCCTTGATGGGAAAAACTGCACACTGATAGATGCTCTATTGGGAGACCCTCATTGTGATGGCTTTCAAAATGAGAAATGGGACCTTTTTGTTGAACGCAGCAAAGCTTTCAGCAACTGTTACCCTTATGATGTGCCAGATTATGCCTCCCTTAGGTCACTAGTTGCCTCGTCAGGCACCCTGGAGTTTATCAATGAAGGCTTCAATTGGACTGGGGTCACTCAGAGTGGGGGAAGCTATGCTTGCAAAAGGGGATCTGATAACAGTTTCTTCAGTAGACTGAATTGGTTGTACGAATCAGAAAGCAAATATCCAGTGCTGAACGTGACTATGCCAAACAATGGCAATTTTGACAAACTGTACATTTGGGGGGTTCACCACCCGAGCACATACAAAGAACAAACCAAGCTATATGTTCGAGCATCAGGGAGAGTCACAGTCTCTACCAAGAGAAGCCAGCAAACTATAATCCCGAATATCGGATCTAGACCCTGGGTAAGGGGTCTGTCTAGTAGAATAAGTATCTATTGGACAATAGTAAAACCGGGAGACATACTGTTAATTAATAGTAATGGGAACCTAATTGCTCCTCGGGGTTACTTCAAAATACGCACTGGGAAAAGCTCAATAATGAGGTCAGATGCACCTATTGGCACCTGCATTTCTGAATGCATCACTCCAAATGGAAGCATTCCCAATGACAAGCCCTTTCAAAACGTAAACAAGATCACATATGGGGCATGTCCCAAGTATGTTAAGCAAAACACTCTGAAGTTGGCAACAGGGATGCGGAATGTACCAGAGAAACAAACTAGA

A/Oregon/4/80

CAAAACCTTCCCGGAAATGACAACAGCACAGCAACGCTGTGCCTGGGACACCATACAGTGCCAAACGGAACGCTAGTGAAAACAATCACGAATGATCAGATTGAAGTGACTAATGCTACTGAGCTGGTTCAGAGTTCCTCAACAGGTAGAATATGCGACAGTCCTCACCGAATCCTTGATGGGAAAAACTGCACACTGGTAGATGCTCTATTGGGAGACCCTCATTGTGATGGCTTTCAGAATGAGAAATGGGACCTTTTTGTTGAACGCAGCAAAGCTTTCAGCAACTGTTACCCTTATGATGTGCCAGATTATGCCTCCCTTAGGTCACTAGTTGCCTCGTCAGGCACCCTGGAGTTTATCAATGAAAGCTTCAATTGGACTGGAGTCACTCAGAGTGGGGGAAGCTCTGCTTGCAAAAGGGGATCTGATAACAGTTTCTTCAGTAGACTGAATTGGTTGTACGAATCAGAAAGCAAATATCCAGTGCTGAACGTGACTATGCCAAACAATGGCAATTTTGACAAACTGTACATTTGGGGGGTTCACCACCCGAGCACGGACAAAGAACAAACCAACCTATATGTTCGAGCATCAGGGAGAGTCACAGTCTCTACCAAGAGAAGCCAGCAAACTATAATCCCGAATATCGGGTCTAGACCCTGGGTAAGGGGTCTGTCTAGCAGAATAAGCATCTATTGGACAATAGTAAAACCGGGAGACATACTGTTAATTAATAGTAATGGGAACCTAATTGCTCCTCGGGGTTACTTCAAAATACGCACTGGGAAAAGCTCAATAATGAGGTCAGATGCACCTATTGGCACCTGCAGTTCTGAATGCATCACTCCAAATGGAAGCATTCCCAATGACAAGCCCTTTCAAAATGTAAACAAGATCACATATGGGGCATGTCCCAGGTATGTTAAGCAAAACACTCTGAAGTTGGCAACAGGGATGCGGAATGTACCAGAGAAACAAACTAGA

A/Belgium/2/81

CAAAAACTTCCCGGAAATGACAACAGCACAGCAACGCTGTGCCTGGGACACCATGCAGTGCCAAACGGAACGCTAGTGAAAACAATCACGAATGATCAGATTGAAGTGACTAATGCTACTGAGCTGGTTCAGAGTTCCTCAACAGGTAGAATATGCGACAGTCCTCATCGGATCCTTGATGGGAAAAACTGCACACTGGTAGATGCTCTATTGGGAGACCCTCATTGTGATGGCTTTCAAAATGAGAAATGGGACCTTTTTGTTGAACGCAGCAAAGCTTTCAGCAACTGTTACCCTTATGATGTGCCAGATTATGCCTCCCTTAGGTCACTAGTTGCCTCGTCAGGCACCCTGGAGTTTATCAATGAAAGCTTCAATTGGACTGGAGTCACTCAGAGTGGGGGAAGCTATGCTTGCAAAAGGGGATCTGATAAAAGTTTCTTCAGTAGACTGAATTGGTTGTACGAATCAGAAAGCAGATATCCAGTGCTGAACGTGACTATGCCAAACAATGGCAATTTTGACAAACTGTACATTTGGGGGGTTCACCACCCGAGCACGGACAAAGAACAAACCAACCTATATGTTCGAGCATCAGGGAGAGTCACAGTCTCTACCAAGAGAAGCCAGCAAACTATAATCCCGAATATCGGGTCTAGACCCTGGGTAAGGGGTCTGTCTAGCAGAATAAGCATCTATTGGACAATAGTAAAACCGGGAGACATACTGTTAATTAATAGTAATGGGAACCTAATTGCTCCTCGGGGTTACTTCAAAATGCGCACTGGGAAAAGCTCAATAATGAGGTCAGATGCACCTATTGGCACCTGCAGTTCTGAATGCATCACTCCAAATGGAAGCATTCCCAATGACAAGCCCTTTCAAAATGTAAACAAGATCACATATGGGGCATGTCCCAAGTATGTTAAGCAAAACACTCTGAAGTTGGCAACAGGGATGCGGAATGTACCAGAGAAACAAACTAGA

A/Philippines/2/82

CAAAACCTTCCCGGAAATGACAACAGCACAGCAACGCTGTGCCTGGGACATCATGCAGTGCCAAACGGAACGCTAGTGAAAACAATCACGAATGATCAGATTGAAGTGACTAATGCTACTGAGCTGGTTCAGAGTTCCTCAACAGGTAGGATATGCGACAGTCCTCACCGAATCCTTGATGGGAAAAACTGCACACTGATAGATGCTCTATTGGGAGACCCTCATTGTGATGGCTTCCAAAATGAGAAATGGGACCTTTTTGTTGAACGCAGCAAAGCTTTCAGCAACTGTTACCCTTATGATGTGCCAGATTATGCCTCCCTTAGGTCACTAGTTGCCTCATCAGGCACCCTGGAGTTTATCAATGAAGGCTTCAATTGGACTGGAGTCACTCAGAGTGGGGGAAGCTATACTTGCAAAAGGGGATCTAATAACAGTTTCTTCAGTAGACTGAACTGGTTGTACGAATCAGAAAGCAAATATCCAGTGCTGAACGTGACTATGCCAAACAATGGCAAATTTGACAAATTGTACATTTGGGGGATTCACCACCCGAGCACGGACAAAGAACAAACCAACCTATATATTCGAGCATCAGGGAGAGTCACAGTCTCTACCAAGAGAAGCCAGCAAACTGTAATCCCGAATATCGGGTCTAGACCCTGGGTAAGGGGTCTGTCTAGTAGAATAAGTATCTATTGGACAATAGTAAAACCGGGAGACATACTGTTAATTAATAGCACTGGGAACCTAATTGCTCCTCGGGGTTACTTCAAAATACGCACTGGGAAAAGCTCAATAATGAGGTCAGATGCACCTATTGGCACCTGCAGTTCTGAATGCATCACTCCAAATGGAAGCATTCCCAATGACAAGCCCTTTCAAAACGTAAACAAGATCACATATGGGGCATGTCCCAGGTATGTTAAGCAAAACACTCTGAAGTTGGCAACAGGGATGCGGAATGTACCAGAGAAACAAACTAGA

A/Oslo/13676/83

CAAAACCTTCCCGGAAATGACAACAGCACGGCAACGCTGTGCCTGGGACACCATGCAGTGCCAAACGGAACGCTAGTGAAAACAATCACGAATGATCAGATTGAAGTGACTAATGCTACTGAGCTGGTTCAGAGTTCCTCAACAGGTAGAATATGCGACAGTCCTCACCGAATCCTTGATGGGAAAAACTGCACACTGGTAGATGCTCTATTGGGAGACCCTCATTGTGATGGCTTTCAAAATGAGAAATGGGACCTTTTTGTTGAACGCAGCAAAGCTTTCAGCAACTGTTACCCTTATGATGTGCCAGATTATGCCTCCCTTAGGTCACTAGTTGCCTCGTCAGGCACCCTGGAGTTTACCAATGAAAGCTTCAATTGGACTGGAGTCACTCAGAGTGGGGGAAGCTATGCTTGCAAAAGGGGATCTGATAACAGTTTCTTCAGTAGACTGAATTGGTTGTACGAATCAGAAAGCAAATATCCAGTGCTGAACGTGACTATGCCAAACAATGGCAATTTTGACAAACTGTACATTTGGGGGGTTCACCACCCGAGCACGGACAAAGAACAAACCAACCTATATGTTCGAGCATCAGGGAGAGTCACAGTCTCTACCAAGAGAAACCAGCAAACTGTAATCCCGAATATCGGGTCTAGACCCTGGGTAAGGGGTCTGTCTAGCAGAATAAGCATCTATTGGACAATAGTAAAACCGGGAGACATACTGCTAATTAATAGTAACGGGAACCTAATTGCTCCTCGGGGTTACTTCAAAATACGCAATGGGAAAAGCTCAATAATGAGGTCAGATGCACCTATTGGCACCTGCAGTTCTGAATGCATCACTCCAAATGGAAGCATTCCCAATGACAAGCCCTTTCAAAATGTAAACAAGATCACATATGGGGCATGTCCCAAGTACGTTAAGCAAAATACTCTGAAGTTGGCAACAGGGATGCGGAATGTACCAGAGAAACAAACTAGA

A/Caen/1/84

CAAAAACTTCCCGGAAATGACAACAGCACAGCAACGCTGTGCCTGGGACATCATGCAGTGCCAAACGGAACGCTAGTGAAAACAATCACGAATGATCAGATTGAAGTGACTAATGCTACTGAGCTGGTTCAGAGTTCCTCAACAGGTAGAATATGCGACAGTCCTCACCGAATCCTTGATGGGAAAAACTGCACACTGATAGATGCTCTATTGGGAGACCCTCATTGTGATGGCTTCCAAAATGAGAAATGGGACCTTTTTGTTGAACGCAGCAAAGCTTACAGCAACTGTTACCCTTATGATGTGCCGGATTATGCCTCCCTTAGGTCACTAGTTGCCTCATCAGGCACCCTGGAGTTTATCAACGAAGGCTTCAATTGGACTGGAGTCACTCAGAGTGGGGGAAGCTATGCTTGCAAAAGGGGATCTGTTAACAGTTTCTTCAGTAGATTGAATTGGTTGTACAAATCAGAAAGCAAATATCCAGTGCTGAACGTGACTATGCCAAACAATGGCAAATTTGACAAATTGTACATTTGGGGGGTTCACCACCCGAGCACGGACAAAGAACAAACCAACCTATATGTTCGAGCATCAGGGAGAGTCACAGTCTCTACCAAGAGAAGCCAGCAAACTGTAATCCCGAATATCGGGTCTAGACCCTGGGTAAGGGGTCTGTCTAGTAGAATAAGTATCTATTGGACAATAGTAAAACCGGGAGACATACTGTTAATTAATAGCACTGGGAACCTAATTGCTCCTCGGGGTTACTTCAAAATACGCACTGGGAAAAGCTCAATAATGAGGTCAGATGCACCTATTGGCACCTGCAGTTATGAATGCATCACTCCAAATGGAAGCATTCCCAATGACAAACCCTTTCAAAATGTAAACAAGATCACATATGGGGCATGTCCCAGGTATGTTAAGCAAAACACTCTGAAGTTGGCAACAGGGATGCGGAATGTACCAGAGAAACAAACTAGA

A/Stockholm/4/1985

CAAAAACTTCCCGGAAATGACAACAGCACAGCAACGCTGTGCCTGGGACATCATGCAGTACCAAACGGAACGCTAGTGAAAACAATCACGAATGAACAGATTGAAGTGACTAATGCTACTGAGCTGGTTCAGAGTTCCTCAACAGGTAGAATATGCGACAGTCCTCACCGAATCCTTGATGGAAAAAACTGCACACTGATAGATGCTCTATTGGGAGACCCTCATTGTGATGGCTTCCAAAATGAGAAATGGGATCTTTTTGTTGAACGCGGCAAAGCTTTCAGCAACTGCTACCCTTATGATGTGCCGGATTATGCCTCCCTTAGGTCACTAGTTGCCTCGTCAGGCACCCTGGAGTTTATCAATGAAGGCTTCAATTGGACTGGAGTCACTCAGAGTGGGGGAAGCTATGCTTGCAAAAGGGGATCTGTTAACAGTTTCTTCAGTAGATTGAATTGGTTGTATAAGTCAGAAAGCAAATATCCAGCGCTGAACGTGACTATGCCAAACAATGGCAAATTTGACAAATTGTACATTTGGGGGGTTCACCACCCTAGCACAGACAAAGAACAAACCAACCTATATGTTCGAGCATCAGGGAGAGTCACAGTCTCTACCAAGAGAAGCCAGCAAACTGTAATCCCGAATATCGGGCCTAGACCCTGGGTAAGGGGTCTGTCTAGTAGAATAAGTATCTATTGGACAATAGTAAAACCGGGAGACATACTGTTAATTAATAGCACTGGGAACCTAATTGCTCCCCGGGGTTACTTCAAAATACACACTGGGAAAAGCTCAATAATGAGGTCAGATGCACCTATTGGCACCTGCAGTTCTGAATGCATCACTCCAAATGGAAGCATTCCCAATGACAAACCCTTTCAAAATGTAAACAAGATCACATATGGGGCATGTCCCAGGTATGTTAAGCAAAACACTCTGAAGTTGGCAACAGGGATGCGGAATGTACCAGAGAAACAAACTAGA

A/Czechoslovakia/4/86

CAAAAACTTCCCGGAAATGACAACAGCACAGCAACGCTGTGCCTGGGACATCATGCAGTGCCAAACGGAACGCTAGTGAAAACAATCACGAATGATCAGATTGAAGTGACTAATGCTACTGAGCTGGTTCAGAGTTCCTCAACAGGTAGAATATGCGACAGTCCTCACCGAATCCTTGATGGAAAAAACTGCACACTGATAGATGCTCTATTGGGAGACCCTCATTGTGATGGCTTCCAAAATGAGAAATGGGACCTTTTTATTGAACGCAGCAAAGCTTTCAGCAACTGTTACCCTTATGATGTGCCGGATTATGCCTCCCTTAGGTCACTAGTTGCCTCATCAGGCACCCTGGAGTTTATCAATGAAGGCTTCAATTGGACTGGAGTCACTCAGAGTGGGGGAAGCTATGCTTGCAAAAGGGGATCTGTTAACAGTTTCTTCAGTAGATTGAATTGGTTGTACGAATCAGAATACAAATATCCAGCGCTGAACGTGACTATGCCAAACAATGGCAAATTTGACAAACTGTACATTTGGGGGGTTCACCACCCGATCACGGAAAAAGAACAAACCAACCTATATGTTCGAGCATCAGGGAGAGTCACAGTCTCTACCAAGAGAAGCCAGCAAACTGTAATCCCGAATATCGGGTCTAGACCCTGGGTAAGGGGTCTGTCTAGTAGAATAAGCATCTATTGGACAATAGTAAAACCGGGAGACATACTGTTAATTAATAGCACTGGGAACCTAATTGCTCCTCGGGGTTACTTCAAAATACGCACTGGGAAAAGCTCAATAATGAGGTCAGATGCACCTATTGGCACCTGCAGTTCTGAATGCATCACTCCAAATGGAAGCATTCCCAATGACAAACCCTTTCAAAATGTAAACAAGATCACATATGGGGCATGTCCCAGGTATGTTAAGCAAAACACTCTGAAATTGGCAACAGGGATGCGGAATGTACCAGAGAAACAAACTAGA

A/Shanghai/11/1987

CAAAAACTTCCCGGAAATGACAACAGCACAGCAACGCTGTGCCTGGGACATCATGCAGTGCCAAACGGAACGCTAGTGAAAACAATAACGAATGATCAGATTGAAGTGACTAATGCTACTGAGCTGGTTCAGAGTTCCTCAACAGGTAGAATATGCGACAGTCCTCACCGAATCCTTGATGGAAAAAACTGCACACTGATAGATGCTCTATTGGGAGACCCTCATTGTGATGGCTTCCAAAATGAGAAATGGGACCTTTTTGTTGAACGCAGCAAAGCTTACAGCAACTGTTACCCTTATGATGTGCCGGATTATGCCTCCCTTAGGTCACTAGTTGCCTCATCAGGCACCCTGGAGTTTATCAATGAAGACTTCAATTGGACTGGAGTCACTCAGAGTGGGGGAAGCTATGCTTGCAAAAGGGGATCTGTTAAAAGTTTCTTCAGTAGATTGAATTGGTTGCACGAATCAGAAGACAAATATCCAGCGCTGAACGTGACTATGCCAAACAATGGCAAATTTGACAAATTGTACATTTGGGGGGTTCACCACCCGAGCACGGACAGAGAACAAACCAAACTATATGTTCGAGCATCAGGGAGAGTCACAGTCTCTACCAAGAGAAGCCAGCAAACTGTAATCCCGAATATCGGGTCTAGACCCTGGGTAAGGGGTCTGTCCAGTAGAATAAGCATCTATTGGACAATAGTAAAACCGGGAGACATACTGTTGATTAATAGCACCGGGAACCTAATTGCTCCTCGGGGTTACTTCAAAATACGCACTGGGAAAAGCTCAATAATGAGGTCAGATGCACCTATTGGCACCTGCAGTTCTGAATGCATCACTCCAAATGGAAGCATTCCCAATGACAAACCCTTTCAAAATGTAAACAAGATCACATATGGGGCATGTCCCAGGTATGTTAAGCAAAACACTCTGAAATTGGCAACAGGGATGCGGAATGTACCAGAGAAACAAACTAGA

A/Uruguay/3/88

CAGAAAATGCCCGGAAATGACAACAGCACAGCAACGCTGTGCCTGGGACACCATGCAGTGCCAAACAGAACGCTAGTGAAAACAATCACGAATGATCAGATTGAAGTGACTAATGCTACTGAGCTGGTTCAGAGTTCCTCAACAGGTAGAATATGCGACAGTCCTCACCGAATCCTTGATGGAAAAAACTGCACACTGATAGATGCTCTATTGGGAGACCCTCATTGTGATGGCTTCCAAAATGAGAAATGGGACCTTTTTGTTGAACGCAGCAAAGCTTTCAGCAACTGTTACCCTTATGATGTGCCGGATTATGCCTCCCTTAGGTCACTAGTTGCCTCATCAGGCACCCTGGAGTTTATCAATGAAGACTTCAATTGGACTGGAGTCACTCAGAGTGGGGGAAGCTGTGCTTGCAAAAGGGGATCTGTTAACAGTTTCTTCAGTAGATTGAATTGGTTGTACGAATCAGAATACAAATATCCAGCGCTGAACGTGACTATGCCAAACAATGGCAAATTTGACAAATTGTACATTTGGGGGGTTCACCACCCGAGCACGGACAAAGAACAAACCAACCTATATGTTCGAGCATCAGGGAGAGTCATAGTCTCTACCAAGAGAAGCCAGCAAACTGTAGTCCCGAATATCGGGTCTAGACCCTGGGTAAGAGGTCTGTCCAGTAGAATAAGCATCTATTGGACAATAGTAAAACCGGGAGACATACTGCTGATTAGTAGCACTGGGAACCTAATTGCTCCTCGGGGTTACTTCAAAATACGCACGGGGAAAAGCTCAATAATGAGGTCAGATGCACCTATTGGCACCTGCAGTTCTGAATGCATCACTCCAAATGGAAGCATTCCCAATGACAAACCCTTTCAAAATGTAAACAGGATCACATATGGGGCATGTCCCAGGTATGTCAAGCAAAACACCCTAAAATTGGCAACAGGGATGCGGAATGTGCCAGAGAAACAAACTAGA

A/England/648/89

CAAAAACTTCCCGGAAATGACAACAGCACAGCAACGCTGTGCCTAGGACATCATGCAGTGCCAAACGGAACGCTAGTGAAAACAATCACGAATGACCAAATTGAAGTGACTAATGCTACTGAGCTGGTTCAGAGTTCCTCAACAGGTCGAATATGCGACAGTCCTCACCGAATCCTTGATGGAAAAAACTGCACACTGATAGATGCTCTATTGGGAGACCCTCATTGTGATGGCTTCCAAAATAAGGAATGGGACCTTTTTGTTGAACGCAGCAAAGCTTACAGCAACTGTTACCCTTATGATGTGCCGGATTATGCCTCCCTTAGGTCCCTAGTTGCCTCATCAGGCACCCTGGAGTTTATCAATGAAGACTTCAATTGGACTGGAGTCGCTCAGAGTGGGGGAAGCTATGCTTGCAAAAGGGGATCTGTTAAAAGTTTCTTCAGTAGATTGAATTGGTTGCACGAATCAGAATACAAATATCCAGCGCTGAACGTGACTATGCCAAACAATGGCAAATTTGACAAATTGTACATTTGGGGGGTTCACCACCCGATCACGGACAGAGAACAAACCAACCTATATATTCGAGCATCAGGGAGAGTCACAGTCTCTACCAAAAGAAGCCAGCAAACTGTAATCCCGAATATCGGGTCTAGACCCTGGGTAAGGGGTCTGTCCAGTAGAATAAGCATCTATTGGACAATAGTAAAACCGGGAGACATACTGTTGATTAATAGCACCGGGAACCTAATTGCTCCTCGGGGTTACTTCAAAATACGAACTGGAAAAAGCTCAATAATGAGGTCAGATGCACCCATTGGCACCTGCAGTTCTGAATGCATCACTCCAAATGGAAGCATTCCCAATGACAAACCCTTTCAAAATGTAAACAGGATCACATATGGGGCATGTCCCAGATATGTTAAGCAAAACACTCTGAAATTGGCAACAGGGATGCGGAATGTACCAGAGAAACAAACTAGA

A/Victoria/2/90

CAAAAACTTCCCGGAAATGACAACAGCACAGCAACGCTGTGCCTGGGACATCATGCAGTGCCAAACGGAACGCTAGTGAAAACAATCACGAATGATCAAATTGAAGTGACTAATGCTACTGAGCTGGTTCAGAGTTCCTCAACAGGTAGAATATGCGACAGTCCTCACCGAATCCTTGATGGAAAAAACTGCACACTGATAGATGCTCTATTGGGAGACCCTCATTGTGATGGCTTCCAAAATAAGGAATGGGACCTTTTTATTGAACGCAGCAAAGCTTACAGCAACTGTTACCCTTATGATGTGCCGGATTATGCCTCCCTTAGGTCACTAGTTGCCTCATCAGGCACCCTGGAGTTTATCAATGAAGACTTCAATTGGACTGGAGTCGCTCAGAGTGGGGAAAGCTATGCTTGCAAAAGGGGATCTGTTAAAAGTTTCTTTAGTAGATTGAATTGGTTGCACGAATCAGAATACAAATATCCAGCGCTGAACGTGACTATGCCAAACAATGGCAAATTTGACAAATTGTACATTTGGGGGGTTCACCACCCGAGCACGGACAGAGAACAAACCAGCCTATATGTTCGAGCATCAGGGAGAGTCACAGTCTCTACCAAAAGAAGCCAACAAACTGTAATCCCGAATATCGGGTCCAGACCCTGGGTAAGGGGTCTGTCCAGTAGAATAAGCATCTATTGGACAATAGTAAAACCGGGAGACATACTTTTGATTAATAGCACCGGGAACCTAATTGCTCCTCGGGGTTACTTCAAAATACGAACTGGGAAAAGCTCGATAATGAGGTCAGATGCACCCATTGGCACCTGCAGTTCTGAATGCATCACTCCAAATGGAAGCATCCCCAATGACAAACCTTTTCAAAATGTAAACAGGATCACATATGGGGCATGTCCCAGATATGTTAAGCAAAACACTCTGAAATTGGCAACAGGGATGCGGAATGTACCAGAGAAACAAACTAGA

A/Brazil/02/91

CAAAAACTTCCCGGAAATGACAACAGCACAGCAACGCTGTGCCTGGGACATCATGCAGTGCCAAACGGAACGCTAGTGAAAACAATCACGAATGATCAAATTGAAGTGACTAATGCTACTGAGCTGGTTCAGAGTTCCTCAACAGGTAGAATATGCGACAGTCCTCACCGAATCCTTGATGGAAAAAACTGCACACTGATAGATGCTCTATTGGGAGACCCTCATTGTGATGGCTTCCAAAATAAGGAATGGGACCTTTTTGTTGAACGCAGCAAAGCTTACAGCAACTGTTACCCTTATGATGTGCCGGATTATGCCTCCCTTAGGTCACTAGTTGCCTCATCAGGCACCCTGGAGTTCATCAATGAAGACTTCAATTGGACTGGAGTCGCTCAGAGTGGGGAAAGCTATGCTTGCAAAAGGGGATCTGTTAAAAGTTTCTTTAGTAGATTGAATTGGTTGCACGAATCAGAATACAAATATCCAGCGCTGAACGTGACTATGCCAAACAATGGCAAATTTGACAAATTGTACATTTGGGGGGTTCACCACCCGATCACGGACAGAGAACAAACCAGCCTATATGTTCGAGCATCAGGGAGAGTCACAGTCTCTACCAAAAGAAGCCAACAAACTGTAATCCCGAATATCGGGTCTAGACCCTGGGTAAGGGGTCTGTCTAGTAGAATAAGCATCTATTGGACAATAGTAAAACCGGGAGACATACTTTTGATTAATAGCACCGGGAACCTAATTGCTCCTCGGGGTTACTTCAAAATACGAACTGGGAAAAGCTCGATAATGAGATCAGATGCACCCATTGGCACCTGCAATTCTGAATGCATCACTCCAAATGGAAGCATCCCCAATGACAAACCTTTTCAAAATGTAAACAGGATCACATATGGGGCATGTCCCAGATATGTTAAGCAAAACACTCTGAAATTGGCAACAGGGATGCGGAATGTACCAGAGAAACAAACTAGA

A/Houston/56829/92

CAAAAACTTCCCGGAAATGACAACAGCACAGCAACGCTGTGCCTGGGACATCATGCAGTGCCAAACGGAACGCTAGTGAAAACAATCACGAATGATCAAATTGAAGTGACTAATGCTACTGAGCTGGTTCAGAGTTCCTCAACAGGTAGAATATGTGACAGTCCTCACCGAATCCTTGATGGAAAAAATTGCACACTGATAGATGCTCTATTGGGAGACCCTCATTGTGATGGCTTCCAAAATAAGGAATGGGACCTTTTTGTTGAACGCAGCAAAGCTTACAGCAACTGTTACCCTTATGAAGTGCCGGATTATGCCTCCCTTAGGTCACTAGTTGCCTCATCAGGCACCCTGGAGTTTACCAATGAAGACTTCAATTGGACTGGAGTCGCTCAGAGTGGGGAAAGCTATGCTTGCAAAAGGGGATCTGTTAAAAGTTTCTTTAGTAGATTGAATTGGTTGCACGAATCAGATTACAAATATCCAGCGCTGAACGTGACTATGCCAAACAATGGCAAATTTGACAAATTGTACATTTGGGGGGTTCACCACCCGAGCACGGACAGAGAACAAACCAGCCTATATGTTCGAGCATCAGGGAGAGTCACAGTCTCCACCAAAAGAAGCCAACAAACTGTGATCCCGAATATCGGGTCCAGACCCTGGGTAAGGGGTCTGTCCAGTAGAATAAGCATCTATTGGACAATAGTGAAACCGGGAGACATACTTTTGATTAATAGCACCGGGAACCTAATTGCTCCTCGGGGTTACTTCAAAATACGAACTGGGAAAAGCTCGATAATGAGATCAGATGCACCCATTGGCACCTGCAGTTCTGAATGCATCACTCCAAATGGAAGCATCCCCAATGACAAACCTTTTCAAAACGTAAACAGGATCACATATGGGGCATGTCCCAGATATGTTAAGCAAAACACTCTGAAATTGGCAACAGGGATGCGGAATGTACCAGAGAAACAAACTAGA

A/Spain/118/93

CAAAAACTTCCCGGAAATGACAACAGCACAGCAACGCTGTGCCTGGGGCATCATGCAGTGCCAAACGGAACTCTAGTGAAAACAATCACGAATGATCAAATTGAAGTGACTAATGCTACTGAGCTGGTCCAGAGTTCCTCAACAGGTAGAATATGCGACAGTCCTCACCGAATCCTTGATGGAAAAAACTGCACACTGATGGATGCTCTATTGGGAGACCCTCATTGTGATGGCTTCCAAAATAAGGAATGGGACCTTTTTGTTGAACGCAACAAAGCTTACAGCAATTGTTACCCTTATGATGTGCCAGATTATGCCTCCCTTAGGTCACTAGTTGCCTCATCAGGCACCCTGGAGTTTATCAATGAAGACTTCAATTGGACTGGAGTTGCTCAGAATGGGGACAGCTATGCTTGCAAAAGGGGATCTGTTAAAAGTTTCTTTAGTAGATTGAATTGGTTGCACGAATCAGAATACAAATATCCAGCGCTGAACGTGACTATGCCAAACAATGACAAATTTGACAAGTTGTACATTTGGGGGGTTCACCACCCGAGCACGGACAGAGAACAAACCAGCCTATATATTCGAGCATCAGGGAGAGTCACAGTCTCTACCAAAAGAAGCCAACAAACTGTAATCCCGAATATCGGGTCCAGACCCTGGGTAAGGGGTCTGTCCAGTAGAATAAGCATCTATTGGACAATAGTAAAACCGGGAGACATACTTTTGATTAATAGCACCGGGAATCTAATTGCTCCTCGGGGTTACTTCAAAATACGAACTGGGAAAAGCTCGATAATGAGGTCAGATGCACCCATTGGCACCTGCAGTTCTGAATGCATCACTCCAAATGGAAGCATCCCCAATGACAAACCTTTTCAAAATGTAAACAGGATCACATATGGGGCATGTCCAAGATATGTTAAGCAAAACACTCTGAAATTGGCAACAGGGATGCGGAATGTACCAGAGAAACAAACTAGA

A/France/1203/94

CAAAAACTTCCCGGAAATGACAACAGCACAGCAACGCTGTGCCTGGGACACCATGCAGTGCCAAACGGAACGCTAGTGAAAACAATCACGAATGATCAAATTGAAGTGACTAATGCTACTGAGCTGGTTCAGAGTTCCTCAACAGGTAGAATATGCGACAGTCCTCACCGAATCCTTGATGGAAAAAACTGCACACTGATAGATGCTCTATTGGGAGACCCTCATTGTGATGGCTTCCAAAATAAGGAATGGGACCTTTTTGTTGAACGCAGCAAAGCTTACAGCAACTGTTACCCTTATGATGTGCCGGATTATGCCTCCCTTAGGTCACTAGTTGCCTCATCAGGCACCCTGGAGTTTATCAATGAAGACTTCAATTGGACTGGAGTCGCTCAGGATGGGAAAAGCTATGCTTGCAAAAGGGGATCTGTTAAAAGTTTCTTTAGTAGATTGAATTGGTTGCACAAATTAGAATACAAATATCCAGCGCTGAACGTGACTATGCCAAACAATGGCAAATTTGACAAATTGTACATTTGGGGGGTCCACCACCCGAGCACGGACAGTGACCAAACCAGCCTATATGTTCGAGCATCAGGGAAAGTCACAGTCTCTACCAAAAGAAGCCAACAAACTGTAATCCCGAATATCGGGTTTAGACCCTGGGTAAGGGGTCTGTCCAGTAGAATAAGCATCTATTGGACAATAGTAAAACCGGGAGACATACTTTTGATTAATAGCACAGGGAATCTAATTGCTCCTAGGGGTTACTTCAAAATACGAAATGGGAAAAGCTCAATAATGAGGTCAGATGCACCCATTGGCAACTGCAGTTCTGAATGCATCACTCCAAATGGAAGCATTCCCAATGACAAACCTTTTCAAAATGTAAACAGGATCACATATGGGGCCTGCCCCAGATATGTTAAGCAAAACACTCTGAAGTTGGCAACAGGGATGCGGAATGTACCAGAGAAACAAACTAGA

A/Memphis/24/95

CAAAAACTTCCCGGAAATGACAACAGCACAGCAACGCTGTGCCTGGGACACCATGCAGTGCCAAACGGAACGCTAGTGAAAACAATCACGAATGATCAAATTGAAGTGACTAATGCTACTGAGCTGGTTCAGAGTTCCTCAACAGGTAGAATATGCGACAGTCCTCACCGAATCCTTGATGGAAAAAACTGCACACTGATAGATGCTCTATTGGGAGACCCTCATTGTGATGGCTTCCAAAATAAGGAATGGGACCTTTTTGTTGAACGCAGCAAAGCTTACAGCAACTGTTACCCTTATGATGTGCCGGATTATGCCTCCCTTAGGTCACTAGTTGCCTCATCAGGCACCCTGGAGTTTACCAAGGAAGGCTTCAATTGGACTGGAGTCGCTCAGGATGGGAAAAGCTATGCTTGCAAAAGGGGATCTGTTAACAGTTTCTTTAGTAGATTGAATTGGTTGCACAAATTAGAATACAAATATCCAGCACTGAACGTGACTATGCCAAACAATGACAAATTTGACAAATTGTACATTTGGGGGGTTCACCACCCGAGCACGGACAGTGACCAAACCAGCCTATATGTTCAAGCATCAGGGAGAGTCACAGTCTCTACCAAAAGAAGCCAACAAACTGTAATCCCGAATATCGGGTCTAGACCCTGGGTAAGGGGTATCTCCAGTAGAATAAGCATCTATTGGACAATAGTAAAACCGGGAGACATACTTTTGATTAACAGCACAGGGAATCTAATTGCTCCTCGGGGTTACTTCAAAATACGAAATGGGAAAAGCTCAATAATGAGGTCAGATGCACCCATTGGCAACTGCAATTCTGAATGCATCACTCCAAATGGAAGCATTCCCAATGACAAACCTTTTCAAAATGTAAACAGGATCACATATGGGGCCTGTCCCAGATATGTTAAGCAAAACACTCTGAAGTTGGCAACTGGGATGCGGAATGTACCAGAGAAACAAACTAGA

A/Brisbane/8/96

CAAAAACTTCCCGGAAATGACAACAGCACGGCAACGCTGTGCCTGGGACACCATGCAGTGCCAAACGGAACGCTAGTGAAAACAATCACGAATGACCAAATTGAAGTGACTAATGCTACTGAGCTGGTTCAGAGTTCCTCAACAGGTAGAATATGCGACAGTCCTCACCGAATCCTTGATGGAAAAAACTGCACACTGATAGATGCTCTATTGGGAGACCCTCATTGTGATGGCTTCCAAAATAAGGAATGGGACCTTTTTGTTGAACGCAGCAAAGCTTACAGCGACTGTTACCCTTATGATGTGCCGGATTATGCTTCCCTTAGGTCACTAGTTGCCTCATCCGGCACCCTGGAGTTTACCAATGAAGGCTTCAATTGGACTGGAGTCGCTCAGGATGGAACAAGCTATGCTTGCAAAAGGGGATCTGTTAAAAGTTTCTTTAGTAGATTGAATTGGTTGCACAAATTAGAATACAAATATCCAGCACTGAACGTGACTATGCCAAACAATGACAAATTTGACAAATTGTACATTTGGGGGGTTCACCACCCGAGTACGGACAGTGACCAAACCAGCCTATATGTTCAAACATCAGGGAGAGTCACAGTCTCTACCAAAAGAAGCCAACAAACTGTAATCCCGAATATCGGGTCTAGACCCTGGGTAAGGGGGGTCTCCAGCGGAATAAGCATCTATTGGACAATAGTAAAACCGGGAGACATACTTTTGATTAATAGCACAGGGAATCTAATTGCCCCTCGGGGTTACTTCAAAATACGAAGTGGGAAAAGCTCAATAATGAGGTCAGATGCACCCATTGGCAACTGCAATTCTGAATGCATCACTCCAAATGGAAGCATTCCCAATGACAAACCTTTTCAAAATGTAAACAGGATCACATATGGGGCCTGTCCCAGATATGTTAAGCAAAACACTCTGAAATTGGCAACAGGGATGCGGAATGTACCAGAGAAACAAACTAGA

A/Russia/41/97

CAAAAACTTCCCGGAAATGACAACAGCACGGCAACGCTGTGCCTGGGACACCATGCAGTGCCAAACGGAACGCTAGTGAAAACAATCACGAATGACCAAATTGAAGTGACTAATGCTACTGAGCTGGTTCAGAGTTCCTCAACAGGTAGAATATGCGACAGTCCTCACCGAATCCTTGATGGAAAAAACTGCACACTGATAGATGCTCTATTGGGAGACCCTCATTGTGATGACTTCCAAAATGAGGAATGGGACCTTTTTGTTGAACGCAGCAAAGCCTACAGCAACTGTTACCCTTATGATGTGCCGGATTATGCCTCCCTTAGGTCACTAGTTGCCTCATCCGGCACCCTGGAGTTTAACAATGAAAGCTTCAATTGGATTGGAGTCGCTCAGAATGGAACAAGCTATGCTTGCAAAAGGAGATCTGTTAAAAGTTTCTTTAGTAGATTGAATTGGTTGCACAAATTAGAATACAAATATCCAGCACTGAACGTGACTATGCCAAACAATGACAAATTTGACAAATTGTACATTTGGGGGGTTCACCACCCGAGTACGGACAGTGACCAAACCAGCCTATATGCTCAAACATCAGGGAGAGTCACAGTCTCTACCAAAAGAAGCCAACAAACTGTAATCCCGAATATCGGATCTAAACCCTGGGTAAGGGGTATCTCCAGCAGAATAAGCATCTATTGGACAATAGTAAAACCGGGAGACATACTTTTGATTACCAGCACAGGGAATCTAATTGCTCCTCGGGGTTACTTCAAAATACGAAGTGGGAAAAGCTCAATAATGAGGTCAGATGCACCCATTGGCAAATGCAATTCTGAATGCATCACTCCAAATGGAAGCATTCCCAATGACAAACCATTTCAAAATGTAAACAGGATCACATATGGGGCCTGTCCCAGATATGTTAAGCAAAACACTCTGAAATTGGCAACAGGGATGCGGAATGTACCAGAGAAACAAACTAGA

A/Zhejiang/18/98

CAAAAACTTCCCGGAAATGACAACAGCACGGCAACGCTGTGCCTGGGACACCATGCAGTGCCAAACGGAACGCTAGTGAAAACAATCACGAGTGACCAAATTGAAGTGACTAATGCTACTGAGCTGGTTCAGAGTTCCTCAACAGGTAGAATATGCGACAGTCCTCACCGAATCCTTGATGGAGAAAACTGCACACTGATAGATGCTCTATTGGGAGACCCTCATTGTGATGGCTTCCAAAATAAGGAATGGGACCTTTTTGTTGAACGCAGCAAAGCCTACAGCAACTGTTACCCTTATGATGTGCCGGATTATGCCTCCCTTAGGTCACTAGTTGCCTCATCCGGCACCCTGGAGTTTAACAATGAAAGCTTCAATTGGACTGGAGTCGCTCAGAATGGAACAAGCTTTGCTTGCAAAAGGAGATCTATTAAAAGTTTCTTTAGTAGATTGAATTGGTTGCACCAATTAAAATACAAATATCCAGCACTGAACGTGACTATGCCAAACAATGACAAATTTGACAAATTGTACATTTGGGGGGTTCACCACCCGAGTACGGACAGTGACCAAACCAGCCTATATGCTCAAGCATCAGGGAGAGTCACAGTCTCTACCAAAAGAAGCCAACAAACTGTAATCCCGAATATCGGATCTAGACCCTGGGTAAGGGGTGTCTCCAGCAGAATAAGCATCTATTGGACAATAGTAAAACCGGGAGACATACTTCTGATTAACAGCACAGGGAATCTAATTGCTCCTCGGGGTTACTTCAAAATACGAAGTGGGAAAAGCTCAATAATGAGGTCAGATGCACCCATTGGCAAATGCAATTCTGAGTGCATCACTCCAAATGGAAGCATTCCCAATGACAAACCATTTCAAAATGTAAACAGGATCACATATGGGGCCTGTCCCAGATATGTTAAGCAAAACACTCTGAAATTGGCAACAGGGATGCGGAATGTTCCAGAGAAACAAACTAGA

A/Moscow/10/99

CAAAAACTTCCCGGAAATGACAACAGCACGGCAACGCTGTGCCTGGGACACCATGCAGTGCCAAACGGAACGCTAGTGAAAACAATCACGAATGACCAAATTGAAGTGACTAATGCTACTGAGCTGGTTCAGAGTTCCTCAACAGGTAGAATATGCGACAGTCCTCACCAAATCCTTGATGGAGAAAACTGCACACTGATAGATGCTCTATTGGGAGACCCACATTGTGATGGCTTCCAAAATAAGGAATGGGACCTTTTTGTTGAACGCAGCAAAGCCTACAGCAACTGTTACCCTTATGATGTGCCGGATTATGCCTCCCTTAGGTCACTAGTTGCCTCATCCGGCACCCTGGAGTTTAACAATGAAAGCTTCAATTGGACTGGAGTCGCTCAGAATGGAACAAGCTCTTCTTGCAAAAGGAGATCTATTAAAAGTTTCTTTAGTAGATTGAATTGGTTGCACCAATTAAAATACAGATATCCAGCACTGAACGTGACTATGCCAAACAATGACAAATTTGACAAATTGTACATTTGGGGGGTTCACCACCCGAGTACGGACAGTGACCAAACCAGCCTATATACCCAAGCATCAGGGAGAGTCACAGTCTCTACCAAAAGAAGCCAACAAACTGTAATCCCGAATATCGGATCCAGACCCTGGGTAAGGGGTATCTCCAGCAGAATAAGCATCTATTGGACAATAGTAAAACCGGGAGACATACTTTTGATTAACAGCACAGGGAATCTAATTGCTCCTCGGGGTTACTTCAAAATACGAAGTGGGAAAAGCTCAATAATGAGGTCAGATGCACCCATTGGCAAATGCAATTCTGAATGCATCACTCCAAATGGAAGCATTCCCAATGACAAACCATTTCAAAATGTAAACAGGATCACATATGGGGCCTGTCCCAGATATGTTAAGCAAAACACTCTGAAATTGGCAACAGGGATGCGGAATGTACCAGAGAAACAAACTAGA

A/SouthAustralia/59/2000

CAAAAACTTCCCGGAAATGACAACAGCACGGCAACGCTGTGCCTGGGGCACCATGCAGTGCCAAACGGAACGCTAGTGAAAACAATCACGAATGACCAAATTGAAGTGACTAATGCTACTGAGCTGGTTCAGAGTTCCTCAACAGGTGGAATATGCGACAGTCCTCACCAAATCCTTGATGGAGAAAACTGCACACTAATAGATGCTCTATTGGGAGACCCTCATTGTGATGGCTTCCAAAATAAGGAATGGGACCTTTTTGTTGAACGCAGCAAAGCCTACAGCAACTGTTACCCTTATGATGTGCCGGATTATGCCTCCCTTAGGTCACTAGTTGCCTCATCCGGCACACTGGAGTTTAACAATGAAAGCTTCAATTGGACTGGAGTCGCTCAGAATGGAACAAGCTCTGCTTGCAAAAGGAGATCTAATAAAAGTTTCTTTAGTAGATTGAATTGGTTGCACCAATTAAAATACAAATACCCAGCACTGAACGTGACTATGCCAAACAATGAAAAATTTGACAAATTGTACATTTGGGGGGTTCACCACCCGAGTACGGACAGTGACCAAATCAGCCTATATGCTCAAGCACCAGGGAGAGTCACAGTCTCTACCAAAAGAAGCCAACAAACTGTAATCCCGAATATCGGATCTAGACCCTGGGTAAGGGGTGTCTCCAGCAGAATAAGCATCTATTGGACAATAGTAAAACCGGGAGACATACTTTTGATTAACTGCACAGGGAATCTAATTGCTCCTCGGGGTTACTTCAAAATACGAAGTGGGAAAAGCTCAATAATGAGGTCAGATGCATCCATTGGCAAATGCAATTCTGAATGCATCACTCCAAATGGAAGCATTCCCAATGACAAACCATTTCAAAATGTAAACAGGATCACATATGGGGCCTGTCCCAGATATGTTAAGCAAAACACTCTGAAATTGGCAACAGGGATGCGGAATGTACCAGAGAAACAAACTAGA

A/Netherlands/126/01

CAAAAACTTCCCGGAAATGACAACAGCACGGCAACGCTGTGCCTGGGACACCATGCAGTGCCAAACGGAACGCTAGTGAAAACAATCACGAATGACCAAATTGAAGTGACTAATGCTACTGAGCTGGTTCAGAGTTCCTCAACAGGTGGAATATGCGACAGTCCTCACCAAATCCTTGATGGAGAAAACTGCACACTAATAGATGCTCTATTGGGAGACCCTCATTGTGATGGCTTCCAAAATAAGGAATGGGACCTTTTTGTTGAACGCAGCAAAGCCTACAGCAACTGTTACCCTTATGATGTGCCGGATTATGCCTCCCTTAGGTCACTAGTTGCCTCATCCGGCACACTGGAGTTTAACAATGAAAGCTTCAATTGGACTGGAGTCGCTCAGAATGGAACAAGCTCTGCTTGCAAAAGGAGATCTAATAAAAGTTTCTTTAGTAGATTGAATTGGTTGCACCAATTAAAATACAAATACCCAGCACTGAACGTGACTATGCCAAACAATGAAAAATTTGACAAATTGTACATTTGGGGGGTTCACCACCCGAGTACGGACAGTGACCAAATCAGCTTATATGCTCAAGCACCAGGGAGAGTCACAGTCTCTACCAAAAGAAGCCAACAAACTGTAATCCCGAATATCGGATCTAGACCCTGGGTAAGGGGTGTCTCCAGCAGAATAAGCATCTATTGGACAATAGTAAAACCGGGAGACATACTTTTGATTAACTGCACAGGGAATCTAATTGCTCCTCGGGGTTACTTCAAAATACGAAGTGGGAAAAGCTCAATAATGAGGTCAGATGCATCCATTGGCAAATGCAATTCTGAATGCATCACTCCAAATGGAAGCATTCCCAATGACAAACCATTTCAAAATGTAAACAGGATCACATATGGGGCCTGTCCCAGATATGTTAAGCAAAACACTCTGAAATTGGCAACAGGGATGCGGAATGTACCAGAGAAACAAACTAGA

A/Denmark/13/2002

CAAAAACTTCCCGGAAATGACAACAGCACGGCAACGCTGTGCCTGGGGCACCATGCAGTGCCAAACGGAACGCTAGTGAAAACAATCACGAATGACCAAATTGAAGTAACTAATGCTACTGAGCTGGTTCAGAGTTCCTCAACAGGTAGAATATGCGACAGTCCTCACCAAATCCTTGATGGAGAAAACTGCACACTAATAGATGCTCTATTGGGAGACCCTCATTGTGATGGCTTCCAAAATAAGGAATGGGACCTTTTTGTTGAACGCAGCAAAGCCTACAGCAACTGTTACCCTTATGATGTGCCGGATTATGTCTCCCTTAGGTCACTAGTTGCCTCATCAGGCACGCTGGAGTTTAACAATGAAAGCTTCAATTGGACTGGAGTCGCTCAGAATGGAACAAGCTCTGCTTGCAAAAGGAGATCTGATAAAAGTTTCTTTAGTAGATTGAATTGGTTGCACCAATTAAAATACAAATATCCAGCACTGAACGTGACTATGCCAAACAATGAAAAATTTGACAAATTGTACATTTGGGGGGTTCACCACCCGGGTACAGACAGTGACCAAATCAGCCTATATGCTCAAGCATCAGGGAGAGTCACAGTCTCTACCAAAAGAAGCCAACAAACTGTAATCCCGAATATCGGATCTAGACCCTGGGTAAGGGGTGTCTCCAGCAGAATAAGCATCTATTGGACAATAGTAAAACCGGGAGACATACTTTTGATTAACAGCACAGGGAATCTAATTGCTCCTCGGGGTTACTTCAAAATACGAAGTGGGAAAAGCTCAATAATGAGGTCAGATGCACCCATTGGCAAATGCAATTCTGAATGCATCACTCCAAATGGAAGCATTCCCAATGGCAAACCATTTCAAAATGTAAACAGGATCACATATGGGGCCTGTCCCAGATATGTTAAGCAAAACACTCTGAAATTGGCAACAGGGATGCGGAATGTGCCAGAGAAACAAACTAGA

A/Queensland/35/2003

CAAAAACTTCCCGGAAATGACAACAGCACGGCAACGCTGTGCCTTGGGCACCATGCAGTACCAAACGGAACGATAGTGAAAACAATCACAAATGACCAAATTGAAGTTACTAATGCTACTGAGCTGGTTCAGAGTTCCTCAACAGGTGGAATATGCGACAGTCCTCATCAGATCCTTGATGGAGAAAACTGCACACTAATAGATGCTCTATTGGGAGACCCTCAGTGTGATGGCTTCCAAAATAAGAAATGGGACCTTTTTGTTGAACGCAGCAAAGCCTACAGCAACTGTTACCCTTATGATGTGCCGGATCATGCCTCCCTTAGGTCACTAGTTGCCTCATCCGGCACACTGGAGTTTAACAATGAAAGCTTCGATTGGACTGGAGTCACTCAGAATGGAACAAGCTCTGCTTGCAAAAGGAGATCTAATAAAAGTTTCTTTAGTAGATTGAATTGGTTGACCCACTTAAAATACAAATACCCAGCATTGAACGTGACTATGCCAAACAATGAAAAATTTGACAAATTGTACATTTGGGGGGTTCACCACCCGGGTACGGACAGTGACCAAATCAGCCTATATGCTCAAGCATCAGGAAGAATCACAGTCTCTACCAAAAGAAGCCAACAAACTGTAATCCCGAATATCGGATCTAGACCCAGGGTAAGGGATGTCTCCAGCCGAATAAGCATCTATTGGACAATAGTAAAACCGGGAGACATACTTTTGATTAACAGCACAGGGAATCTAATTGCTCCTCGGGGTTACTTCAAAATACGAAGTGGGAAAAGCTCAATAATGAGATCAGATGCACCCATTGGCAAATGCAATTCTGAATGCATCACTCCAAATGGAAGCATTCCCAATGACAAACCATTTCAAAATGTAAACAGGATCACATATGGGGCCTGTCCCAGATATGTTAAGCAAAACACTCTGAAATTGGCAACAGGGATGCGAAATGTACCAGAGAAACAAACTAGA

A/Denmark/12/2004

CAAAAACTTCCCGGAAATGACAACAGCACGGCAACGCTGTGCCTTGGGCACCATGCAGTACCAAACGGAACGATAGTGAAAACAATCACGAATGACCAAATTGAAGTTACTAATGCTACTGAGCTGGTTCAGATTTCCTCAACAGGTGAAATATGCGACAGTCCCCATCAGATCCTTGATGGAGAAAACTGCACACTAATAGATGCTCTATTGGGAGACCCTCAGTGTGATGGCTTCCAAAATACGAAATGGGACCTTTTTGTTGAACGCAGCAAAGCCTACAGCAACTGTTACCCTTATGATGTGCCGGATTATGCCTCCCTTAGGTCACTAGTTGCCTCATCCGGCACACTGGAGTTTAACAATGAAAGCTTCGATTGGACTGGAGTCACTCAGAATGGAACAAGCTCTGCTTGCAAAAGGAGATCTGATAAAAGTTTCTTTAGTAGATTGAATTGGTTGACCCACTTAAAATACAAATACCCAGCATTGAACGTAACTATGCCAAACAATGAAAAATTTGACAAATTGTACATTTGGGGGGTTCACCACCCGGGGACGGACAGTGACCAAATCAGCCTATATGCTCAAGCATCAGGAAGAATCACAGTCTCTACCAAAAGAAGCCAACAAACTGTAATCCCGAATATCGGATCTAGACCCAGGGTAAGGGATGTCTCCAGCCGAATAAGCATCTATTGGACAATAGTAAAACCGGGAGACATACTTTTGATTAACAGCACAGGGAATCTAATTGCTCCTCGGGGTTACTTCAAAATACGAAGTGGGAAAAGCTCAATAATGAGATCAGATGCACCCATTGGCAAATGCAATTCTGAATGCATCACTCCAAATGGAAGCATTCCCAATGACAAACCATTTCAAAATGTAAACAGGATCACATATGGGGCCTGTCCCAGATATGTTAAGCAAAACACTCTGAAATTGGCAACAGGGATGCGAAATGTACCAGAGAAACAAACTAGA

A/Cambodia/7/2005

CAAAAACTTCCCGGAAATGACAACAGCACGGCAACGCTGTGCCTTGGGCACCATGCAGTACCAAACGGAACGATAGTGAAAACAATCACGAATGACCAAATTGAAGTTACTAATGCTACTGAGCTGGTTCAGAGTTCCTCAACAGGTGGAATATGCGACAGTCCTCATCAGATCCTTGATGGAGAAAACTGCACACTAATAGATGCTCTATTGGGAGACCCTCAGTGTGATGGCTTCCAAAATAAGAAATGGGACCTTTTTGTTGAACGCAGCAAAGCCTACAGCAACTGTTACCCTTATGATGTGCCGGATTATGCCTCCCTTAGGTCACTAGTTGCCTCATCCGGCACACTGGAGTTTAACAATGAAAGCTTCAATTGGACTGGAGTCACTCAAAATGGAACAAGCTCTGCTTGCAAAAGGAGATCTAATAACAGTTTCTTTAGTAGATTGAATTGGTTGACCCACTTAAAATTCAAATACCCAGCATTGAACGTGACTATGCCAAACAATGAAAAATTTGACAAATTGTACATTTGGGGGGTCCACCACCCGGGTACGGACAATGACCAAATCTTCTTGTATGCTCAAGCATCAGGAAGAATCACAGTCTCTACCAAAAGAAGCCAACAAACTGTAATCCCGAATATCGGGTCTAGACCCAGAGTAAGGAATATCCCCAGCAGAATAAGCATCTATTGGACAATAGTAAAACCGGGAGACATACTTTTGATTAACAGCACAGGGAATCTAATTGCTCCTAGGGGTTACTTCAAAATACGAAGTGGGAAAAGCTCAATAATGAGATCAGATGCACCCATTGGCAAATGCAATTCTGAATGCATCACTCCAAATGGAAGCATTCCCAATGACAAACCATTTCAAAATGTAAACAGGATCACATATGGGGCCTGTCCCAGATATGTTAAGCAAAACACTCTGAAATTGGCAACAGGGATGCGAAATGTACCAGAGAAACAAACTAGA

A/Wisconsin/45/2006

CAAAAACTTCCCGGAAATGACAACAGCACGGCAACGCTGTGCCTTGGGCACCATGCAGTACCAAACGGAACGATAGTGAAAACAATCACGAATGACCAAATTGAAGTTACTAATGCTACTGAACTGGTTCAGAGTTCCTCAACAGGTGAAATATGCGACAGTCCTCATCAGATCCTTGATGGAGAAAACTGCACACTAATAGATGCCCTATTGGGAGACCCTCAGTGTGATGGCTTCCAAAATAAGAAATGGGACCTTTTTGTTGAACGCAGCAAAGCCTACAGCAACTGTTACCCTTATGATGTGCCGGATTATGCCTCCCTTAGGTCACTAGTTGCCTCATCCGGCACACTGGAGTTTAACAATGAAAACTTCAATTGGACTGGAGTCACTCAGAATGGAACAAGCTCTGCTTGCAAAAGGAGATCTAATAACAGTTTCTTTAGTAGACTGAATTGGTTGACCCACTCAAAATTCAAATACCCAGCATTGAACGTGACTATGCCAAACAATGAAAAATTTGACAAATTGTACATTTGGGGGGTTCACCACCCGGGTACGGACAATGACCAAATCTTCCTGTATGCTCAAGCATCAGGAAGAATCACAGTCTCTACCAAAAGAAGCCAACAAACTGTAATCCCGAACATCGGATCTAGACCTAGAGTAAGGAATATCCCCAGCAGAATAAGCATCTATTGGACAATAGTAAAACCGGGAGACATACTTTTGATTAACAGCACAGGGAATCTAATTGCTCCTAGGGGTTACTTCAAAATACGAAGTGGGAAAAGCTCAATAATGAGATCAGATGCACCCATTGGCAAATGCAATTCTGAATGCATCACTCCAAATGGAAGCATTCCCAATGACAAACCATTTCAAAATGTAAACAGAATCACATATGGGGCCTGTCCCAGATATGTTAAGCAAAACACTCTGAAATTGGCAACAGGGATGAGAAATGTACCAGAGAAACAAACTAGA

A/Germany/AF1009/2007

CAAAAACTTCCCGGAATTGACAACAGCACGGCAACGCTGTGCCTTGGGCACCATGCAGTACCAAACGGAACGATAGTGAAAACAATCACGAATGACCAAATTGAAGTTACTAATGCTACTGAGCTGGTTCAGAGTTCCTCAACAGGTGGAATATGCGACAGTCCTCATCAGATCCTTGATGGAGAAAACTGCACACTAATAGATGCTCTATTGGGAGACCCTCAATGTGATGGCTTCCAAAATAAGAAATGGGACCTTTTTGTTGAACGCAGCAAAGCCTACAGCAACTGTTACCCTTATGATGTGCCGGATTATGCCTCCCTTAGGTCACTAGTTGCCTCATCCGGCACACTGGAGTTTAACAATGAAAGCTTCAATTGGGCTGGAGTCACTCAAAATGGAACAAGCTCTGCTTGCAAAAGGGGATCTAATAACAGTTTCTTTAGTAGATTGAATTGGTTGACCCACTCAAAATTCAAATACCCAGCATTGAACGTGACTATGCCAAACAATGAAGAATTTGACAAATTGTACATTTGGGGGGTTCACCACCCGGGTACGGACAATGACCAAATCTTCCTGTATGCTCAAGCATCAGGAAGAATCACAGTCTCTACCAAAAGAAGCCAACAAACTGTAATCCCGAATATCGGATCTAGACCCAGAGTAAGGAATATCCCCAGCAGAATAAGCATCTATTGGACAATAGTAAAACCGGGAGACATACTTTTGATTAACAGCACAGGGAATCTAATTGCTCCTAGGGGTTACTTCAAAATACGAAGTGGGAAAAGCTCAATAATGAGATCAGATGCACCCATTGGCAAATGCAATTCTGAATGCATCACTCCAAATGGAAGCATTCCCAATGACAAACCATTTCAAAATGTAAACAGGATCACATACGGGGCCTGTCCCAGATATGTTAAGCAAAACACTCTGAAATTGGCAACAGGGATGCGAAATGTACCAGAGAAACAAACTAGA

A/Guatemala/AF1847/2008

CAAAAATTTCCTGGAAATGACAACAGCACGGCAACGCTGTGCCTTGGGCACCATGCAGTACCAAACGGAACGATAGTGAAAACAATCACGAATGACCAAATTGAAGTTACTAATGCTACTGAGCTGGTTCAGAGTTCCTCAACAGGTGAAATATGCGACAGTCCTCATCAGATCCTTGATGGAGAAAACTGCACACTAATAGATGCTCTATTGGGAGACCCTCAGTGTGATGGCTTCCAAAATAAGAATTGGGACCTTTTTGTTGAACGCAGCAAAGCCTACAGCAACTGTTACCCTTATGATGTGCCGGATTATGCCTCCCTTAGGTCACTAGTTGCCTCATCCGGCACACTGGAGTTTAACAATGAAAGCTTCAATTGGACTGGAGTCACTCAAAACGGAACAAGCTCTGCTTGCATAAGGAGATCTAATAACAGTTTCTTTAGTAGATTGAATTGGTTGACCCACTCAAAATTCAAATACCCAGCATTGAACGTGACTATGCCAAACAATGAAAATTTTGACAAATTGTACATTTGGGGGGTTCACCACCCGGGTACGGACAATGACCAAATCTTCCTGTATGCTCAAGCATCAGGAAGAATCACAGTCTCTACCAAAAGAAGCCAACAGACTGTAATCCCGAATATCGGATCTAGACCCAGAGTAAGGAATATCCCTAGCAGAATAAGCATCTATTGGACAATAGTAAAACCGGGAGACATACTTTTGATTAACAGCACAGGGAATCTAATTGCTCCTAGGGGTTACTTCAAAATACGAAGTGGGAAAAGCTCAATAATGAGATCAGATGCACCCATTGGCAAATGCAATTCTGAATGCATCACTCCAAATGGAAGCATTCCCAATGACAAACCATTCCAAAATGTAAACAGGATCACATACGGGGCCTGTCCCAGATATGTTAAGCAAAACACTCTGAAATTGGCAACAGGGATGCGAAATGTACCAGAGAAACAAACTAGA

A/Qingdao/1046/2009

CAAAAACTTCCTGGAAATGACAACAGCACGGCAACGCTGTGCCTTGGGCACCATGCAGTACCAAACGGAACGATAGTGAAAACAATCACTAATGACCAAATTGAAGTTACTAATGCTACTGAGCTGGTTCAGAGTTCCTCAACAGGTGAAATATGCGACAGTCCTCATCAGATCCTTGATGGAAAAAACTGCACACTAATAGATGCTCTATTGGGAGACCCTCAGTGTGATGGCTTCCAAAATAAGAAATGGGACCTTTTTGTTGAACGCAGCAAAGCCTACAGCAACTGTTACCCTTATGATGTGCCGGATTACGCCTCCCTTAGGTCACTAGTTGCCTCATCCGGCACACTGGAGTTTAACAATGAAAGCTTCAATTGGACTGGAGTCACTCAAAACGGAACAAGCTCTGCTTGCATAAGGAGATCTAAAAACAGTTTCTTTAGTAAATTGAATTGGTTGACCCACTTAAACTTCAAATACCCAGCATTGAACGTGACTATGCCAAACAATGAACAATTTGACAAATTGTACATTTGGGGGGTTCACCACCCGGGTACGGACAGAGACCAAATCTTCCTGTATGCTCAAGCATCAGGAAGAATCACAGTCTCTACCAAAAGAAGCCAACAAACTGTAATCCCGAATATCGGATCTAGACCCAGAGTAAGGAATATCCCTAGCAGAATAAGCATCTATTGGACAATAGTAAAACCGGGAGACATACTTTTAATTAACAGCACAGGGAATCTAATTGCTCCTAGGGGTTACTTCAAAATACGAAGTGGGAAAAGCTCAATAATGAGATCAGATACACCCATTGGCAAAAGCAATTCTGAATGCATCACTCCAAATGGAAGCATTCCCAATGACAAACCATTCCAAAATGTAAACAGGATCACATACGGGGCCTGTCCCAGATATGTTAAGCAAAACACTCTGAAATTGGCAACAGGGATGCGAAATGTACCAGAGAAACAAACTAGA

A/Italy/AF2157/2010

CAAAAACTTCCTGGAAATGACAACAGCACGGCAACGCTGTGCCTTGGGCACCATGCAGTACCAAACGGAACGATAGTGAAAACAATCACGAATGACCAAATTGAAGTTACTAATGCTACTGAGCTGGTTCAGAATTCCTCAACAGGTGAAATATGCGACAGTCCTCATCAGATCCTTGATGGAGAAAACTGCACACTAATAGATGCTCTATTGGGGGACCCTCAGTGTGATGGCTTCCAAAATAAGAAATGGGACCTTTTTGTTGAACGAAGCAAAGCCTACAGCAACTGTTACCCTTATGATGTGCCGGATTATGCCTCCCTTAGGTCACTAGTTGCCTCATCCGGCACACTGGAGTTTAACAATGAAAGCTTCAATTGGACTGGAGTCACTCAAAACGGAACAAGCTCTGCTTGCATAAGGAGATCTAATAATAGTTTCTTTAGTAGATTGAATTGGTTGACCCACTTAAACTTCAAATACCCAGCATTGAACGTGACTATGCCAAACAATGAACAATTTGACAAATTGTACATTTGGGGGGTTCACCACCCGGGTACGGACAAGGACCAAATCTTCCTGTATGCTCAAGCATCAGGAAGAATCACAGTATCTACCAAAAGAAGCCAACAAGCTGTAATCCCGAATATCGGATCTAGACCCAGAGTAAGGAATATCCCTAGCAGAATAAGCATCTATTGGACAATAGTAAAACCGGGAGACATACTTTTGATTAACAGCACAGGGAATCTAATTGCTCCTAGGGGTTACTTCAAAATACGAAGTGGGAAAAGCTCAATAATGAGATCAGATGCACCCATTGGCAAATGCAATTCTGAATGCATCACTCCAAATGGAAGCATTCCCAATGACAAACCATTCCAAAATGTAAACAGGATCACATACGGGGCCTGTCCCAGATATGTTAAGCAAAACACTCTGAAATTGGCAACAGGAATGCGAAATGTACCAGAGAAACAAACTAGA

A/Aichi/2/68

CAAGACCTTCCAGGAAATGACAACAGCACAGCAACGCTGTGCCTGGGACATCATGCGGTGCCAAACGGAACACTAGTGAAAACAATCACAGATGATCAGATTGAAGTGACTAATGCTACTGAGCTAGTTCAGAGCTCCTCAACGGGGAAAATATGCAACAATCCTCATCGAATCCTTGATGGAATAGACTGCACACTGATAGATGCTCTATTGGGGGACCCTCATTGTGATGTTTTTCAAAATGAGACATGGGACCTTTTCGTTGAACGCAGCAAAGCTTTCAGCAACTGTTACCCTTATGATGTGCCAGATTATGCCTCCCTTAGGTCACTAGTTGCCTCGTCAGGCACTCTGGAGTTTATCACTGAGGGTTTCACTTGGACTGGGGTCACTCAGAATGGGGGAAGCAATGCTTGCAAAAGGGGACCTGGTAGCGGTTTTTTCAGTAGACTGAACTGGTTGACCAAATCAGGAAGCACATATCCAGTGCTGAACGTGACTATGCCAAACAATGACAATTTTGACAAACTATACATTTGGGGGATTCACCACCCGAGCACGAACCAAGAACAAACCAGCCTGTATGTTCAAGCATCAGGGAGAGTCACAGTCTCTACCAGGAGAAGCCAGCAAACTATAATCCCGAATATCGGGTCCAGACCCTGGGTAAGGGGTCTGTCTAGTAGAATAAGCATCTATTGGACAATAGTTAAGCCGGGAGACGTACTGGTAATTAATAGTAATGGGAACCTAATCGCTCCTCGGGGTTATTTCAAAATGCGCACTGGGAAAAGCTCAATAATGAGGTCAGATGCACCTATTGATACCTGTATTTCTGAATGCATCACTCCAAATGGAAGCATTCCCAATGACAAGCCCTTTCAAAACGTAAACAAGATCACATATGGAGCATGCCCCAAGTATGTTAAGCAAAACACCCTGAAGTTGGCAACAGGGATGCGGAATGTACCAGAGAAACAAACTAGA

A/HongKong/3/69

CAAGACCTTCCAGGAAATGACAACAGCACAGCAACGCTGTGCCTGGGACATCATGCGGTGCCAAACGGAACACTAGTGAAAACAATCACAGATGATCAGATTGAAGTGACTAATGCTACTGAGCTAGTTCAGAGCTCCTCAACGGGGAAAATATGCAACAATCCTCATCGAATCCTTGATGGACTAGACTGCACACTGATAGATGCTCTATTGGGGGACCCTCATTGTGATGTTTTTCAAAATGAGACATGGGACCTTTTCGTTGAACGCAGCAAAGCTTTCAGCAACTGTTACCCTTATGATGTGCCAGATTATGCATCCCTTAGGTCACTAGTTGCCTCGTCAGGCACTCTGGAGTTTATCACTGAGGGTTTCACTTGGACTGGGGTCACTCAGAATGGGGGAAGCAATGCTTGCAAAAGGGGACCTGGTAGCGGTTTTTTCAGTAGACTGAACTGGTTGACCAAATCAGGAAGCACATATCCAGTGCTGAACGTGACTATGCCAAACAATGACAATTTTGACAAACTATACATTTGGGGGGTTCATCACCCGAGCACGAACCAAGAACAAACCAGCCTGTATGTTCAAGCATCAGGGAGAGTCACAGTTTCTACCAGGAGAAGCCAGCAGACTATAATCCCGAATATCGGGTCCAGACCCTGGGTAAGGGGTCTGTCTAGTAGAATAAGCATCTATTGGACAATAGTTAAGCCGGGAGACGTACTGGTAATTAATAGTAATGGGAACCTAATCGCTCCTCGGGGTTATTTCAAAATGCGCACTGGGAAAAGCTCAATAATGAGGTCAGATGCAACTATTGATACCTGTATTTCTGAATGCATCACTCCAAATGGAAGCATTCCCAATGACAAACCCTTTCAAAACGTAAACAAGATCACATATGGAGCATGCCCCAAGTATGTTAAGCAAAACACCCTGAAGTTGGCAACAGGGATGCGGAATGTACCAGAGAAACAAACTAGA

A/Bilthoven/2668/70

CAAGACCTTCCAGGAAATGACAACAGTACAGCAACGCTGTGCCTGGGACATCATGCGGTGCCAAACGGAACACTAGTGAAAACAATCACAAATGATCAGATTGAAGTGACTAATGCTACTGAGCTAGTTCAGAGCTCCTCAACGGGGAAAATATGCAACAATCCTCATCGAATCCTTGATGGAATAAACTGCACACTGATAGACGCTCTATTGGGGGACCCTCATTGTGATGTTTTCCAAGATGAGACATGGGACCTTTTCGTTGAACGCAGCAAAGCTTTCAGCAACTGTTACCCTTATGATGTGCCAGATTATGCCTCCCTTAGGTCACTAATTGCCTCGTCAGGCACTCTGGAGTTTATCACTGAGGGTTTCACTTGGACTGGGGTCACTCAGAATGGGGGAAGCAATGCTTGCAAAAGGGGACCTGGTAGCGGTTTTTTCAGTAGACTGAACTGGTTGACCAAATCAGGAAGCACATATCCAGTGCTGAACGTGACTATGCCAAACAATGACAATTTTGACAAACTATACATTTGGGGGGTTCACCACCCGAGCACGAACCAAGAACAAACCAGCCTGTATGTTCAAGCATCAGGGAGAGTCACAGTCTCTACCAGGAGAAGCCAGCAAACTATAATCCCGAATATCGGGTCCAGACCCTGGGTAAGGGGTCTGTCTAGTAGAATAAGCATCTATTGGACAATAGTTAAACCGGGAGACGTACTGGTAATTAATAGTAATGGGAACCTAATCGCTCCTCGGGGTTATTTCAAAATGCGCACTGGGAAAAGCTCAATAATGAGGTCAGATGCACCTATTGATACCTGTATTTCTGAATGCATCACTCCAAATGGAAGCATTCCCAATGACAAGCCCTTTCAAAACGTAAACAAGATCACATATGGAGCATGCCCCAAGTATGTTAAGCAAAACACCCTGAAATTGGCAACAGGGATGCGGAATGTACCAGAGAAACAAACTAGA

A/Bilthoven/21438/71

CAAGACCTTCCAGGAAATGACAAAAGCACAGCAACGCTGTGCCTGGGACATCATGCGGTGCCAAACGGAACACTAGTGAAAACAATCACAAATGATCAGATTGAAGTGACTAATGCTACTGAGCTGGTTCAGAGCTCCTCAACGGGGAAAATATGCAACAATCCTCATCGAATCCTTGATGGAATAGACTGCACACTGATAGATGCTCTATTGGGGGACCCTCATTGTGATGGCTTTCAAAATGAGACATGGGACCTTTTCGTTGAACGCAGCAAAGCTTTCAGCAACTGTTACCCTTATGATGTGCCAGATTATGCCTCCCTTAGGTCACTAGTTGCCTCGTCAGGCACTCTGGAGTTTATCACTGAGGGTTTCACTTGGACTGGGGTCACTCAGAATGGGGGAAGCAGTGCTTGCAAAAGGGGACCTGGTAGCGGTTTTTTCAGTAGACTGAACTGGTTGACCAAATCAGGAAGCACATATCCAGTGCTGAACGTGACTATGCCAAACAATGACAATTTTGACAAACTATACATTTGGGGGGTTCACCACCCGAGCACGAACCAAGAACAAACCAGCCTGTATGTACAAGCATCAGGGAGAGTCACAGTCTCTACCAGGAGAAGCCAGCAAACTATAATCCCGAATATCGGGTCTAGACCCTGGGTAAGGGGTCTGTCTAGTAGAATAAGCATCTATTGGACAATAGTTAAACCGGGAGACATACTGATAATTAATAGTAATGGGAACCTAATTGCTCCTCGGGGTTATTTCAAAATGCGCACTGGGAAAAGCTCAATAATGAGGTCAGATGCACCTATTGATACCTGTATTTCTGAATGCATCACTCCAAATGGAAGCATTCCCAATGACAAGCCCTTTCAAAATGTAAACAAGATCACATATGGGGCATGCCCCAAGTATGTTAAGCAAAACACCCTGAAGTTGGCAACAGGGATGCGGAATGTACCAGAGAAACAAACTAGA

A/Bilthoven/6022/72

CAAGACCTTCCAGGAAATGACAAAAGCACAGCAACGCTGTGCCTGGGACACCATGCAGTGCCAAACGGAACACTAGTGAAAACAATCACAAATGATCAGATTGAAGTGACTAATGCTACTGAGCTGGTTCAGAGCTCCTCAACGGGGAAAATATGCAACAATCCTCATCGAATCCTTGATGGAATAGACTGCACACTGATAGATGCTCTATTGGGGGACCCTCATTGTGATGGCTTTCAAAATGAGACATGGGACCTTTTCGTTGAACGCAGCAAAGCTTTCAGCAACTGTTACCCTTATGATGTGCCAGATTATGCCTCCCTTAGGTCACTAGTTGCCTCGTCAGGCACTCTGGAGTTTATCACTGAGGGTTTCACTTGGACTGGGGTCACTCAGAATGGGGGAAGCAATGCTTGCAAAAGGGGACCTGGTAGCGGTTTTTTCAGTAGGCTGAACTGGTTGACCAAATCAGGAAGCACATATCCAGTGCTGAACGTGACTATGCCAAACAATGACAATTTTGACAAACTATACATTTGGGGGGTTCACCACCCGAGCACGAACCAAGAACAAACCAGCCTGTATGTACAAGCATCAGGGAGAGTCACAGTCTCCACCAAGAGAAGCCAGCAAACTATAATCCCGAATATCGGGTCTAGACCCTGGGTAAGGGGTCCGTCTAGCAGAATAAGCATCTATTGGACAACAGTTAAACCGGGAGACATACTGATAATCAATAGTAATGGGAACCTAATTGCTCCTCGGGGTTATTTCAAAATGCGAACTGGGAAAAGCTCAATAATGAGGTCAGATGCACCTATTGGTGCCTGTATTTCTGAATGCATCACTCCAAATGGAAGCATTCCCAATGACAAGCCCTTTCAAAATGTAAACAAGATCACATATGGGGCATGCCCCAAGTATGTTAAGCAAAACACCCTGAAGTTGGCAACAGGGATGCGGAATGTACCAGAGAAACAAACTAGA

A/Memphis/3/73

CAAGATTTTCCAGGAAATGACAACAGCACAGCAACGCTGTGCCTGGGACATCATGCGGTGCCAAACGGAACACTAGTGAAAACAATCACAAATGATCAGATTGAAGTGACTAATGCTACTGAGCTGGTTCAGAGTTCCTCAACGGGGAAAATATGCAACAATCCTCATCGAATCCTTGATGGAATAGACTGCACACTGATAGATGCTCTATTGGGGGACCCTCATTGTGATGGCTTTCAAAATGAGACATGGGACCTTTTCGTTGAACGCAGCAAAGCTTTCAGCAACTGTTACCCTTATGATGTGCCAGATTATGCCTCCCTTAGGTCACTAGTTGCCTCGTCAGGCACTCTGGAGTTTATCAATGAAGGCTTCACTTGGACTGGGGTCACTCAGAATGGGGGAAGCAATGCTTGCAAAAGGGGACCTGATAGCGGTTTTTTCAGTAGACTGAACTGGTTGTACAAATCAGGAAGCACATATCCAGTGCTGAACGTGACTATGCCAAACAATGACAATTTTGACAAACTATACATTTGGGGGGTTCACCACCCGAGCACGGACCAAGAACAAACCAGCCTATATGTTCAAGCATCAGGGAGAGTCACAGTCTCTACCAAGAGAAGCCAGCAAACTATAATCCCGAATATCGGGTCTAGACCCTGGGTAAGGGGTCTGTCTAGTAGAATAAGCATCTATTGGACAATAGTTAAACCAGGAGACATACTGGTAATTAATAGTAATGGGAACCTAATTGCTCCTCGGGGTTATTTCAAAATGCGCACTGGGAAAAGCTCAATAATGAGGTCAGATGCACCTATTGGCACCTGCATTTCTGAATGCATCACTCCAAATGGAAGCATTCCCAATGACAAGCCCTTTCAAAACGTAAACAAGATCACATATGGGGCATGTCCCAAGTATGTTAAGCAAAACACCCTGAAGTTGGCAACAGGGATGCGGAATGTACCAGAGAAACAAACTAGA

A/Memphis/103/74

CAAGACCTTCCAGGAAATGACAACAGCACAGCAACGCTGTGCCTGGGACATCATGCGGTGCCAAACGGAACGCTAGTGAAAACAATCACGAATGATCAGATTGAAGTGACTAATGCTACTGAGCTGGTTCAGAGCTCCTCAACGGGTAAAATATGCAACAATCCTCATCGAATCCTTGATGGAATAAACTGCACACTGATAGATGCTCTATTGGGGGACCCTCATTGTGATGGCTTTCAAAATGAGAAATGGGACCTTTTCGTTGAACGCAGCAAAGCTTTCAGCAACTGTTACCCTTATGATGTGCCAGACTATGCCTCCCTTAGGTCACTAGTTGCCTCGTCAGGCACTCTGGAGTTTATCAATGAAGGCTTCAATTGGACTGGGGTCACTCAGAATGGGGGAAGCAATGCTTGCAAAAGGGGACCTGATAGCGGTTTTTTCAGTAGACTGAACTGGTTGTACAAATCAGGAAGCACATATCCAGTGCTGAACGTGACTATGCCAAACAATGACAATTTTGACAAACTATACATTTGGGGGGTTCACCACCCGAGCACGGACCAAGAACAAACCAACCTATATGTTCAAGCATCAGGGAGAGTCACAGTCTCCACCAAGAGAAGCCAGCAAACTATAATCCCGAATGTTGGGTCTAGACCCTGGGTAAGGGGTCTGTCTAGTAGAATAAGCATCTATTGGACAATAGTTAAACCGGGAGACATACTGGTAATTAATAGTAATGGGAACCTAATTGCTCCTCGGGGTCACTTCAAAATGCGCACTGGGAAGAGCTCAATAATGAGGTCAGATGCACCTATTGGCACCTGCAGTTCTGAATGCATCACTCCAAATGGAAGCATTCCCAATGACAAGCCCTTTCAAAACGTAAACAAGATCACATATGGGGCATGTCCCAAGTATGTTAAGCAAAACACTCTGAAGTTGGCAACAGGGATGCGGAATGTACCAGAGAAACAAACTAGA

A/Victoria/3/75

CAAGACCTTCCAGGAAATGACAACAGCACAGCAACGCTGTGCCTGGGACATCATGCGGTGCCAAACGGAACGCTAGTGAAAACAATCACGAATGATCAGATTGAAGTGACTAATGCTACTGAGCTGGTTCAGAGTTCCTCAACGGGTAAAATATGCAACAATCCTCATCGAATCCTTGATGGAATAAACTGCACACTGATAGATGCTCTATTGGGGGACCCTCATTGTGATGGATTTCAAAATGAGAAATGGGACCTTTTCGTTGAACGCAGCAAAGCTTTCAGCAACTGTTACCCTTATGATGTGCCAGATTATGCCTCCCTTAGGTCACTAGTTGCCTCGTCAGGCACTCTGGAGTTTATCAATGAAGGCTTCAATTGGACTGGGGTCACTCAGAATGGGGGAAGCAGTGCTTGCAAAAGAGGACCTGATAGCGGTTTTTTCAGTAGACTGAACTGGTTGTACAAATCAGGAAGCACATATCCAGTGCAAAACGTGACCATGCCAAACAATGACAATTCTGACAAACTATACATTTGGGGGGTTCACCACCCGAGCACGGACAAAGAACAAACCAACCTATATGTTCAAGCATCAGGGAAAGTCACAGTCTCCACCAAGAGAAGCCAGCAAACTATAATCCCGAATGTCGGGTCTAGACCCTGGGTAAGGGGTCTGTCTAGTAGAATAAGCATCTATTGGACAATAGTTAAACCGGGAGACATACTGGTAATTAATAGTAATGGGAACCTAATTGCTCCTCGGGGTTACTTCAAAATGCGCACTGGGAAAAGCTCAATAATGAGGTCAGATGCACCTATTGGCACCTGCAGCTCTGAATGCATCACTCCAAATGGAAGCATTCCCAATGACAAGCCCTTTCAAAACGTAAACAAGATCACATATGGGGCATGTCCCAAGTATGTTAAGCAAAACACTCTGAAGTTGGCAACAGGGATGCGGAATGTACCAGAGAAACAAACTAGA

A/Bilthoven/1761/76

CAAGACCTTCCAGGAAATGACAACAGCACAGCAACTCTGTGCCTGGGACATCATGCGGTGCCAAACGGAACGCTAGTGAAAACAATCACGAATGATCAGATTGAAGTGACTAATGCTACTGAACTGGTTCAGAGTTCCTCAACGGGTAAAATATGCGACAATCCTCATCGAATCCTTGATGGAATAAACTGCACACTGATAGATGCTCTATTGGGGGACCCTCATTGTGATGGCTTTCAAAATGAGAAATGGGACCTTTTCGTTGAACGCAGCAAAGCTTTCAGCAACTGTTACCCTTATGATGTGCCAGATTATGCCTCCCTTAGGTCACTAGTTGCCTCGTCAGGCACTCTGGAGTTTATCAATGAAGGCTTCAATTGGACTGGGGTCACTCAGAATGGGGGAAGCAGTGCTTGCAAAAGGGGACCTGATAACGGTTTTTTCAGTAGACTGAACTGGTTGTACAAATCAGGAAGCACATATCCAGTGCAGAACGTGACCATGCCAAACAATGACAACTCTGACAAACTATACATTTGGGGGGTTCACCACCCGAGCACGGACAAAGAACAAACCGACCTATATGTTCAAGCATCAGGGAAAGTCACAGTCTCCACCAAGAGAAGCCAGCAAACTGTAATCCCGAATGTCGGGTCTAGACCCTGGGTAAGGGGTCTGTCTAGTAGAGTAAGCATCTATTGGACAATAGTTAAACCGGGAGACATACTGGTAATTAATAGTAATGGAAACCTAATTGCTCCTCGGGGTTACTTCAAAATGCGCACTGGGAAAAGCTCAATAATGAGGTCAGATGCACCTATTGGCACCTGCAGCTCTGAATGCATCACTCCAAATGGAAGCATTCCCAATGACAAGCCCTTTCAAAACGTAAACAAGATCACATATGGGGCATGTCCCAAGTATGTTAAGCAAAACACTCTGAAGTTGGCAACAGGGATGCGGAATGTACCAGAGAAACAAACTAGA

A/Texas/1/77

CAAAACCTTCCAGGAAATGACAACAGCACAGCAACGCTGTGCCTGGGACATCATGCGGTGCCAAACGGAACGCTAGTGAAAACAATCACGAATGATCAGATTGAAGTGACTAATGCTACTGAGCTGGTTCAGAGTTCCTCAACAGGTAGAATATGCGACAGTCCTCATCGAATCCTTGATGGGAAAAACTGCACACTGATAGATGCTCTATTGGGGGACCCTCATTGTGATGGCTTTCAAAATGAGAAATGGGACCTTTTTGTTGAACGCAGCAAAGCTTTCAGCAACTGTTACCCTTATGATGTGCCAGATTATGCCTCCCTTAGGTCACTAGTTGCCTCGTCAGGCACTCTGGAGTTTATCAATGAAGGCTTCAATTGGACTGGAGTCACTCAGAATGGGGGAAGCTATGCTTGCAAAAGGGGACCTGATAACGGTTTCTTCAGTAGACTGAACTGGTTGTACAAATCAGAAAGCACATATCCAGTGCTGAACGTGACTATGCCAAACAATGGCAATTTTGACAAACTGTACATTTGGGGAGTTCACCACCCGAGCACGGACAAAGAACAAACCAACCTATATGTTCAAGCATCAGGGAGAGTCACAGTCTCCACCAAGAGAAGCCAGCAAACTATAATCCCGAATGTCGGGTCTAGACCCTGGGTAAGGGGTCTGTCTAGTAGAATAAGCATCTATTGGACAATAGTAAAACCGGGAGACATACTGTTAATTAATAGTAATGGGAACCTAATTGCTCCTCGGGGTTACTTCAAAATACGCACTGGGAAAAGCTCAATAATGAGGTCAGATGCACCCATTGGCACCTGCAGCTCTGAATGCATCACTCCAAATGGAAGCATTCCCAATGACAAGCCCTTTCAAAACGTAAACAAGATCACATATGGGGCATGTCCCAAGTATGTTAAGCAAAACACTCTGAAGTTGGCAACAGGGATGCGGAATGTACCAGAGAAACAAACTAGA

A/Memphis/12/78

CAAAACCTTCCAGGAAATGACAACAGCACAGCAACGCTGTGCCTGGGACATCATGCAGTGCCAAACGGAACGCTAGTGAAAACAATCACGAATGATCAGATTGAAGTGACTAATGCTACTGAGCTGGTTCAGAGTTCCTCAACAGGTAGAATATGCGACAATCCTCATCGAATCCTTGATGGAAAAAACTGCACACTGATAGATGCTCTATTGGGGGACCCTCATTGTGATGGCTTTCAAAATGAGAAATGGGACCTTTTTGTTGAACGCAGCAAAGCTTTCAGCAACTGTTACCCTTATGATGTGCCAGATTATGCCTCCCTTAGGTCACTAGTTGCCTCGTCAGGCACCCTGGAGTTTTTCAATGAAGGCTTCAATTGGACTGGGGTCACTCAGAATGGGGGAAGCTATGCTTGTAAAAGGGGACCTGATAACAGTTTCTTCAGTAGACTGAACTGGTTGTACAAATCAGAAAGCACATATCCAGTGCTGAACGTAACTATGCCAAACAATGACAATTTTGACAAACTGTACATTTGGGGAGTTCACCACCCGAGCACGGACAAAGAACAAACCAACCTATATGTTCAAGCATCAGGGAGAGTCACAGTCTCCACCAAGAGAAGCCAGCAAACTATAATCCCGAATGTCGGGTCTAGACCCTGGGTAAGGGGTCTGTCTAGTAGAATAAGCATCTATTGGACAATAGTAAAACCGGGAGACGTACTGTTAATTAATAGTAATGGGAACCTAATTGCTCCTCGGGGTTACTTCAAAATACGCACTGGGAAAAGCTCAATAATGAGGTCAGATGCACCCATTGGCACCTGCAGTTCTGAATGTATCACTCCAAATGGAAGCATTCCCAATGACAGGCCCTTTCAAAACGTAAACAAGATCACATATGGGGCATGTCCCAAGTATGTTAAGCAAAACACTCTGAAGTTGGCAACAGGGATGCGGAATGTACCAGAGAAACAAACTAGA

A/Bangkok/1/1979

CAAAACCTTCCCGGAAATGACAACAGCACAGCAACGCTGTGCCTGGGACACCATGCAGTGCCAAACGGAACGCTAGTGAAAACAATCACGAATGATCAGATTGAAGTGACTAATGCTACTGAGCTGGTTCAGAGTTCCTCAACAGGTAGAATATGCGACAGTCCTCACCGAATCCTTGATGGGAAAAACTGCACACTGATAGATGCTCTATTGGGAGACCCTCATTGTGATGGCTTTCAAAATGAGAAATGGGACCTTTTTGTTGAACGCAGCAAAGCTTTCAGCAACTGTTACCCTTATGATGTGCCAGATTATGCCTCCCTTAGGTCACTAGTTGCCTCGTCAGGCACTCTGGAGTTTATCAATGAAGGCTTCAATTGGACTGGAGTCACTCAGAGTGGGGGAAGCTATGCTTGCAAAAGGGGATCTGATAACAGTTTCTTCAGTAGACTGAATTGGTTGTACGAATCAGAAAGCAAATATCCAGTGCTGAACGTGACTATGCCAAACAATGGCAATTTTGACAAACTGTACATTTGGGGGGTTCACCACCCGAGCACGGACAAAGAACAAACCAACCTATATGTTCGAGCATCAGGGAGAGTCACAGTCTCTACCAAGAGAAGCCAGCAAACTATAATCCCGAATATCGGGTCTAGACCCTGGGTAAGGGGTCTGTCTAGTAGAATAAGTATCTATTGGACAATAGTAAAACCGGGAGACATACTGTTAATTAATAGTAATGGGAACCTAATTGCTCCTCGGGGTTACTTCAAAATACGCACTGGGAAAAGCTCAATAATGAGGTCAGATGCACCTATTGGCACCTGCAGTTCTGAATGCATCACTCCAAATGGAAGCATTCCCAATGACAAGCCCTTTCAAAACGTAAACAAGATCACATATGGGGCATGTCCCAAGTATGTTAAGCAAAACACTCTGAAGTTGGCAACAGGGATGCGGAATGTACCAGAGAAACAAACTAGA

A/HongKong/46/80

CAAAACCTTCCCGGAAATGACAACAGCACAGCAACGCTGTGCCTGGGACATCATGCAGTGCCAAACGGAACGCTAGTGAAAACAATCACGAATGATCAGATTGAAGTGACTAATGCTACTGAGCTGGTTCAGAGTTCCTCAACAGGTAGAATATGCGACAGTCCTCACCGAATCCTTGATGGGAAAAACTGCACACTGATAGATGCTCTATTGGGAGACCCTCATTGTGATGGCTTTCAAAATGAGAAATGGGACCTTTTTGTTGAACGCAGCAAAGCTTTCAGCAACTGTTACCCTTATGATGTGCCAGATTATGCCTCCCTTAGGTCACTAGTTGCCTCGTCAGGCACCCTGGAGTTTATCAATGAAGGCTTCAATTGGACTGGGGTCACTCAGAGTGGGGGAAGCTATGCTTGCAAAAGGGGATCTGATAAAAGTTTCTTCAGTAGACTGAACTGGTTGTACGAATCAGAAAGCAAATATCCAGCGCTGAACGTGACTATGCCAAACAATGGCAATTTTGACAAACTGTACATTTGGGGGGTTCATCACCCGAGCACGGACAAAGAACAAACCAACCTATATGTTCGAGCATCAGGGAGAGTCACAGTCTCTACCAAGAGAAGCCAGCAAACTATAATCCCGAATATCGGGCCTAGACCCTGGGTAAGGGGCCTGTCTAGTAGAATAAGTATCTATTGGACAATAGTAAAACCGGGAGACATACTGTTAATTAATAGTAGTGGGAACCTAATTGCTCCTCGGGGTTATTTCAAAATACGCACTGGAAAAAGCTCAATAATGAGGTCAGATGCACCTATTGGCACCTGCAGTTCTGAATGCATCACTCCAAATGGAAGCATTCCCAATGACAAGCCCTTCCAAAACGTAAACAAGATCACATATGGGGCATGTCCCAAGTATGTTAAGCAAAACACTCTGAAATTGGCAACAGGGATGCGGAATATACCAGAGAAACAAACTAGA

A/Baylor2A/81

CAAAACCTTCCCGGAAATGACAACAGCACAGCAACGCTGTGCCTGGGACACCATGCAGTGCCAAACGGAACGCTAGTGAAAACAATCACGAATGATCAGATTGAAGTGACTAATGCTACTGAGCTGGTTCAGAGTTCCTCAACAGGTAGAATATGCGACAGTCCTCACCGAATCCTTGATGGGAAAAACTGCACACTGGTAGATGCTCTATTGGGAGACCCTCATTGTGATGGCTTTCAAAATGAGAAATGGGACCTTTTTGTTGAACGCAGCAAAGCTTTCAGCAACTGTTACCCTTATGATGTGCCAGATTATGCCTCCCTTAGATCACTAGTTGCCTCGTCAGGCACCCTGGAGTTTATCAATGAAAGCTTCAATTGGACTGGAGTCACTCAGAGTGGGGGAAGCTATGCTTGCAAAAGGGGATCTGATAACAGTTTCTTCAGTAGACTGAATTGGTTGTACGAATCAGAAAGCAAATATCCAGTGCTGAACGTGACTATGCCAAACAATGGCAATTTTGACAAACTGTACATTTGGGGGGTTCACCACCCGAGCACGGACAAAGAACAAACCAACCTATATGTTCGAGCATCAGGGAGAGTCACAGTATCTACCAAGAGAAGCCAGCAAACTATAATCCCGAATATCGGGTCTAGACCCTGGGTAAGGGGTCTGTCTAGCAGAATAAGCATCTATTGGACTATAGTAAAGCCGGGAGACATACTGTTAATTAATAGTAATGGGAACCTAATTGCTCCTCGGGGTTACTTCAAAATACGCACTGGGAAAAGCTCAATAATGAGGTCAGATGCACCTATTGGCACCTGCAGTTCTGAATGCATCACTCCAAATGGAAGCATTCCCAATGACAAGCCCTTTCAAAATGTAAACAAGATCACATATGGGGCATGTCCCAAGTATGTTAAGCAAAACACTCTGAAGTTGGCAACAGGGATGCGGAATGTACCAGAGAAACAAACTAGA

A/ChristHospital/231/82

CAAAACCTTCCCGGAAATGACAACAGCACAGCAACGCTGTGCCTGGGACACCATGCAGTGCCAAACGGAACGCTAGTGAAAACAATCACGAATGATCAGATTGAAGTGACTAATGCTACTGAGCTGGTTCAGAGTTCCTCAACAGGTAAAATATGCGGCAGTCCTCACCGAATCCTTGATGGGAAAAACTGCACACTGGTAGATGCTCTATTGGGAGACCCTCATTGTGATGGCTCTCAAAATGAGAAATGGGACCTTTTTGTTGAACGCAGCAAAGCTTTCAGCAACTGTTACCCTTATGATGTGCCAGATTATGCCTCCCTTAGGTCACTAGTTGCCTCGTCAGGCACCCTGGAGTTTATCAATGAAAGCTTCAATTGGACTGGAGTCACTCAGAGTGGGGGAAGCTATGCTTGCAAAAGGGGATCTGATAACAGTTTCTTCAGTAGACTGAATTGGTTGTATGAATCAGAAAGCAAATATCCAGTGCTGAACGTGACTATGCCAAACAATGGCAATTTTGACAAACTGTACATTTGGGGGGTTCACCACCCGAGCACGGACAAAGAACAAACCAAACTATATGTTCGAGCATCAGGGAGAGTCACAGTCTCTACCAAGAGAAACCAGCAAACTGTAATCCCGAATATCGGGTCTAGACCCTGGGTAAGGGGTCTGTCTAGCAGAATAAGCATCTATTGGACAATAGTAAAACCGGGAGACATACTGCTAATTAATAGTAATGGGAACCTAATTGCTCCTCGGGGTTACTTCAAAATACGCAATGGGAAAAGCTCAATAATGAGGTCAGATGCACCTATTGGCACCTGCAGTTCTGAATGCATCACTCCAAATGGAAGCATTCCCAATGACAAGCCCTTTCAAAATGTAAACAAGATCACATATGGGGCATGTCCCAAGTATGTTAAGCAAAACACTCTGAAGTTGGCAACAGGGATGCGGAATGTACCAGAAAAACAAACTAGA

A/HongKong/26/83

CAAAAACTTCCCGGAAATGACAACAGCACAGCAACGCTGTGCTTGGGACATCATGCAGTGCCAAACGGAACGCTAGTGAAAACAATCACGAATGATCAGATTGAAGTGACTAATGCTACTGAGCTGGTTCAGATTTCCTCAACAGGTAGAATATGCGACAGTCCTCACCGAATCCTTGATGGGAAAAACTGCACACTGATAGATGCTCTATTGGGAGACCCTCATTGTGATGGCTTCCAAAATGAGAAATGGGACCTTTTTGTTGAACGCAGCAAAGCTTTCAGCAACTGTTACCCTTATGATGTGCCGGATTATGCCTCCCTTAGGTCACTAGTTGCCTCATCAGGCACCCTGGAGTTTATCAATGAAGGCTTCAATTGGACTGGAGTCACTCAGAGTGGGGGAAGCTATACTTGCAAAAGGGGATCTGTTAACAGTTTCTTCAGTAGATTGAATTGGTTGTACGAATCAGAAAGCAAATATCCAGTGCTGAACGTGACTATGCCAAACAATGGCAAATTTGACAAATTGTACATTTGGGGGGTTCACCACCCGAGCACGGACAAAGAACAAACCAACCTATATGTTCGAGCATCAGGGAGAGTCACAGTCTCTACCAAGAGAAGCCAGCAAACTGTAATCCCGAATATCGGGTCTAGACCCTGGGTAAGGGGTCTGTCTAGTAGAATAAGTATCTATTGGACAATAGTAAAACCGGGAGACATACTGTTAATTAATAGCACTGGGAACCTAATTGCTCCTCGGGGTTACTTCAAAATACGCACTGGGAAAAGCTCAATAATGAGGTCAGATGCACCTATTGGCACCTGCAGTTCTGAATGCATCACTCCAAATGGAAGTATTCCCAATGACAAACCCTTTCAAAATGTAAACAAGATCACATATGGGGCATGTCCCAGGTATGTTAAGCAAAACACTCTGAAGTTGGCAACAGGGATGCGGAATGTACCAGAGAAACAAACTAGA

A/Texas/18088/84

CAAAAACTTCCCGGAAATGACAACAGCACAGCAACGCTGTGCCTGGGACATCATGCAGTGCCAAACGGAACGCTAGTGAAAACAATCACGAATGATCAGATTGAAGTGACTAATGCTACTGAGCTGGTTCAGAGCTCCTCAACAGGTAGAATATGCGACAGTCCTCACCGAATCCTTGATGGGAAAAACTGCACACTGATAGATGCTCTATTGGGAGACCCTCATTGTGATGGCTTCCAAAATGAGAAATGGGACCTTTTTGTTGAACGCAGCAAAGCTTTCAGCAACTGTTACCCTTATGATGTGCCGGATTATGCCTCCCTTAGGTCACTAGTTGCCTCATCAGGCACCCTGGAATTTACCAATGAAGGCTTCAATTGGACTGGAGTCACTCAGAGTGGGGGAAGCTATGCTTGCAAAAGGGGATCTGTTAACAGTTTCTTCAGTAGATTGAATTGGTTGTACGAATCAGAAAGCAAATATCCAGTGCTGAACGTGACTATGCCAAACAATGGCAAATTTGACAAATTGTACATTTGGGGGGTTCACCACCCGAGCACGGACAAAGAACAAACCAACCTATATGTTCGAGCATCAGGGAGAGTCACAGTCTCTACCAAGAGAAGCCAGCAAACTGTAATCCCGAATATCGGGTCTAGACCCTGGGTAAGGGGTCTGTCTAGTAGAATAAGTATCTATTGGACAATAGTAAAACCGGGAGACATACTGTTAATTAATAGCACTGGGAACCTAATTGCTCCTCGGGGTTACTTCAAAATACGCACTGGGAAAAGCTCAATAATGAGGTCAGATGCACCTATTGGCACCTGCAGTTCTGAATGCATCACTCCGAATGGAAGCATTCCCAATGACAAACCCTTTCAAAATGTAAACAAGATCACATATGGGGCATGTCCCAGGTATGTTAAGCAAAACACTCTGAAGTTGGCAACAGGGATGCGGAATGTACCAGAGAAACAAACTAGA

A/Stockholm/10/85

CAAAAACTTCCCGGAAATGACAACAGCACAGCAACGCTGTGCCTGGGACATCATGCAGTGCCAAACGGAACGCTAGTGAAAACAATCACGAATGATCAGATTGAAGTGACTAATGCTACTGAGCTGGTTCAGAGTTCCTCAACAGGTAGAATATGCGACAGTCCTCACCGAATCCTTGATGGAAAAAACTGCACACTGATAGATGCTCTATTGGGAGACCCTCATTGTGATGGATTCCAAAATGAGAAATGGGACCTTTTTATTGAACGCAGCAAAGCTTTCAGCAACTGTTACCCTTATGATGTGCCGGATTATGCCTCCCTTAGGTCACTAGTTGCCTCATCAGGCACCCTGGAGTTTATCAATGAAGGCTTCAATTGGACTGGAGTCACTCAGAGTGGGGGAAGCTATGCTTGCAAAAGGGGATCTGTTAACAGTTTCTTCAGTAGATTGAATTGGTTGTACGAATCAGAATACAAATATCCAGCGCTGAACGTGACTATGCCAAACAATGGCAAATTTGACAAACTGTACATTTGGGGGGTTCACCACCCGAGCACGGAAAAAGAACAAACCAACCTATATGTTCGAGCATCAGGGAGAGTCACAGTCTCTACCAAGAGAAGCCAGCAAACTGTAACCCCGAATATCGGGTCTAGACCCTGGGTAAGGGGTCTGTCCAGTAGAATAAGCATCTATTGGACAATAGTAAAACCGGGAGACATACTGTTGATTAATAGCACTGGGAACCTAATTGCTCCTCGGGGTTACTTCAAAATACGCACTGGGAAAAGCTCAATAATGAGGTCAGATGCACCTATTGGCACCTGCAGTTCTGAATGCATCACTCCAAATGGAAGCATTCCCAATGACAAACCCTTTCAAAATGTAAACAAGATCACATATGGGGCATGTCCCAGGTATGTTAAGCAAAACACTCTGAAATTGGCAACAGGGATGCGGAATGTACCAGAGAAACAAACTAGA

A/Colorado/2/86

CAGAAACTGCCCGGAAATGACAACAGCACAGCAACGCTGTGCCTGGGACATCATGCAGTGCCAAACGGAACGCTAGTGAAAACAATCACGAATGATCAGATTGAAGTGACTAATGCTACTGAGCTGGTTCAAAGTTCCTCAACAGGTCGAATATGCGACAGTCCTCACCGAATCCTTGATGGAAAAAACTGCACACTGATAGATGCTCTATTGGGAGACCCTCATTGTGATGGCTTCCAAAATGAGAAATGGGACCTTTTTGTTGAACGCAGCAAAGCTTTCAGCAACTGTTACCCTTATGATGTGCCGGATTATGCCTCCCTTAGGTCACTAGTTGCCTCATCAGGCACCCTGGAGTTTATCAATGAAGACTTCAATTGGACTGGAGTCACTCAGAGTGGGGGAAGCTATGCTTGCAAAAGGGAATCTGTTAACAGTTTCTTCAGTAGATTGAATTGGTTGTACGAATCAGAATACAAATATCCAGCGCTGAACGTGACTATGCCAAACAATGGCAAATTTGAAAAATTGTACATTTGGGGGGTTCACCACCCGAGCACGGACAAAGAACAAACCAACCTATATGTTCGAGCATCAGGGAGAGTCACAGTCTCTACCAAGAGAAGCCAGCAAACTGTAATCCCGAATATCGGGTCTAGACCCTGGGTGAGAGGTCTGTCCAGTAGAATAAGCATCTATTGGACAATAGTAAAACCGGGAGACATACTGTTGATTAATAGCACTGGGAACCTAATTGCTCCTCGGGGTTACTTTAAAATACGCACTGGGAAAAGCTCAATAATGAGGTCAGATGCACCCATTGGCACCTGCAGTTCTGAATGCATCACTCCAAATGGAAGCATTCCCAATGACAAACCCTTTCAAAATGTAAACAAGATCACATATGGGGCATGTCCCAGGTATGTTAGGCAAAACACCCTGAAATTGGCAACAGGGATGCGGAATGTACCAGAGAAACAAACTAGA

A/Los_Angeles/1987

CAAAAACTTCCCGGAAATGACAACAGCACAGCAACGCTGTGCCTGGGACATCATGCAGTGCCAAACGGAACGCTAGTGAAAACAATCACGAATGATCAGATTGAAGTGACTAATGCTACTGAGCTGGTTCAGAGTTCCTCAACAGGTAGAATATGCGACAGTCCTCACCGAATCCTTGATGGAAAAAACTGCACACTGATAGATGCTCTATTGGGAGACCCTCATTGTGATGGCTTCCAAAATGAGAAATGGGACCTTTTTGTTGAACGCAGCAAAGCTTACAGCAACTGTTACCCTTATGATGTGCCGGATTATGCCTCCCTTAGGTCACTAGTTGCCTCATCAGGCACCCTGGAGTTTATCAATGAAGACTTCAATTGGACTGGAGTCACTCAGAGTGGGGGAAGCTATGCTTGCAAAAGGGGATCTGTTAACAGTTTCTTCAGTAGATTGAATTGGTTGCACGAATCAGAATACAAATATCCAGCGCTGAACGTGACTATGCCAAACAATGGCAAATTTGACAAATTGTACATTTGGGGGGTTCACCACCCGATCACGGACAGAGAACAAACCAACCTATATGTTCGAGCATCAGGGAGAGTCACAGTCTCTACCAAGAGAAGCCAGCAAACTGTAATCCCGAATATCGGGTCTAGACCCTGGGTAAGGGGTCTGTCTAGTAGAATAAGCATCTATTGGACAATAGTAAAACCGGGAGACATACTGTTGATTAATAGCACCGGGAACCTAATTGCTCCTCGGGGTTACTTCAAAATACGCACTGGGAAAAGCTCAATAATGAGGTCAGATGCACCTATTGGCACCTGCAGTTCTGAATGCATCACTCCAAATGGAAGCATTCCCAATGACAAACCCTTTCAAAATGTAAACAAGATCACATATGGGGCATGTCCCAGGTATGTTAAGCAAAACACTCTGAAATTGGCAACAGGGATGCGGAATGTACCAGAGAAACAAACTAGA

A/Siena/3/1988

CAAAAACTTCCCGGAAATGACAACAGCACAGCAACGCTGTGCCTGGGACATCATGCAGTGCCAAACGGAACGCTAGTGAAAACAATCACGAATGATCAGATTGAAGTGACTAATGCTACTGAGCTGGTTCAGAGTTCCTCAACAGGTAGAATATGCGACAGTCCTCACCGAATCCTTGATGGAAAAAACTGCACACTGATAGATGCTCTATTGGGAGACCCTCATTGTGATGGCTTCCAAAATGAGAAATGGGACCTTTTTGTTGAACGCAGCAAGGCTTACAGCAACTGTTACCCTTATGATGTGCCGGATTATGCCTCCCTTAGGTCACTAGTTGCCTCATCAGGCACCCTGGAGTTTATCAATGAAGACTTCAATTGGACTGGAGTCACTCAGAGTGGGGGAAGCTATTCTTGCAAAAGGGGATCTGTTAACAGTTTCTTCAGTAGATTGAATTGGTTGCACGAATCAGAATACAAATATCCAGCGCTGAACGTGACTATGCCAAACAATGGCAAATTTGACAAATTGTACATTTGGGGGGTTCACCACCCGAGCACGGACAGAGAACAAACCAAACTATATGTTCGAGCATCAGGGAGAGTCACAGTCTCTACCAAGAGAAGCCAGCAAACTGTAATCCCGAATATCGGGTCTAGACCCTGGGTAAGGGGTCTGTCCAGTAGAATAAGCATCTATTGGACAATAGTAAAACCGGGAGACATACTGTTGATTAATAGCACCGGGAACCTAATTGCTCCTCGGGGTTACTTCAAAATACGCACTGGGAAAAGCTCAATAATGAGGTCAGATGCACCTATTGGCACCTGCAGTTCTGAATGCATCACTCCAAATGGAAGCATTCCCAATGACAAACCCTTTCAAAATGTAAACAAGATCACATATGGGGCATGTCCCAGATATGTTAAGCAAAACACTCTGAAATTGGCAACAGGGATGCGGAATGTACCAGAGAAACAAACTAGA

A/Beijing/353/89

CAAAAACTTCCCGGAAATGACAACAGCACAGCAACGCTGTGCCTGGGACATCATGCAGTGCCAAACGGAACGCTAGTGAAAACAATCACGAATGATCAAATTGAAGTGACTAATGCTACTGAGCTGGTTCAGAGTTCCTCAACAGGTAGAATATGCGACAGTCCTCACCGAATCCTTGATGGAAAAAACTGCACACTGATAGATGCTCTATTGGGAGACCCTCATTGTGATGGCTTCCAAAATAAGGAATGGGACCTTTTTGTTGAACGCAGCAAAGCTTACAGCAACTGTTACCCTTATGATGTGCCGGATTATGCCTCCCTTAGGTCACTAGTTGCCTCATCAGGCACCCTGGAGTTTATCAATGAAGACTTCAATTGGACTGGAGTCGCTCAGAGTGGGGAAAGCTATGCTTGCAAAAGGGGATCTGTTAAAAGTTTCTTTAGTAGATTGAATTGGTTGCACGAATCAGAATACAAATATCCAGCGCTGAACGTGACTATGCCAAACAATGGCAAATTTGACAAATTGTACATTTGGGGGGTTCACCACCCGAGCACGGACAGAGAACAAACCAACCTATATGTTCGAGCATCAGGGAGAGTCACAGTCTCTACCAAAAGAAGCCAACAAACTGTAATCCCGAATATCGGGTCTAGACCCTGGGTAAGGGGTCTGTCCAGTAGAATAAGCATCTATTGGACAATAGTAAAACCGGGAGACATACTTTTGATTAATAGCACCGGGAACCTAATTGCTCCTCGGGGTTACTTCAAAATACGAACTGGGAAAAGCTCAATAATGAGGTCAGATGCACCCATTGGCACCTGCAGTTCTGAATGCATCACTCCAAATGGAAGCATCCCCAATGACAAACCTTTTCAAAATGTAAACAGGATCACATATGGGGCATGTCCCAGATATGTTAAGCAAAACACTCTGAAATTGGCAACAGGGATGCGGAATGTACCAGAGAAACAAACTAGA

A/Siena/10/1990

CAAAAACTTCCCGGAAATGACAACAGCACAGCAACGCTGTGCCTAGGACATCATGCAGTGCCAAACGGAACGCTAGTGAAAACAATCACGAATGACCAAATTGAAGTGACTAATGCTACTGAGCTGGTTCAGAGTTCCTCAACAGGTCGAATATGCGACAGTCCTCACCGAATCCTTGATGGAAAAAACTGCACACTGATAGATGCTCTATTGGGAGACCCTCATTGTGATGGCTTCCAAAATAAGGAATGGGACCTTTTTGTTGAACGCAGCAAAGCTTACAGCAACTGTTACCCTTATGATGTGCCGGATTATGCCTCCCTTAGGTCACTAGTTGCCTCATCAGGCACCCTGGAGTTTATCAATGAAGATTTCAATTGGACTGGAGTCGCTCAGAGTGGGGGAAGCTATGCTTGCAAAAGGGGATCTGTTAACAGTTTCTTCAGTAGATTGAATTGGTTGCACGAATCAGAATACAAATATCCAGCGCTGAACGTGACTATGCCAAACAATGGCAAATTTGACAAATTGTACATTTGGGGGGTTCACCACCCGAGCACGGACAGAGAACAAACCAAACTATATGTTCGAGCATCAGGGAGAGTCACAGTCTCTACCAAAAGAAGCCAGCAAACTGTAATCCCGAATATTGGGTCTAGACCCTGGGTAAGGGGTCTGTCCAGTAGAATAAGCATCTATTGGACAATAGTAAAACCGGGAGACATACTGTTGATTAATAGCACCGGGAACCTAATTGCTCCTCGGGGTTACTTCAAAATACGAACTGGAAAAAGCTCAATAATGAGGTCAGATGCACCCATTGGCACCTGCAGTTCTGAATGCATCACTCCAAATGGAAGCATTCCCAATGACAAACCCTTTCAAAATGTAAACAGGATCACATATGGGGCATGTCCCAGATATGTTAAGCAAAACACTCTGAAATTGGCAACAGGGATGCGGAATGTACCAGAGAAACAAACTAGA

A/Canberra/1/91

CAAAAACTTCCCGGAAATGACAACAGCACAGCAACGCTGTGCCTGGGACATCATGCAGTGCCAAACGGAACGCTAGTGAAAACAATCACGAATGACCAAATTGAAGTGACTAATGCTACTGAGCTGGTTCAGAGTTCCTCAACAGGTCGAATATGCGACAGTCCTCACCGAATCCTTGATGGAAAAAACTGCACACTGATAGATGCTCTATTGGGAGATCCTCATTGTGATGGCTTCCAAAATAAGGAATGGGACCTTTTTGTTGAACGCAGCAAAGCTTACAGCAACTGTTACCCTTATGATGTGCCGGATTATGCCTCCCTTAGGTCACTAGTTGCCTCATCAGGCACCCTGGAGTTTATCAATGAAGACTTCAATTGGACTGGAGTCGCTCAGAGTGGGGGAAGCTATACTTGCAAAAGGGGATCTGTTAACAGTTTCTTCAGTAGATTGAATTGGTTGCACGAATCAGAATACAAATATCCAGCGCTGAACGTGACTATGCCAAACAATGGCAAATTTGACAAATTGTACATTTGGGGGGTTCACCACCCGAGCACGGACAGAGAACAAACCAACCTATATGTTCGAGCATCAGGGAGAGTCACAGTCTCCACCAAAAGAAGCCAGCAAACTGTAATCCCGAATATCGGGTCTAGACCCTGGGTAAGGGGTCTGTCCAGTAGAATAAGCATCTATTGGACAATAGTAAAACCGGGAGACATACTGTTGATTAATAGCACCGGGAACCTAATTGCTCCTCGGGGTTACTTCAAAATACGAACTGGAAAAAGCTCAATAATGAGGTCAGATGCACCCATTGGCACCTGCAGTTCTGAATGCATCACTCCAAATGGAAGCATTTCCAATGACAAACCCTTTCAAAATGTAAACAGGATCACATATGGGGCATGTCCCAGATATGTTAAGCAAAACACTCTGAAATTGGCAACAGGGATGCGGAATGTGCCAGAAAAACAAACTAGA

A/Stockholm/7/92

CAAAAACTTCCCGGAAATGACAACAGCACAGCAACGCTGTGCCTGGGGCATCATGCAGTGCCAAACGGAACTCTAGTGAAAACAATCACGAATGATCAAATTGAAGTGACTAATGCTACTGAGCTGGTTCAGAGTTCCTCAACAGGTAGAATATGCGACAGTCCTCACCGAATCCTCGATGGAAAAAACTGCACACTGATAGATGCTCTATTGGGAGACCCTCATTGTGATGGCTTCCAAAATAAGGAATGGGACCTTTTTGTTGAACGCAGCAAAGCTTACAGCAATTGTTACCCTTATGATGTGCCAGATTACGCCTCCCTTAGGTCACTAGTTGCCTCATCAGGCACCCTGGAGTTTATCAATGAAGACTTCAATTGGACTGGAGTCGCTCAGAATGGGGACAGCTATGCTTGCAAAAGGGGATCTGTTAAAAGTTTCTTTAGTAGATTGAATTGGTTGCACGAATCAGAATACAAATATCCAGCGCTGAACGTGACTATGCCAAACAATGACAAATTTGACAAGTTGTACATTTGGGGGGTTCACCACCCGAGCACGGACAGAGAACAAACCAGCCTATATATTCGAGCATCAGGGAGAGTCACAGTCTCTACCAAAAGAAGCCAACAAACTGTAATCCCGAATATCGGGTCCAGACCCTGGGTAAGGGGTCTGTCCAGTAGAATAAGCATCTATTGGACAATAGTAAAACCGGGAGACATACTTTTGATTAATAGCACCGGGAATCTAATTGCTCCTCGGGGTTACTTCAAAATACGAACTGGGAAAAGCTCGATAATGAGGTCAGATGCACCCATTGGCACCTGCAGTTCTGAATGCATCACTCCAAATGGAAGCATCCCCAATGACAAACCTTTTCAAAATGTAAACAGGATCACATATGGGGCATGTCCCAGATATGTTAAGCAAAACACTCTGAAATTGGCAACAGGGATGCGGAATGTACCAGAGAAACAAACTAGA

A/Argentina/3105/93

CAAAAACTTCCCGGAAATGACAACAGCACAGCAACGCTGTGCCTGGGACACCATGCAGTGCCAAACGGAACGCTAGTGAAAACAATCACGAATGACCAAATTGAAGTGACTAATGCTACTGAGCTGGTTCAGAGTTCCTCAACAGGTAGAATATGCGACAATCCTCACCGAATCCTTGATGGAAAAAACTGCACACTGATAGATGCTCTATTGGGAGACCCTCATTGTGATGGCTTCCAAAATAAGGAATGGGACCTTTTTGTTGAACGCAGCAAAGCTTACAGCAACTGTTACCCTTATGATGTGCCGGATTATGCCTCCCTTAGGTCACTAGTTGCCTCATCAGGCACCCTGGAGTTTATCAATGAAGACTTCAATTGGACTGGAGTCGCTCAGGATGGGAAAAGCTATGCTTGCAAAAGGGGATCTGTTAACAGTTTCTTTAGTAGATTGAATTGGTTGCACAAATTAGAAGACAAGTATCCAGCGCTGAACGTGACTATGCCAAACAATGGCAAATTTGACAAATTGTACATTTGGGGGTTTCACCACCCGAGCACGGACAGTGACCAAACCAGCCTATATGTTCGAGCATCAGGGAGAGTCACAGTCTCTACCAAAAGAAGCCAACAAACTGTAATCCCGAATATCGGGTCTAGACCCTGGGTAAGGGGTCAGTCCAGTAGAATAAGCATCTATTGGACAATAGTAAAACCGGGAGACATACTTTTGATTAATAGCACAGGGAATCTAATTGCTCCTCGGGGTTACTTCAAAATACGAAATGGGAAAAGCTCAATAATGAGGTCAGATGCACCCATTGGCAACTGCAGTTCTGAATGCATCACTCCAAATGGAAGCATTCCCAATGACAAACCTTTTCAAAATGTAAACAGGATCACATATGGGGCCTGCCCCAGATATGTTAAGCAAAACACTCTGAAATTGGCAACAGGGATGCGGAATGTACCAGAGAAACAAACTAGA

A/Argentina/3779/94

CAAAAACTTCCCGGAAATGACAACAGCACAGCAACGCTGTGCCTGGGACACCATGCAGTGCCAAACGGAACGCTAGTGAAAACAATCACGAATGATCAAATTGAAGTGACTAATGCTACTGAGCTGGTTCAGAGTTCCCCAACAGGTAGAATATGCGACAGCCCTCACCGAATCCTTGATGGAAAGAACTGCACACTGATAGATGCTCTATTGGGAGACCCTCATTGTGATGGCTTCCAAAATAAGGAATGGGACCTTTTTGTTGAACGCAGCAAAGCTTACAGCAACTGTTACCCTTATGATGTGCCGGATTATGCCTCCCTTAGGTCACTAGTTGCCTCATCAGGCACCCTGGAGTTTATCAACGAAAACTTCAATTGGACTGGAGTCGCTCAGGATGGGAAAAGCTATGCTTGCAAAAGGGGATCTGTTAACAGTTTCTTTAGTAGATTGAATTGGTTGCACAAATTAGAATACAAATATCCAGCGCTGAACGTGACTATGCCACACAATGGCAAATTTGACAAATTGTACATTTGGGGGGTTCACCACCCGAGCACGGACAGTGTCCAAACCAGCCTATATGTCCGAGCATCAGGGAGAGTCACAGTCTCTACCAAAAGAAGCCAACAAACTGTAATCCCGGATATCGGGTATAGACCATGGGTAAGGGGTCAGTCCAGTAGAATAAGCATCTATTGGACAATAGTAAAACCGGGAGACATACTTTTGATTAATAGCACAGGGAATCTAATTGCTCCTCGGGGTTACTTCAAAATACGAAATGGGAAAAGCTCAATAATGAGGTCAGATGCACCCATTGGCAACTGCAGTTCTGAATGCATCACTCCAAATGGAAGCATTCCCAATGACAAACCTTTTCCAAATGTAAACAGGATCACATATGGGGCCTGCCCCAGATATGTTAAGGAAAACACTCTGAAATTGGCAACAGGGATGCGGAATGTACCAGAGAAACAAACTAGA

A/Netherlands/271/95

CAAAAACTTCCCGGAAATGACAACAGCACAGCAACGCTGTGCCTGGGACACCATGCAGTGCCAAACGGAACGCTAGTGAAAACAATCACAAATGATCAAATTGAAGTGACTAATGCTACTGAGCTGGTTCAGAGTTCCTCAACAGGTAGAATATGCGACAGTCCTCACCGAATCCTTGATGGAAAAAACTGCACACTGATAGATGCTCTATTGGGAGACCCTCATTGTGATGGCTTCCAAAATAAGGAATGGGACCTTTTTGTTGAACGCAGCAAAGCTTACAGCAACTGTTACCCTTATGATGTGCCGGATTATGCTTCCCTTAGGTCACTAGTTGCCTCATCAGGCACCCTGGAGTTTACCAATGAAGGCTTCAATTGGACTGGAGTCGCTCAGGATGGGAAAAGCTATGCTTGCAAAAGGGGATCTGTTAAAGGTTTCTTTAGTAGATTGAATTGGTTGCACAAATTAGAATTCAAATATCCAGCACTGAACGTGACTATGCCAAACAATGACAAATTTGACAAATTGTACATTTGGGGGGTTCACCACCCGAGCACGGACAGTGACCAAACCAGCCTATATGTTCAAGCATCAGGGAGAGTCACAGTCTCTACCAAAAGAAGCCAACAAACTGTAATCCCGAATATCGGGTCCAGACCCTGGGTGAGGGGTATCTCCAGTAGAATAAGCATCTATTGGACAATAGTAAAACCGGGAGACATACTTTTGATTAACAGCACAGGGAATCTAATTGCTCCTCGGGGTTACTTCAAAATACGAAATGGGAAAAGCTCAATAATGAGGTCAGATGCACCCATTGACAACTGCAATTCTGAATGCATCACTCCAAATGGAAGCATTCCCAATGACAAACCTTTTCAAAATGTAAACAGGATCACATATGGGGCCTGTCCCAGATATGTTAAGCAAAACACTCTGAAATTGGCAACAGGGATGCGGAATGTACCAGAGAAACAAACTAGA

A/Germany/491/96

CAAAAACTTCCCGGAAATGACAACAGCACAGCAACGCTGTGCCTGGGACACCATGCAGTGCCAAACGGAACGCTAGTGAAAACAATCACGAATGATCAAATTGAAGTGACTAATGCTACTGAGCTGGTTCAGAGTTCCTCAACAGGTAGAATATGCGACAGTCCTCACCGAATCCTTGATGGAAAAAACTGCACACTGATAGATGCTCTATTGGGAGACCCTCATTGTGATGGCTTCCAAAATAAGGAATGGGACCTTTTTGTTGAACGCAGCAAAGCTTACAGCAACTGTTACCCTTATGATGTGCCGGATTATGCCTCCCTTAGGTCACTAGTTGCCTCATCAGGCACCCTGGAGTTTACCAATGAAGGCTTCAATTGGACTGGAGTCGCTCAGGATGGGAAAAGCTATGCTTGCAAAAGGGGATCTGTTAACAGTTTCTTTAGTAGATTGAATTGGTTGCACAAATTAGAATACAAATATCCAGCACTGAACGTGACTATGCCAAACAATGACAAATTTGACAAATTGTACATTTGGGGGGTTCACCACCCGAGCACGGACAGTGACCAAACCAGCCTATATGTTCAAGCATCAGGGAGAGTCACAGTCTCTACCAAAAGAAGCCAACAAACTGTAATCCCGAATATCGGGTCCAGACCCTGGGTGAGGGGCATCTCCAGTAGAATAAGCATCTATTGGACAATAGTAAAACCGGGAGACATACTTTTGATTACCAGCACAGGGAATCTAATTGCTCCTCGGGGTTACTTCAAAATACGAAATGGGAAAAGCTCAATAATGAGGTCAGATGCACCCATTGACAACTGCAATTCTGAATGCATCACTCCAAATGGAAGCATTCCCAATGACAAACCTTTTCAAAATGTAAACAGGATCACATATGGGGCCTGTCCCAGATATGTTAAGCAAAACACCCTGAAATTGGCAACAGGGATGCGGAATGTACCAGAGAAACAAACTAGA

A/PuertoRico/1/97

CAAAAACTTCCCGGAAATGACAACAGCACGGCAACGCTGTGCCTGGGACACCATGCAGTGCCAAACGGAACGCTAGTGAAAACAATCACGAATGACCAAATTGAAGTGACTAATGCTACTGAGCTGGTTCAGAGTTCCTCAACAGGTAGAATATGCGACAGTCCTCACCGAATCCTTGATGGAGAAAACTGCACACTGATAGATGCTCTATTGGGAGACCCTCATTGTGATGGCTTCCAAAATAAGGAATGGGACCTTTTTGTTGAACGCAGCAAAGCCTACAGCAACTGTTACCCTTATGATGTGCCGGATTATGCCTCCCTTAGGTCACTAGTTGCCTCATCCGGCACCCTGGAGTTTAACAATGAAAGCTTCAATTGGACTGGAGTCGCTCAGAATGGAACAAGCTATGCTTGCAAAAGGAGATCTATTAAAAGTTTCTTTAGTAGATTGAATTGGTTGCACCAATTAAAATACAAATATCCAGCACTGAACGTGACTATGCCAAACAATGACAAATTTGACAAATTGTACATTTGGGGGGTTCACCACCCGAGTACGGACAGTGACCAAACCAGCCTATATGCTCAAGCATCAGGGAGAGTCACAGTCTCTACCAAAAGAAGCCAACAAACTGTAATCCCGAATATCGGATCTAGACCCTGGGTAAGGGGTGTCTCCAGCCGAATAAGCATCTATTGGACAATAGTAAAACCGGGAGACATACTTTTGATTAACAGCACAGGGAATCTAATTGCTCCTCGGGGTTACTTCAAAATACGAAGTGGGAAAAGCTCAATAATGAGGTCAGATGCACCCATTGGCAAATGCAATTCTGAATGCATCACTCCAAATGGAAGCATTCCCAATGACAAACCATTTCAAAATGTAAACAGGATCACATATGGGGCCTGTCCCAGATATGTTAAGCAAAACACTCTGAAATTGGCAACAGGGATGCGGAATGTACCAGAGAAACAAACTAGA

A/Victoria/605/1998

CAAAAACTTCCCGGAAATGACAACAGCACGGCAACGCTGTGCCTGGGACACCATGCAGTGCCAAACGGAACGCTAGTGAAAACAATCACGAATGACCAAATTGAAGTGACTAATGCTACTGAGCTGGTTCAGAGTTCCTCAACAGGTAGAATATGCGACAGTCCTCACCGAATCCTTGATGGAGAAAACTGCACACTGATAGATGCTCTATTGGGAGACCCTCATTGTGATGGCTTCCAAAATAAGGAATGGGACCTTTTTGTTGAACGCAGCAAAGCCTACAGCAACTGTTACCCTTATGATGTGCCGGATTATGCCTCCCTTAGGTCACTAGTTGCCTCATCCGGCACCCTGGAGTTTAACAATGAAAGCTTCAATTGGACTGGAGTCGCTCAGAATGGAACAAGCTCTGCTTGCAAAAGGAGATCTATTAAAAGTTTCTTTAGTAGATTGAATTGGTTGCACCAATTAAAATACAAATATCCAGCACTGAACGTGACTATGCCAAACAATGACAAATTTGACAAATTGTACATTTGGGGGGTTCACCACCCGAGTACGGACAGTGACCAAACCAGCATATATGCTCAAGCATCAGGGAGAGTCACAGTCTCTACCAAAAGAAGCCAACAAACTGTAATCCCGAATATCGGATCTGGACCCTGGGTAAGGGGTGTCTCCAGCAGAATAAGCATCTATTGGACAATAGTAAAACCGGGAGACATACTTTTGATTAACAGCACAGGGAATCTAATTGCTCCTCGGGGTTACTTCAAAATACGAAGTGGGAAAAGCTCAATAATGAGGTCAGATGCACCCATTGGCAAATGCAATTCTGAATGCATCACTCCAAATGGAAGCATTCCCAATGACAAACCATTTCAAAATGTAAACAGGATCACATATGGGGCCTGTCCCAGATATGTTAAGCAAAACACTCTGAAATTGGCAACAGGGATGCGGAATGTACCAGAGAAACAAACTAGA

A/Netherlands/301/99

CAAAAACTTCCCGGAAATGACAACAGCACGGCAACGCTGTGCCTGGGGCACCATGCAGTGCCAAACGGAACGCTAGTGAAAACAATCACGAATGACCAAATTGAAGTGACTAATGCTACTGAGCTGGTTCAGAGTTCCTCAACAGGTAGAATATGCGACAGTCCTCACCAAATCCTTGATGGAGAAAACTGCACACTAATAGATGCTCTATTGGGAGACCCTCATTGTGATGGCTTCCAAAATAAGGAATGGGACCTTTTCGTTGAACGCAGCAAAGCCTACAGCAACTGTTACCCTTATGATGTGCCGGATTATGCCTCCCTTAGGTCACTAGTTGCCTCATCCGGCACACTGGAGTTTAACAATGAAAGCTTCAATTGGACTGGAGTCGCTCAGAATGGAACAAGCTCTGCTTGCAAAAGGAGGTCTAATAAAAGTTTCTTTAGTAGATTGAATTGGTTGCACCAATTAAAATACAAATACCCAGCACTGAACGTGACTATGCCAAACAATGAAAAATTTGACAAATTGTACATTTGGGGGGTTCACCACCCGAGTACGGACAGTGACCAAATCAGCCTATATGCTCAAGCATCAGGGAGAGTCACAGTCTCTACCAAAAGAAGCCAACAAACTGTAATCCCGAATATCGGATCTAGACCCTGGGTAAGGGGTGTCTCCAGCAGAATAAGCATCTATTGGACAATAGTAAAACCGGGAGACATACTTTTGATTAACAGCACAGGGAATCTAATTGCTCCTCGGGGTTACTTCAAAATACGAAGTGGGAAAAGCTCAATAATGAGGTCAGATGCATCCATTGGCAAATGCAATTCTGAATGCATCACTCCAAATGGAAGCATTCCCAATGACAAACCATTTCAAAATGTAAACAGGATCACATATGGGGCCTGTCCCAGATATGTTAAGCAAAACACTCTGAAATTGGCAACAGGGATGCGGAATGTACCAGAGAAACAAACTAGA

A/NewYork/432/2000

CAAAAACTTCCCGTAAATGACAACAGCACGGCAACGCTGTGCCTGGGACACCATGCAGTGCCAAACGGAACGCTAGTGAAAACAATCACGAATGACCACATTGAAGTGACTAATGCTACTGAGCTGGTTCAGAGTTCCTCAACAGGTAGAATATGCGACAGTCCTCACCAAATCCTTGATGGAGAAAACTGCACACTAATAGATGCTCTATTGGGAGACCCTCATTGTGATGGCTTCCAGAATAAGGAATGGGACCTTTTTGTTGAACGCAGCACAGCCTACAGCAACTGTTACCCTTATGATGTGCCGGATTATGCCTCCCTTAGGTCACTAGTTGCCTCATCCGGCACACTGGAGTTTAACAATGAAAGCTTCAATTGGACTGGAGTCGCTCAGAATGGAACAAGCTCTGCTTGCAAAAGGAGATCTATTAAAAGTTTCTTTAGTAGATTGAATTGGTTGCACCAATTAAAATACAAATATCCAGCACTGAACGTGACTATGCCAAACAATGAAAAATTTGACAAATTGTACATTTGGGGGGTTCACCACCCGAGTACGGACAGTGACCAAATCAGCCTATATGCTCAAGCATCAGGGAGAGTCACAGTCTCTACCAAAAGAAGCCAACAAACTGTAATCCCGAATATCGGATCTAGACCCTGGGTAAGGGGTGTCTCCAGCATAATAAGCATCTATTGGACAATAGTAAAACCGGGAGACATACTTTTGATTAACAGCACAGGGAATCTAATTGCTCCTCGGGGTTACTTTAAAATACGAAGTGGGAAAAGCTCAATAATGAGGTCAAATGCACCCATTGGCAAATGCAATTCTGAATGCATCACTCCAAATGGAAGCATTCCCAATGACAAACCATTTCAAAATGTAAACAGGATCACATATGGGGCCTGTCCCAGATATGTTAAGCAAAACACTCTGAAATTGGCAACAGGGATGCGGAATGTACCAGAGAAACAAACTAGA

A/NewYork/85/2001

CAAAAACTTCCCGGAAATGACAACAGCACGGCAACGCTGTGCCTGGGGCACCATGCAGTGCCAAACGGAACGCTAGTGAAAACAATCACGAATGACCAAATTGAAGTGACTAATGCTACTGAGCTGGTTCAGAATTCCTCAACAGGTAGAATATGCGACAGTCCTCACCAAATCCTTGATGGAGAAAACTGCACACTAATAGATGCTCTATTGGGAGACCCTCATTGTGATGGCTTCCAAAATAAGGAATGGGACCTTTTTGTTGAACGCAGCAAAGCCTACAGCAACTGTTACCCTTATGATGTGCCGGATTATGTCTCCCTTAGGTCACTAGTTGCCTCATCAGGCACGCTGGAGTTTAACAATGAAAGCTTCAATTGGACTGGAGTCGCTCAGAATGGGACAAGCTCTGCTTGCAAAAGGAGATCTGATAAAAGTTTCTTTAGTAGATTGAATTGGTTGCACCAATTAAAATACAAATATCCAGCACTGAACGTGACTATGCCAAACAATGAAAAATTTGACAAATTGTACATTTGGGGGGTTCACCACCCGGGTACAGACAATGACCAAATCAGCCTATATGCTCAAGCATCAGGGAGAGTCACAGTCTCTACCAAAAGAAGCCAACAAACTGTAATCCCGAATATTGGATCTGGACCCTGGGTAAGGGGTGTCTCCAGCAGAATAAGCATCTATTGGACAATAGTAAAACCGGGAGACATACTTTTGATTAACAGCACAGGGAATCTAATTGCTCCTCGGGGTTACTTCAAAATACGAAGTGGGAAAAGCTCAATAATGAGGTCAGATGCACCCATTGGCAAATGCAATTCTGAATGCATCACTCCAAATGGAAGCATTCCCAATGACAAACCATTTCAAAATGTAAACAGGATCACATATGGGCCCTGTCCCAGATATGTTAAGCAAAACACTCTGAAATTGGCAACAGGGATGCGGAATGTGCCAGAGAAACAAACTAGA

A/Netherlands/120/02

CAAAAACTTCCCGGAAATGACAACAGCACGGCAACGCTGTGCCTGGGGCACCATGCAGTGCCAAACGGAACGCTAGTGAAAACAATCACGAATGACCAAATTGAAGTAACTAATGCTACTGAGCTGGTTCAGAGTTCCTCAACAGGTAGAATATGCGACAGTCCTCACCAAATCCTTGATGGAGAAAACTGCACACTAATAGATGCTCTATTGGGAGACCCTCATTGTGATGGCTTCCAAAATAAGGAATGGGACCTTTTTGTTGAACGCAGCAAAGCCTACAGCAACTGTTACCCTTATGATGTGCCGGATTATGTCTCCCTTAGGTCACTAGTTGCCTCATCAGGCACGCTGGAGTTTAACAATGAAAGCTTCAATTGGACTGGAGTCGCTCAGAATGGAACAAGCTCTGCTTGCAAAAGGAGATCTGATAAAAGTTTCTTTAGTAGATTGAATTGGTTGCACCAATTAAAATACAAATATCCAGCACTGAACGTGACTATGCCAAACAATGAAAAATTTGACAAACTGTACATTTGGGGGGTTCACCACCCGGGTACAGACAGTGACCAAATCAGCCTATATGCTCAAGCATCAGGGAGAGTCACAGTCTCTACCAAAAGAAGCCAACAAACTGTAATCCCGAATATCGGATCTAGACCCTGGGTAAGGGGTGTCTCCAGCAGAATAAGCATCTATTGGACAATAGTAAAACCGGGAGACATACTTTTGATTAACAGCACAGGGAATCTAATTGCTCCTCGGGGTTACTTCAAAATACGAAGTGGGAAAAGCTCAATAATGAGGTCAGATGCACCCATTGGCAAATGCAATTCTGAATGCATCACTCCAAATGGAAGCATTCCCAATGGCAAACCATTTCAAAATGTAAACAGGATCACATATGGGGCCTGTCCCAGATATGTTAAGCAAAACACTCTGAAATTGGCAACAGGGATGCGGAATGTGCCAGAGAAACAAACTAGA

A/New_York/474/2003

CAAAAACTTCCCGGAAATGACAACAGCACGGCAACGCTGTGCCTTGGGCACCATGCAGTACCAAACGGAACGATAGTGAAAACAATCACGAATGACCAAATTGAAGTTACTAATGCTACTGAGCTGGTTCAGATTTCCTCAACAGGTGGAATATGCGACAGTCCTTATCAGATCCTTGATGGAGAAAACTGCACACTAATAGATGCTCTATTGGGAGACCCTCAGTGTGATGGCTTCCAAAATAAGAAATGGGACCTTTTTGTTGAACGCAGCAAAGCCTACAGCAACTGTTACCCTTATGATGTGCCGGATTATGCCTCCCTTAGGTCACTAGTTGCCTCATCCGGCACACTGGAGTTTAACAATGAAAGCTTCGATTGGACTGGAGTCACTCAGAATGGAACAAGCTCTGCTTGCAAAAGGAGATCTAATAAAAGTTTCTTTAGTAGATTGAATTGGTTGACCCACTTAAAATACAAATACCCAGCATTGAACGTGACTATGCCAAACAATGAAAAATTTGACAAATTGTACATTTGGGGGGTTCACCACCCGGGTACGGACAGTGACCAAATCAGCCTATATGCTCAAGCATCAGGAAGAATCACAGTCTCTACCAAAAGAAGCCAACAAACTGTAATCCCGAATATCGGATCTAGACCCAGGGTAAGGGATGTCTCCAGCCGAATAAGCATCTATTGGACAATAGTAAAACCGGGAGACATACTTTTGATTAACAGCACAGGGAATCTAATTGCTCCTCGGGGTTACTTCAAAATACGAAGTGGGAAAAGCTCAATAATGAGATCAGATGCACCCATTGGCAAATGCAATTCTGAATGCATCACTCCAAATGGAAGCATTCCCAATGACAAACCATTTCAAAATGTAAACAGGATCACATATGGGGCCTGTCCCAGATATGTTAAGCAAAACACTCTGAAATTGGCAACAGGGATGCGAAATGTACCAGAGAAACAAACTAGA

A/Johannesburg/30/2004

CAAAAACTTCCCGGAAATGACAACAGCACGGCAACGCTGTGCCTTGGGCACCATGCAGTACCAAACGGAACGATAGTGAAAACAATCACGAATGACCAAATTGAAGTTACTAATGCTACTGAGCTGGTTCAGAGTTCCTCAACAGGTGGAATATGCGACAGTCCTCATCAGATCCTTGATGGAGAAAACTGCACACTAATAGATGCTCTATTGGGAGACCCTCAGTGTGATGGCTTCCAAAATAAGAAATGGGACCTTTTTGTTGAACGCAGCAAAGCCTACAGCAACTGTTACCCTTATGATGTGCCGGATTATGCCTCCCTTAGGTCACTAGTTGCCTCATCCGGCACACTGGAGTTTAACAATGAAAGCTTCAATTGGACTGGAGTCACTCAAAATGGAACAAGCTCTGCTTGCAAAAGGAGATCTAATAAAAGTTTCTTTAGTAGATTGAATTGGTTGACCCATTTAAAATTCAAATACCCAGCATTGAACGTGACTATGCCAAACAATGAAAAATTTGACAAATTGTACATTTGGGGGGTTCACCACCCGGGTACGGACAATGACCAAATCAGCCTATATGCTCAAGCATCAGGAAGAATCACAGTCTCTACCAAAAGAAGCCAACAAACTGTAATCCCGAATATCGGATCTAGACCCAGGGTAAGGGATGTCCCCAGCAGAATAAGCATCTATTGGACAATAGTAAAGCCGGGAGACATACTTTTGATTAACAGCACAGGGAATCTAATTGCTCCTCGGGGTTACTTCAAAATACGAAGTGGGAAAAGCTCAATAATGAGATCAGATGCACCCATTGGCAAATGCAATTCTGAATGCATCACTCCAAATGGAAGCATTCCCAATGACAAACCATTTCAAAATGTAAACAGGATCACATATGGGGCCTGTCCCAGATATGTTAAGCAAAACACTCTGAAATTGGCAACAGGGATGCGAAATGTACCAGAGAAACAAACTAGA

A/Italy/384/2005

CAAAAACTTCCCGGGAATGACAACAGCACGGCAACGCTGTGCCTTGGGCACCATGCAGTACCAAACGGAACGATAGTGAAAACAATCACGAATGACCAAATTGAAGTTACTAATGCTACTGAGCTGGTTCAGAGTTCCTCAACTGGTGGAATATGCGACAGTCCTCATCAGATCCTTGATGGAGAAAACTGCACACTAATAGATGCTCTATTGGGAGACCCTCAGTGTGATGGCTTCCAAAATAAGAAATGGGACCTTTTTGTTGAACGCAGCAAAGCCTACAGCAACTGTTACCCTTATGATGTGCCGGATTATGCCTCCCTTAGGTCACTAGTTGCCTCATCCGGCACACTGGAGTTTAACAATGAAAGCTTCAATTGGACTGGAGTCACTCAAAATGGAACAAGCTCTGCTTGCAAAAGGAGATCTAATAACAGTTTCTTTAGTAGATTGAATTGGTTGACCCACTTAAAATTCAAATACCCAGCACTGAACGTGACTATGCCAAACAATGAAAAATTTGACAAATTGTACATTTGGGGGGTTCACCACCCGGGTACGGACAATGACCAAATCAGCCTATATGCTCAAGCATCAGGAAGAATCACAGTCTCTACCAAAAGAAGCCAACAAACTGTAATCCCGAATATCGGATCTAGACCCAGGGTAAGGGATATCCCCAGCAGAATAAGCATCTATTGGACAATAGTAAAACCGGGAGACATACTTTTGATTAACAGCACAGGGAATCTAATTGCTCCTCGGGGTTACTTCAAAATACGAAGTGGGAAAAGCTCAATAATGAGATCAGATGCACCCATTGGCAAATGCAATTCTGAATGCATCACTCCAAATGGAAGCATTCCCAATGACAAACCATTTCAAAATGTAAACAGGATCACATATGGGGCCTGTCCCAGATATGTTAAGCAAAACACTCTGAAATTGGCAACAGGGATGCGAAATGTACCAGAGAAACAAACTAGA

A/Thailand/CU23/2006

CAAAAACTTCCCGGAAATGACAACAGCACGGCAACGCTGTGCCTTGGGCACCATGCAGTACCAAACGGAACGATAGTGAAAACAATCACGAATGACCAAATTGAAGTTACTAATGCTACTGAGCTGGTTCAGAGTTCCTCAACAGGTGGAATATGCGACAGTCCTCATCAGATCCTTGATGGAGAAAACTGCACACTAATAGATGCTCTATTGGGAGACCCTCAGTGTGATGGCTTCCAAAATAAGAAATGGGACCTTTTTGTTGAACGCAGCAAAGCCTACAGCAACTGTTACCCTTATGATGTGCCGGATTATGCCTCCCTTAGGTCACTAGTTGCCTCATCCGGCACACTGGAGTTTAACAATGAAAGCTTCAATTGGACTGGAGTCACTCAAAATGGGACAAGCTCTGCTTGCAAAAGGAGATCTAATAACAGTTTCTTTAGTAGATTGAATTGGTTGACCCACTTAAAATTCAAATACCCAGCATTGAACGTGACTATGCCAAACAATGAAAAATTTGACAAATTGTACATTTGGGGGGTTCACCACCCGGGTACGGACAATGACCAAATCTTCCTGTATGCTCAAGCATCAGGAAGAATCACAGTCTCTACCAAAAGAAGCCAACAAACTGTAATCCCGAATATCGGATCTAGACCCAGAGTAAGGAATATCCCCAGCAGAATAAGCATCTATTGGACAATAGTAAAACCGGGAGACATACTTTTGATTAACAGCACAGGGAATCTAATTGCTCCTAGGGGTTACTTCAAAATACGAAGTGGGAAAAGCTCAATAATGAGATCAGATGCACCCATTGGCAAATGCAATTCTGAATGCATCACTCCAAATGGAAGCATTCCCAATGACAAACCATTTCAAAATGTAAACAGGATCACATATGGGGCCTGTCCCAGATATGTTAAGCAAAACACTCTGAAATTGGCAACAGGGATGCGAAATGTACCAGAGAAACAAACTAGA

A/Brisbane/10/2007

CAAAAACTTCCCGGAAATGACAACAGCACGGCAACGCTGTGCCTTGGGCACCATGCAGTACCAAACGGAACGATAGTGAAAACAATCACGAATGACCAAATTGAAGTTACTAATGCTACTGAGCTGGTTCAGAGTTCCTCAACAGGTGAAATATGCGACAGTCCTCATCAGATCCTTGATGGAGAAAACTGCACACTAATAGATGCTCTATTGGGAGACCCTCAGTGTGATGGCTTCCAAAATAAGAAATGGGACCTTTTTGTTGAACGCAGCAAAGCCTACAGCAACTGTTACCCTTATGATGTGCCGGATTATGCCTCCCTTAGGTCACTAGTTGCCTCATCCGGCACACTGGAGTTTAACAATGAAAGCTTCAATTGGACTGGAGTCACTCAAAACGGAACAAGCTCTGCTTGCATAAGGAGATCTAATAACAGTTTCTTTAGTAGATTGAATTGGTTGACCCACTTAAAATTCAAATACCCAGCATTGAACGTGACTATGCCAAACAATGAAAAATTTGACAAATTGTACATTTGGGGGGTTCACCACCCGGGTACGGACAATGACCAAATCTTCCTGTATGCTCAAGCATCAGGAAGAATCACAGTCTCTACCAAAAGAAGCCAACAAACTGTAATCCCGAATATCGGATCTAGACCCAGAGTAAGGAATATCCCCAGCAGAATAAGCATCTATTGGACAATAGTAAAACCGGGAGACATACTTTTGATTAACAGCACAGGGAATCTAATTGCTCCTAGGGGTTACTTCAAAATACGAAGTGGGAAAAGCTCAATAATGAGATCAGATGCACCCATTGGCAAATGCAATTCTGAATGCATCACTCCAAACGGAAGCATTCCCAATGACAAACCATTCCAAAATGTAAACAGGATCACATACGGGGCCTGTCCCAGATATGTTAAGCAAAACACTCTGAAATTGGCAACAGGGATGCGAAATGTACCAGAGAAACAAACTAGA

A/Shanghai/N38/2008

CAAAAACTTCCTGGAAATGACAACAGCACGGCAACGCTGTGCCTTGGGCACCATGCAGTACCAAACGGAACGATAGTGAAAACAATCACGAATGACCAAATTGAAGTTACTAATGCTACTGAGCTGGTTCAGAGTTCCTCAACAGGTGAAATATGCGACAGTCCTCATCAGATCCTTGATGGAGAAAACTGCACACTAATAGATGCTCTATTGGGAGACCCTCAGTGTGATGGCTACCAAAATAAGAAATGGGACCTTTTTGTTGAACGCAGCAAAGCTTACAGCAACTGTTACCCTTATGATGTGCCGGATTATGCCTCCCTTAGGTCACTAGTTGCCTCATCCGGCACACTGGAGTTTAACAATGAAAGCTTCAATTGGACTGGAGTCACTCAAAACGGAACAAGCTCTGCTTGCATAAGGAGATCTAATAACAGTTTCTTTAGTAGATTGAATTGGTTGACCCACTTAAAATTCAAATACCCAGCATTGAATGTGACTATGCCAAACAATGAACAATTTGACAAATTGTACATTTGGGGGGTTCACCACCCGGGTACGGACAATGACCAAATCTTCCTGTATGCTCAAGCATCAGGAAGAATCACAGTCTCTACCAAAAGAAGCCAACAAACTGTAATTCCGAATATCGGATCTAGACCCAGAGTAAGGAATATCCCTAGCAGAATAAGCATCTATTGGACAATAGTAAAACCGGGAGACATACTTTTGATTAACAGCACAGGGAATCTAATTGCTCCTAGGGGTTACTTCAAAATACGAAGTGGGAAAAGCTCAATAATGAAATCAGATGCACCCATTGGCAAATGCAATTCTGAATGCATCACTCCAAATGGAAGCATTCCCAATGACAAACCATTCCAAAATGTAAGCAGGATCACATACGGGGCCTGTCCCAGATATGTTAAACAAAACACTCTGAAATTGGCAACAGGGATGCGAAATGTACCAGAGAAACAAACTAGA

A/Victoria/502/2009

CAAAAACTTCCTGGAAATGACAACAGCACGGCAACGCTGTGCCTTGGGCACCATGCAGTACCAAACGGAACGATAGTGAAAACAATCACGAATGACCAAATTGAAGTTACTAATGCTACTGAGCTGGTTCAGAGTTCCTCAACAGGTGAAATATGCAACAGTCCTCATCAGATCCTTGATGGAGAAAACTGCACACTAATAGATGCTCTATTGGGAGACCCTCAGTGTGATGGCTTCCAAAATAAGAAATGGGACCTTTTTGTTGAACGCAGCAAAGCCTACAGCAACTGTTACCCTTATGATGTGCCGGATTATGCCTCCCTTAGGTCACTAGTTGCCTCATCCGGCACACTGGAGTTTAACAATGAAAGCTTCAATTGGACTGGAGTCACTCAAAACGGAACAAGCTCTGCTTGCATAAGGAGATCTAATAACAGTTTCTTTAGTAGATTGAATTGGTTGACCCACTTAAAATTCAAATACCCAGCATTAAACGTGACTATGCCAAACAATGAACAATTTGACAAATTGTACATTTGGGGGGTTCACCACCCGGGTACGGACAATGACCAAATCTTCCTGTATGCTCAAGCATCAGGAAGAATCACAGTCTCTACCAAAAGAAGCCAACAAACTGTAATCCCGAATATCGGATCTAGACCCAGAGTAAGGAATATCCCTAGCAGAATAAGCATCTATTGGACAATAGTAAAACCGGGAGACATACTTTTGATTAACAGCACAGGGAATCTAATTGCTCCTAGGGGTTACTTCAAAATACGAAGTGGGAAAAGCTCAATAATGAGATCAGATGCACCCATTGGCAAATGCAATTCTGAATGCATCACTCCAAATGGAAGCATTCCCAATGACAAACCATTCCAAAATGTAAACAGGATCACATACGGGGCCTGTCCCAGATATGTTAAGCAAAACACTCTGAAATTGGCAACAGGGATGCGAAATGTACCAGAGAAACAAACTAGA

A/Nanjing/01/2010

CAAAAACTTCCTGGAAATGACAACAGCACGGCAACGCTGTGCCTTGGGCACCATGCAGTCTCAAACGGAACGATAGTGAAAACAATCACGAATGACCAAATTGAAGTTACTAATGCTACTGAGCTGGTTCAGAGTTCCTCAACAGGTGAAATATGCGACAGTCCTCATCAGATCCTTGATGGAGAAAACTGCACACTAATAGATGCTCTATTGGGAGACCCTCAGTGTGATGGCTTCCAAAATAAGAAATGGGACCTTTTTGTTGAACGAAGCAAAGCCTACAGCAACTGTTACCCTTATGATGTGCCGGATTATGCCTCCCTTAGGTCACTAGTTGCCTCATCCGGCACACTGGAGTTTAACAATGAAAGCTTCAATTGGACTGGAGTCACTCAAAACGGAACAAGCTCTGCTTGCATAAGGAGATCTAATAATAGTTTCTTTAGTAGATTGAATTGGTTGACCCACTTAAACTTCAAATACCCAGCATTGAACGTGACTATGCCAAACAATGAACAATTTGACAAATTGTACATTTGGGGGGTTCACCACCCGGGTACGGACAAGGACCAAATCTTCCTGTATGCTCAAGCATCAGGAAGAATCACAGTATCTACCAAAAGAAGCCAACAAGCTGTAATCCCGAATATCGGATCTAGACCCAGAGTAAGGAATATCCCTAGCAGAATAAGCATCTATTGGACAATAGTAAAACCGGGGGACATACTTTTGATTAACAGCACAGGGAATCTAATTGCTCCTAGGGGTTACTTCAAAATACGAAGTGGGAAAAGCTCAATAATGAGATCAGATGCACCCATTGGCAAATGCAATTCTGAATGCATCACTCCAAATGGAAGCATTCCCAATGACAAACCATTCCAAAATGTAAACAGGATCACATACGGGGCCTGTCCCAGATATGTTAAGCAAAACACTCTGAAATTGGCAACAGGAATGCGAAATGTACCAGAGAAACAAACTAGA
